# Effect of Iboga Alkaloids on µ-Opioid Receptor-Coupled G Protein Activation

**DOI:** 10.1371/journal.pone.0077262

**Published:** 2013-10-16

**Authors:** Tamara Antonio, Steven R. Childers, Richard B. Rothman, Christina M. Dersch, Christine King, Martin Kuehne, William G. Bornmann, Amy J. Eshleman, Aaron Janowsky, Eric R. Simon, Maarten E. A. Reith, Kenneth Alper

**Affiliations:** 1 Department of Psychiatry, New York University School of Medicine, New York, New York, United States of America; 2 Department of Biochemistry and Molecular Pharmacology, New York University School of Medicine, New York, New York, United States of America; 3 Department of Physiology and Pharmacology, Wake Forest School of Medicine, Winston-Salem, North Carolina, United States of America; 4 Translational Pharmacology Research Section, National Institute on Drug Abuse Intramural Research Program, Baltimore, Maryland, United States of America; 5 Department of Chemistry, University of Vermont, Burlington, Vermont, United States of America; 6 Department of Experimental Therapeutics, University of Texas M. D. Anderson Cancer Center, Houston, Texas, United States of America; 7 Research Service, VA Medical Center, and Departments of Psychiatry and Behavioral Neuroscience, Oregon Health and Science University, Portland, Oregon, United States of America; 8 Department of Neurology, New York University School of Medicine, New York, New York, United States of America; Medical School of Hannover, United States of America

## Abstract

**Objective:**

The iboga alkaloids are a class of small molecules defined structurally on the basis of a common ibogamine skeleton, some of which modify opioid withdrawal and drug self-administration in humans and preclinical models. These compounds may represent an innovative approach to neurobiological investigation and development of addiction pharmacotherapy. In particular, the use of the prototypic iboga alkaloid ibogaine for opioid detoxification in humans raises the question of whether its effect is mediated by an opioid agonist action, or if it represents alternative and possibly novel mechanism of action. The aim of this study was to independently replicate and extend evidence regarding the activation of μ-opioid receptor (MOR)-related G proteins by iboga alkaloids.

**Methods:**

Ibogaine, its major metabolite noribogaine, and 18-methoxycoronaridine (18-MC), a synthetic congener, were evaluated by agonist-stimulated guanosine-5´-*O*-(γ-thio)-triphosphate ([^35^S]GTPγS) binding in cells overexpressing the recombinant MOR, in rat thalamic membranes, and autoradiography in rat brain slices.

**Results And Significance:**

In rat thalamic membranes ibogaine, noribogaine and 18-MC were MOR antagonists with functional Ke values ranging from 3 uM (ibogaine) to 13 uM (noribogaine and 18MC). Noribogaine and 18-MC did not stimulate [^35^S]GTPγS binding in Chinese hamster ovary cells expressing human or rat MORs, and had only limited partial agonist effects in human embryonic kidney cells expressing mouse MORs. Ibogaine did not did not stimulate [^35^S]GTPγS binding in any MOR expressing cells. Noribogaine did not stimulate [^35^S]GTPγS binding in brain slices using autoradiography. An MOR agonist action does not appear to account for the effect of these iboga alkaloids on opioid withdrawal. Taken together with existing evidence that their mechanism of action also differs from that of other non-opioids with clinical effects on opioid tolerance and withdrawal, these findings suggest a novel mechanism of action, and further justify the search for alternative targets of iboga alkaloids.

## Introduction

**Figure 1:**
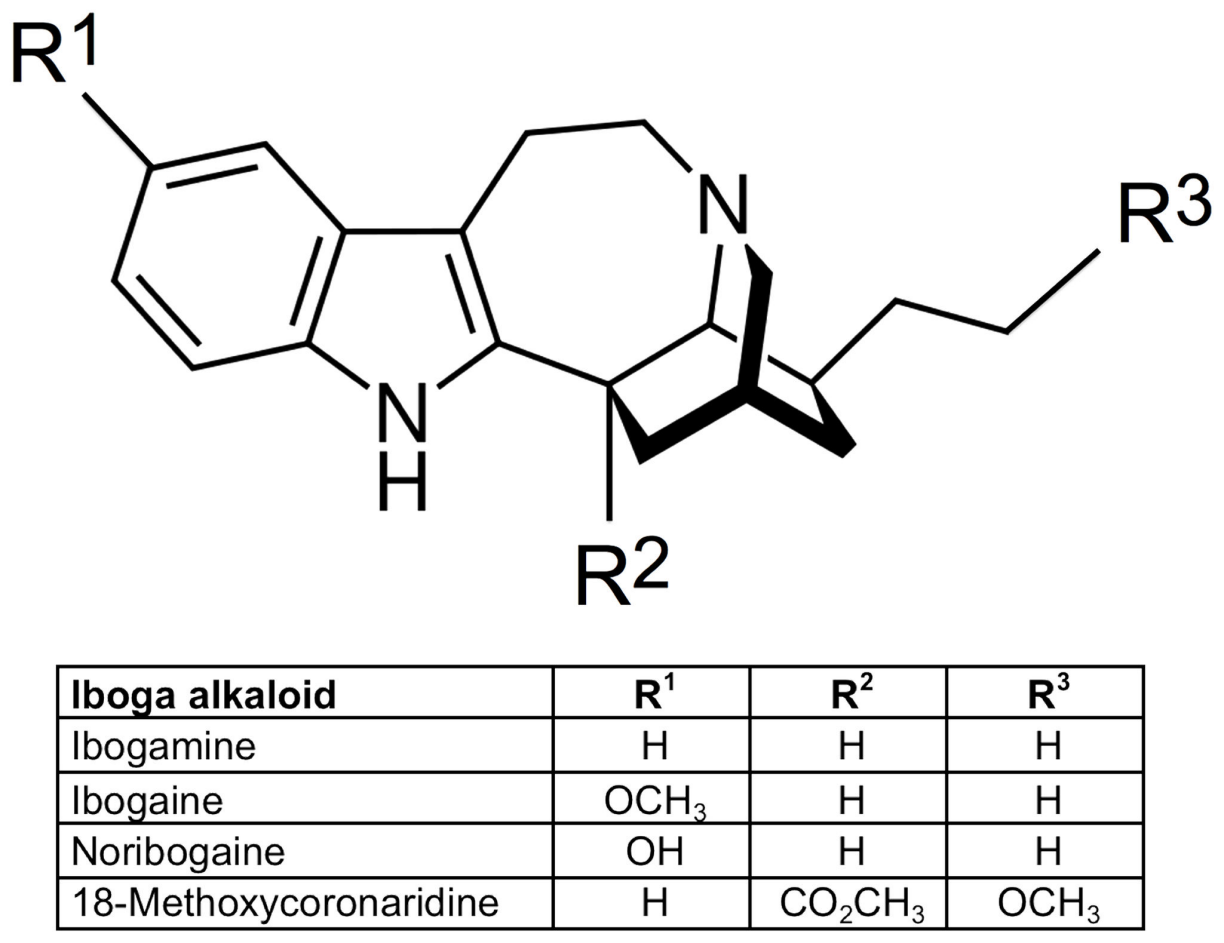
Structures of the iboga alkaloid ibogamine parent skeleton and ibogaine, noribogaine, and 18-MC.

The iboga alkaloids are a class of approximately 80 known naturally occurring and synthetic monoterpene indole alkaloids defined structurally on the basis of a common ibogamine skeleton [1,2] (Fig. 1). As novel small molecules that modify opioid withdrawal and drug self-administration, iboga alkaloids are of interest with regard to their potential for neurobiological investigation and drug development. Ibogaine, the prototypic and presently the most extensively studied iboga alkaloid, occurs in the root bark of the West African shrub *Tabernanthe iboga* Baill. (Apocynaceae family). In West Central Africa *eboga*, crude *T. iboga* root bark, has been used as a sacramental hallucinogen in the Bwiti religion for centuries [3]. In North America, Europe, and elsewhere ibogaine is used in humans in medical and nonmedical settings for treatment of substance use disorders [4-6]. The National Institute on Drug Abuse (NIDA) has recently committed 3.6 million USD support to date for preclinical testing and chemical manufacturing and control work intended to enable clinical trials to develop the synthetic iboga alkaloid 18-methoxycoronaridine (18-MC) as a pharmacotherapy for addiction [7].

Ibogaine has most often been administered as a single large dose in the range of 10 to 25 mg/kg, most often for the specific indication of opioid detoxification [4-6]. Residual effects on self-administration of abused substances in humans or animal models following treatment with iboga alkaloids are a focus of interest for development, however detoxification from opioids with ibogaine presently remains the clinical context in which iboga alkaloids have been most commonly administered to humans as addiction treatment [4]. The clinical phenomenon of opioid detoxification with ibogaine, often in the setting of severe physiological dependence is robust [4-6], and appears to be pharmacologically mediated and not accounted for by placebo effects, which are clinically negligible in opioid detoxification [8-10]. Although the half-life of ibogaine in humans is on the order of 4 to 7 hours [6,11], signs of withdrawal are typically absent following treatment with adequate single doses [4-6]. This is distinct from detoxification with opioid agonists, which generally must be tapered over days or weeks in order to avoid withdrawal signs and symptoms [8-10]. Individuals typically do not go back into withdrawal following opioid detoxification with single doses of ibogaine despite opioid abstinence, suggesting a persistent modification of neuroadaptations associated with opioid tolerance or dependence. Consistent with observations in humans, iboga alkaloids administered intraperitoneally or intracerebrally in preclinical models have reduced naloxone or naltrexone-precipitated opioid withdrawal in thirteen of 14 reported studies in the rat, mouse and two primate species [12-25]. 

Although ibogaine, noribogaine and 18-MC bind to the μ-opioid receptor (MOR) with affinities in the low micromolar range [26-29], functional effects of ibogaine in humans and preclinical models appear to suggest that these iboga alkaloids are not orthosteric MOR agonists. Ibogaine does not itself produce the classical MOR agonist effect of analgesia in preclinical models, although it potentiates morphine analgesia [23,30-35]. Ibogaine and its principal metabolite noribogaine attenuate tolerance to morphine analgesia in mice [33,35,36], and Ciba Pharmaceutical in 1957 obtained a patent on ibogaine for the inhibition of the development of tolerance to morphine analgesia [30,31]. These effects of ibogaine or noribogaine on analgesia and tolerance appear to involve signaling pathways relatively specifically linked to the MOR because they were seen with the administration of morphine, but not with delta or kappa opioid agonists [33,34]. 

Another line of evidence that suggests ibogaine does not act as an orthosteric MOR agonist is that dosages equivalent to those used in opioid detoxification does not produce signs of overdose in individuals who lack tolerance to opioids [5,6], as would be expected if it were a MOR agonist. The oral dose of the MOR agonist methadone that is generally recommended in the maintenance treatment of opioid dependence is in the range 60 to 100mg [37], and substantially exceeds the LD50 of methadone in humans who are not pharmacologically tolerant to opioids, which is estimated to be less than 50 mg [38]. However, doses of ibogaine equivalent to those used to detoxify addicts do not produce opioid overdose in non-tolerant individuals such as Bwiti initiates, or those taking ibogaine for substance use indications other than opioid dependence. Collectively, in vivo evidence suggests that an orthosteric MOR agonist action does not explain the effect of iboga alkaloids in opioid detoxification [26,27,39-42]. 

Although the clinical and in vivo preclinical evidence reviewed above appears to weigh against an opioid agonist effect, there is one report on agonist effects of iboga alkaloids in functionally activating the MOR as assessed by guanosine-5´-*O*-(γ-thio)-triphosphate ([^35^S]GTPγS) binding [43]. Consistent with the apparent lack of in vivo MOR agonist effect, ibogaine stimulated [^35^S]GTPγS binding to only a small extent (~20-30%) at concentrations of 100 µM to 1 mM in rat thalamic membranes [43]. However in this same study, noribogaine, ibogaine’s principal metabolite was determined to be a full MOR agonist on the basis of naloxone-sensitive stimulation of [^35^S]GTPγS binding. Noribogaine, the product of demethylation of ibogaine via hepatic cytochrome P450 2D6 has a half-life that is estimated to be considerably longer than that of the parent compound [6]. This has lead to the suggestion that the relatively slow elimination of noribogaine may function as a prolonged, gradual MOR agonist “self taper”, analogous to the commonly utilized methadone taper in opioid detoxification [43], a hypothesis that assumes an MOR agonist mechanism of action. 

The question of the functional activation of the MOR is fundamental to the mechanism of action of iboga alkaloids. If iboga alkaloids such as ibogaine, noribogaine or 18-MC are not MOR agonists and this is not an explanatory mechanism, then other, possibly novel alternative targets are indicated. The aim of this study was to replicate findings regarding the activation of MOR-related G proteins in multiple independent laboratories, extend that work to cells overexpressing the MOR in addition to rat thalamic membranes, and to include 18-MC. We found that noribogaine and 18-MC have only limited, partial agonist effects in some MOR expressing cells and do not stimulate [^35^S]GTPγS binding at all in others, and ibogaine did not did not stimulate [^35^S]GTPγS binding in any MOR expressing cells. Ibogaine, noribogaine or 18-MC did not activate G-proteins in thalamic membranes. These compounds do not appear to function as MOR agonists, which further supports the rationale for the search for other, novel targets of iboga alkaloids.

## Materials and Methods

### Animal welfare

Animals were handled under Institutional Animal Care and Use Committee (IACUC)-approved protocols and sacrificed under CO_2_ anesthesia. The protocols were under the following committees: The IACUC of the NYU School of Medicine, the Wake Forest University IACUC, and the IACUC of Oregon Health and Science University.

### Chemicals

#### Sources

[^35^S]GTPγS (1,250 Ci/mmole) was purchased from Perkin-Elmer (St. Lo uis, MO, USA). Dulbecco’s Modiﬁed Eagle’s Medium (DMEM) was from Gibco (Grand Island, NY, USA) or from Sigma-Aldrich Chemicals (St. Louis, MO). Dulbecco’s phosphate buffered saline (DPBS), buprenorphine, MgCl_2,_ EDTA, EGTA, guanosine-5´-diphosphate (GDP) and GTPγS were from Sigma-Aldrich (St. Louis, MO, USA). DAMGO ([D-Ala^2^, N-MePhe^4^, Gly-ol]-enkephalin) was purchased from Bachem (Torrance, CA, USA). The National Institute on Drug Abuse (NIDA) Drug Supply Program provided DAMGO, buprenorphine, and morphine for some experiments. Penicillin/streptomycin, fetal bovine serum, fetal calf serum, glutamine, NaCl, Tris, 6 N HCl, sucrose, bovine serum albumin (BSA) and all reagent grade cell culture chemicals were from Fisher Scientiﬁc (Fair Lawn, NJ, USA) or Atlas Biologicals (Ft. Collins, CO).

 Ibogaine HCl was obtained from Slater & Frith Ltd (Wroxham Norwich, UK) (ibogaine source A), or was obtained from National Institute on Drug Abuse Research Resources Drug Supply Program (ibogaine source B). Noribogaine HCl was prepared by conversion from the above ibogaine source A at the Kuehne lab with preparative high-performance liquid chromatography (HPLC) purification at the Bornmann lab (noribogaine source C), or from Slater & Frith Ltd (donated by Phytostan Enterprises, Inc., Montreal, Quebec) (noribogaine source D). 18-MC HCl was from Obiter Research LLC (Champaign, IL). Ibogaine source A was 95% pure and contaminated by approximately 3% of ibogamine HCl with lesser amounts of tabernanthine HCl and ibogaline HCl, and ibogaine source B was 97.2% pure. Impurities of the noribogaine from source C were < 0.1%. Noribogaine source D was 99.6% pure, with the only detectable impurity being ibogamine. 18-MC HCl contained < 0.1% impurity. High-performance liquid chromatography- mass spectrometry (HPLC-MS), gas chromatography-mass spectrometry (GC-MS) graphs and nuclear magnetic resonance (NMR) spectral data of these compounds are provided as Supporting Information Files S1 and S2. The sources of ibogaine and noribogaine used by the four respective collaborating laboratories were: Reith Lab, ibogaine source A and noribogaine source C; Rothman Lab, noribogaine source C; Janowsky Lab ibogaine source B and noribogaine source D; and Childers Lab, noribogaine source D.

Ibogaine, noribogaine, and 18-MC dissolved in water but the concentration in stocks could not exceed 1 mM without drug coming out of solution; with incubation mixtures routinely containing 10% (v/v) stock of 10 times the intended final concentration, the highest drug concentration tested was 10% of 1 mM, i.e. 10^-4^ M.

#### Synthesis and preparative HPLC of noribogaine source C (Bornmann lab)

400 mg of ibogaine HCl (source A above) was demethylated with boron tribromide by the Archer procedure [44] at the Kuehne lab to provide 220 mg of crude product which had a reverse phase HPLC-MS composition (retention times/MW+1) of 65% noribogaine (5.6 min/297), 29% ibogaine (10.9 min/311), and 6% ibogamine (11.4 min/281). 

Preparative HPLC was carried out at the Bornmann lab on a Varian Prepstar SD-1 semi-preparative system equipped (UV detection at 250 nm) using a Prep Microsorb-MWC18 column (250 X 41.4 mm; 6μ; 60 Å) with the following solvent system A= 5mM ammonium formate in water and B=acetonitrile and a gradient of 20%B to 44%B over 65 minutes with a flow rate of 20 ml/min. 140 mg of the crude material above was dissolved in 10 ml methanol and filtered. The filtrate was divided into ten 1 ml aliquots for subsequent injection. Once completed the fractions corresponding to the correct mass were collected and freeze dried. Analytic HPLC-MS was performed on an Agilent Accurate-Mass 6200 TOF LC/MS system equipped with an Agilent LC1200 HPLC using a Varian Microsorb-MW C18 column (250 X 4.6 mm; 5 μ) with the following solvent gradient system: A= H_2_O /0.1% TFA and B=acetonitrile/0.1% TFA. 10%B to 95%B over 30 min with a flow rate of 1ml/min. 

#### NMR analysis of noribogaine source C (Bornmann lab)

NMR spectra were recorded on an IBM-Bruker Advance 500 (500 MHz for ^1^H NMR and 125.76 MHz for ^13^C NMR), spectrometers. The chemical structures of ibogamine, ibogaine and noribogaine were confirmed by ^1^H and ^13^C NMR via correlation spectroscopy (COSY) and heteronuclear correlation (HETCOR) analysis. NMR spectroscopy of noribogaine source C confirmed the presence of noribogaine and the absence of any other detectable contaminant structure, consistent with a purity > 99.9%. NMR spectral data for noribogaine source C are provided in Supporting Information Files S1 and S2.

#### Assessment of purity of ibogaine source A, noribogaine source D, and 18-MC (Kuehne lab)

HPLC-MS analysis was performed using an AB Sciex 4000 QTrap (AB Sciex, Framingham, MA) hybrid triple quadrupole/linear ion trap liquid chromatograph-mass spectrometer (LCMS). Positive atmospheric chemical ionization was used as the ionization source. Nebulizer temperature was maintained at 450°C. Nebulizer or auxiliary gas was set at 40, curtain gas flow at 30, and the declustering potential was set to 100. The mass spectrometer was operated in single quadrupole mode, scanning from 200 to 400 da. Analytes were separated using a Shimadzu Prominence high performance (HPLC) system (Shimadzu Scientific Instruments, Columbia, MD) across a water to acetonitrile (ACN) gradient, using 0.1% formic acid as an ion pairing reagent. At the beginning of each run, the mobile phase was held at 15% ACN for 1 min, increased to 40% over 17 min, reduced back to 15% over 1 min, and held at 15% for 12 min. Flow was maintained at 100 μl/min. One μl of each sample was injected onto an Alltech (Grace Alltech, Deerfield IL) Alltima C18 reversed phase HPLC column (150 mm x 1 mm x5 μm). Graphs of the HPLC-MS analyses of noribogaine source D and 18-MC are provided in Supporting Information Files S1 and S2.

GC-MS analysis was performed on a Varian Saturn 2100T gas chromatograph-ion trap mass spectrometer with a Varian 3900 GC and CP-8400 autosampler. The mass spectrometer was operated in positive chemical ionization mode using methanol as the chemical ionization reagent gas. One microliter of sample was injected onto a Phenomenex Zebron ZB-5 (5% phenyl-95% dimethyl-polysiloxane) GC column (30m x 0.25 mm ID x 0.25 um film) with the following chromatographic conditions: injection port temp 270°C; Initial GC temp 50°C; Initial Time 2 min; ramp rate 30°C/min; final temp 310°C; final time 15 min. Graphs of GC-MS analysis of ibogaine source A are provided in Supporting Information Files S1 and S2.

### MOR-expressing cells

#### mMOR-expressing human embryonic kidney (HEK) 293 cells (Reith lab)

HEK 293 cells stably expressing mouse mMORs were kindly donated by Richard Howells [45] and were grown in Dulbecco’s Modified Eagle medium (DMEM) supplemented with 10% fetal bovine serum, 2 mM L-glutamine, 0.25 mg/ml geneticin (G418), 100 µg/mL penicillin, and 100 µg/mL streptomycin and maintained at 37°C with 5% CO_2_. Cells grown to confluency were dissociated with ice cold phosphate-buffered saline (PBS), centrifuged at 400 g for 5 min, and the pellet was homogenized in ice cold buffer containing 50 mM Tris-HCl and 10 mM EDTA with a Brinkman polytron. The homogenate was centrifuged at 35,000 g at 4°C for 10 min. This process was repeated once more. The final pellet was resuspended in assay buffer A (50 mM Tris-HCl, 100 mM NaCl, 3 mM MgCl_2_, 0.2 mM EGTA, 10 µg/mL saponin and 10 µM GDP pH 7.4) for immediate use or in freezing buffer A (10 mM Tris-HCl, 0.2 mM EDTA and 10% sucrose, pH 7.4) for later use and stored at −80°C. No difference was observed between fresh and frozen preparations, and most experiments reported here were with fresh material. For experiments with the latter, frozen aliquots were thawed and diluted with assay buffer A and subsequently treated as fresh aliquots. Cell suspension aliquots were preincubated with test drugs in a total volume of 1 mL for 15 min at 30°C in a shaking water bath followed by addition of 0.08 nM [^35^S]GTPyS (1,250 Ci/mmole, Perkin Elmer) and allowed to incubate for an additional 45 min. Binding assays were terminated by cold, rapid vacuum filtration onto a Brandel GF/B filtermat using a Brandel 24-pin manual harvester (Biomedical Research & Development Laboratories, Inc., Gaithersburg, MD) followed by four washes with 3 ml ice-cold 50 mM Tris-HCl buffer pH 7.4. A Beckman LS 6500 Multi-Purpose Scintillation Counter, Beckman Coulter, Inc (Fullerton, CA, USA) was used to determine ^35^S radioactivity at 60% efficiency. Nonspecific binding of [^35^S]GTPγS measured in the presence of 10 μM GTPγS was 10% or less of basal binding in the absence of drug. The plateau binding (maximal binding stimulation over basal) with test drug was expressed as percent of maximal binding observed with 10 μM DAMGO unless indicated otherwise (% E_max_); each filter mat used for harvesting and scintillation counting contained varying concentrations of test drug for EC_50_ (concentration producing half maximal stimulation) determination. All assays were performed in triplicate and expressed as mean ± SE. EC_50_ values of the test drugs were estimated by nonlinear logarithmic fitting (logistic model) with Origin 7.0 software (OriginLab Software; North Hampton, MA). The functional K_i_ (equilibrium dissociation constant) of an antagonist derived from experiments with a fixed [agonist] and varying [antagonist] was computed with K_i_ = IC_50_ / (1 + {[ago]/EC_50, ago_}). Ke was calculated as in the Rothman lab detailed in next section.

#### hMOR-expressing Chinese hamster ovary (CHO) cells (Rothman lab)

Human **h**MOR-expressing CHO cells were generated and examined for [^35^S]GTPγS binding as described recently [46]. The procedures were generally as described above for HEK 293-MOR cells with the following exceptions; homogenization of cells was carried out in the presence of a protease inhibitor cocktail, incubation mixtures contained additionally 1 mM DDT, and incubation was initiated with test drugs and [^35^S]GTPγS together for a total time of 3 h at 25°C. Stimulation by test drug was expressed as % of that by 1 µM DAMGO. Ke is the functional K_i_ (equilibrium dissociation constant) of an antagonist and is calculated according to the equation: [Test Drug]/(EC50–2/EC_50–1_ −1), where EC_50–2_ is the EC_50_ value in the presence of the test drug and EC_50–1_ is the value in the absence of the test drug.

#### rMOR-expressing CHO cells (Janowsky lab)

CHO cells expressing the rat rMOR were generously supplied by Drs. David Grandy (Oregon Health and Science University) and Thomas Murray (Creighton University) [47]. Cells were grown to confluency in DMEM supplemented with 10% fetal calf serum and 0.40 mg/ml G418 at 37°C with 10% CO_2_. Cells were grown to confluency and starved of serum for 18 hrs before use in [^35^S]GTPγS binding assays. Assay conditions were adapted from Toll et al., 1998 [48]. For an assay, media was decanted and cells were rinsed in Ca^2+^/Mg^2+^ free phosphate buffered saline (CMF-PBS), scraped into 10mL CMF-PBS, and centrifuged at 1000xg at @ 4°C for 10min. The supernatant was removed and 3mL of GTP buffer (20 mM HEPES, 10 mM MgCl_2_, 100 mM NaCl, 0.2 mM DTT, pH 7.4 at 25°C) was added and cell pellets were homogenized with a polytron. Suspensions were centrifuged at 30,000xg for 15 min, and the process was repeated twice with resuspensions in 10 mL. Membranes were either frozen in fresh buffer for use in later experiments, or used immediately. There were no effects of freezing on radioligand binding (data not shown). The effects of ibogaine and its analogues on [^35^S]GTPγS binding were assessed in the presence of GDP (1 µM) and EDTA (1 mM). For purposes of comparison, concentration response curves with DAMGO were also constructed. GTPγS (10 µM) was used to define non-specific binding. Cell membranes and drugs were incubated for 1 hr at room temperature and aspirated over Perkin Elmer Filtermat A filters using a 96-well Tomtec cell harvester. Radioactivity remaining on the filters was measured using a Wallac microbeta plate reader.

### Rat thalamic membranes

#### Rat thalamic membranes (Reith lab)

Female Sprague-Dawley rats (Charles-River, Wilmington, MA) were sacrificed by decapitation, and the brains were collected in ice-cooled glass dishes. Thalami were rapidly dissected, collected in 20 volumes (w/v) of ice-cold buffer (50 mM Tris-HCl, 100 mM NaCl, 3 mM MgCl_2_, 1mM EGTA, pH 7.4), and homogenized with a Brinkman polytron. The homogenate was centrifuged at 35,000g at 4°C for 10 min. The supernatant was discarded and the pellet was resuspended in assay buffer B (50 mM Tris-HCl, 100 mM NaCl, 3 mM MgCl2, 0.2 mM EGTA, pH 7.4) and centrifuged again. The supernatant was discarded and the final pellet was resuspended in assay buffer B containing 10 μM GDP for a final concentration of 0.2-0.3 mg (original wet weight) per tube or in freezing buffer B (10 mM Tris-HCl and 10% sucrose) for later use and stored at −80°C. Details for assays with fresh or frozen aliquots were as above for the cell experiments with the following exceptions: thalamic membrane suspensions were co-incubated with test drugs and 0.09 nM [^35^S]GTPyS from the start for 1 hr at 30°C; and in experiments with buprenorphine, 1 mg/ml bovine serum albumin (BSA) was present in assay buffer B, and in the buprenorphine dilutions in order to avoid absorption of buprenorphine to the walls of incubation tubes.

#### Rat thalamic membranes (Janowsky lab)

To characterize the effects of ibogaine and its analogues on [^35^S]GTPγS binding to brain membranes, two different methods were used. The first method was adapted from Holstein et al, (2013) [49]. Previously dissected and frozen thalami from Fischer F344 or Sprague Dawley rats (350-450 g) were homogenized with a polytron in 1 ml of ice-cold Tris-EDTA-Dithiothreitol (TED) buffer (5 mM Tris-HCl, 1 mM EDTA, 1 mM DTT, and 10% w/v sucrose, pH 7.4 (at 4°C). The homogenate was centrifuged at 1000xg for 10 min at 4°C. The supernatant was decanted and saved and the pellet was homogenized in 0.5 ml ice-cold TED buffer using a Polytron Homogenizer and centrifuged again at 1000xg. The supernatants were combined and centrifuged at 9,000xg for 20 min at 4°C. The pellet was resuspended in 1 mL TED buffer and centrifuged at 16,000xg for 20 min at 4°C. The procedure with the pellet was repeated. The pellet was resuspended in 1 mL TED buffer and preincubated on ice for 30 min. The suspension was centrifuged at 35,000xg for 10 min and the final pellet was resuspended in 2 mL 50 mM Tris buffer and used in assays or aliquoted and frozen. Thawed membranes (10 µg) were incubated for 60 min at 30°C in 500 μl of 50 mM Tris-HCl (pH 7.4) containing 5 mM MgCl_2_, 100 mM NaCl, 0.1 mM EDTA, 0.2 mM EGTA, 0.2 mM dithiothreitol, 20 μM GDP, 0.2 nM [^35^S]GTPγS.

For comparison, tissue preparation and assays were modified to replicate the methods of Pablo and Mash [43]. This latter method used fewer tissue washes. Additionally, 10 µM GDP (as opposed to 20 µM) was included in the binding assay, but DTT was not. When binding samples were filtered, Tris, as opposed to saline was used to rinse the filters [43] as opposed to saline.

#### Rat thalamic membranes (Childers lab)

For [^35^S]GTPγS binding in Sprague-Dawley rat thalamic membranes, brain regions were dissected on ice and frozen separately at −80°C. Tissue samples were thawed, homogenized with a Tissumizer (Tekmar, Cincinnati OH) in cold membrane buffer (50 mM Tris-HCl, 3 mM MgCl_2_, 1 mM EGTA, pH 7.4) and centrifuged at 48,000 x g for 10 min at 4°C. Pellets were resuspended in membrane buffer and centrifuged again under identical conditions. After the second centrifugation, pellets were homogenized in assay buffer (50 mM Tris-HCl, 3 mM MgCl_2_, 0.2 mM EGTA, 100 mM NaCl, pH 7.7). Assays of mu-activated [^35^S]GTPγS binding included 3 µM DAMGO, 30 µM GDP, 0.05 nM [^35^S]GTPγS, 4 mU/ml adenosine deaminase, 10 µg protein and assay buffer in a final volume of 1 ml. Basal binding was detto ermined in the presence of GDP and absence of drug, and nonspecific binding was assessed in the presence of 10 µM GTPγS. Assays were incubated at 30°C for 2 hr. Reactions were terminated by rapid filtration under vacuum through Whatman GF/B glass fiber filters followed by three washes with 3 ml cold 50 mM Tris-HCl buffer pH 7.7. Bound radioactivity was determined by liquid scintillation spectrophotometry at 95% efficiency for [^35^S] after overnight extraction of the filters in 4 ml ScintiSafe Econo scintillation fluid. Data are reported as mean ± standard error of three separate experiments each performed in triplicate.

#### In vitro autoradiography of agonist-stimulated [^35^S]GTPγS binding in brain sections (Childers lab)

For [^35^S]GTPγS binding in sections, brains from Sprague-Dawley rats were frozen in isopentane at −35°C and stored at −80°C. Coronal sections (20 µm) were cut on a cryostat at −20°C, mounted on gelatin-subbed slides and stored at −80°C. Sections were rinsed in assay buffer (50 mM Tris-HCl, 3 mM MgCl2, 0.2 mM EGTA, 100 mM NaCl, pH 7.4) at 25°C for 10 min, followed by a 15 min preincubation in assay buffer containing 2 mM GDP and 10 mU/ml adenosine deaminase at 25°C. Sections were then incubated in assay buffer with 2 mM GDP, 10 mU/ml adenosine deaminase, 0.04 nM [^35^S]GTPγS, with (stimulated) or without (basal) 3 μM DAMGO at 25°C for 2 h. Slides were rinsed twice in cold Tris buffer (50 mM Tris-HCl, pH 7.0) for 2 min and once in deionized water for 30 s [50]. After drying at room temperature overnight, sections were exposed to film for 72 to 96 h in cassettes containing [^14^C] standards for densitometric analysis. Films were digitized with a Sony XC-77 video camera and analyzed densitometrically using the NIH Image program for Macintosh computers. Values were corrected for [^35^S] based on incorporation of [^35^S] into sections of frozen brain paste as previously described [50] and correction factors were used to convert [^14^C] values to [^35^S] data. Net agonist-stimulated [^35^S]GTPγS binding was calculated by subtracting basal binding from agonist-stimulated binding. Results were obtained from three separate experiments in sections from three individual rats.

## Results

### Effect of ibogaine, noribogaine, and 18-MC compared with MOR agonists DAMGO and morphine, and partial agonist buprenorphine in MOR expressing cells

**Figure 2:**
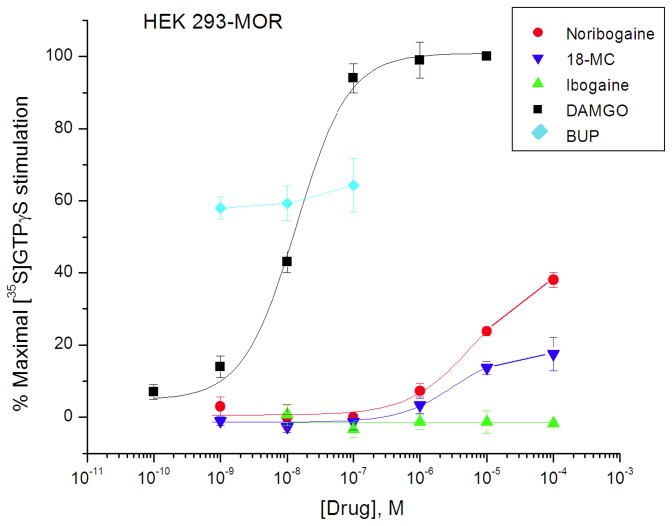
Effect of ibogaine, noribogaine, and 18-MC on [^35^S]GTPγS binding in HEK 293-mMOR cells compared with full agonist DAMGO and partial agonist buprenorphine (BUP) (Reith lab). Cell suspension aliquots were incubated with indicated drug for 15 min and subsequently with an additional concentration of 0.08 nM of [^35^S]GTPγS at 30°C. Data are expressed as % of maximal stimulation by 10 µM DAMGO and presented as mean ± SEM (vertical bar) for 3 - 4 independent experiments assayed in triplicate.

In mMOR-expressing HEK 293 cells (Reith lab), the maximal stimulation of [^35^S]GTPγS binding (0.08 nM) produced by the full agonists DAMGO and morphine respectively was 257 ± 12% and 246 ± 14% over basal (mean ± SEM for 3-4 independent experiments). Using DAMGO (10^-5^ M) as a standard for 100% stimulation, buprenorphine (up to 10^-7^ M) produced a 61 ± 3% increase, consistent with its effect as a partial agonist (Figure 2; Table 1). In comparison, ibogaine, noribogaine, and 18-MC were much less effective. Tested up to final concentrations of 10^-4^ M for solubility reasons (see Methods), noribogaine and 18-MC respectively maximally stimulated 36 ± 6% and 19 ± 4% of maximum DAMGO-stimulated [^35^S]GTPγS binding, and ibogaine showed no stimulation of [^35^S]GTPγS binding (Figure 2; Table 1). EC_50_ values for stimulation for DAMGO, noribogaine and 18-MC were 12.9 ± 2.0 nM, 7.42 ± 1.3 µM, and 3.42 ± 2.2 µM, respectively (Table 1). 

**Table 1:** Effects of iboga alkaloids on [^35^S]GTPγS binding.

**Preparation (lab)**	**Compound**	**Agonist activity**		**Antagonist activity**
		EC_50_ (μM)	E_max_ (%)^1^	Ke (μM)
**Cells expressing MOR**				
HEK mMOR (Reith lab)	Naltrexone	-	-	0.00096 ± 0.00007^3^
	DAMGO	0.0129 ± 0.020	100	-
	Buprenorphine	ND^5^	61 ± 3	-
	Ibogaine^3^	N/A^2^	N/A^2^	4.08 ± 0.55^3^
	Ibogaine^4^	N/A^2^	N/A^2^	1.94 ± 0.46^4^
	Noribogaine	7.42 ± 1.3	36 ± 6	38.3 ± 1.71^4^
	18-MC	3.42 ± 2.2	19 ± 4	19.1 ± 2.97^4^
CHO hMOR (Rothman lab)	DAMGO	0.072 ± 0.09	100	-
	Noribogaine	N/A^2^	N/A^2^	1.48 ± 0.25^4^
CHO rMOR (Janowsky lab)	DAMGO	0.034 ± 0.004	100	-
	ibogaine	N/A^2^	N/A^2^	ND^5^
	Noribogaine	N/A^2^	N/A^2^	ND^5^
	18-MC	N/A^2^	N/A^2^	ND^5^
**Rat thalamic membranes**				
Sprague-Dawley (Reith lab)	DAMGO	0.238 ± 0.024	100	-
	Buprenorphine	0.00016 ± 0.00002	14 ± 2	-
	ibogaine	N/A^2^	N/A^2^	3.05 ± 0.68^4^
	Noribogaine	N/A^2^	N/A^2^	13.3 ± 4.4^4^
	18-MC	N/A^2^	N/A^2^	13.2 ± 3.1^4^
Sprague-Dawley, Fischer 344 (Janowsky lab)	DAMGO	0.122 ± 0.059	100	-
	ibogaine	N/A^2^	N/A^2^	ND^5^
	Noribogaine	N/A^2^	N/A^2^	ND^5^
	18-MC	N/A^2^	N/A^2^	ND^5^
Sprague-Dawley (Childers lab)	DAMGO	0.21 ± 0.33	100	-
	Noribogaine	N/A^2^	N/A^2^	ND^5^
**Autoradiography rat brain sections**				
Sprague-Dawley (Childers lab)	Noribogaine	N/A^2^	N/A^2^	ND^5^

MOR expressing cells or rat thalamus were studied for interaction of iboga alkaloids, and full agonist DAMGO, partial agonist buprenorphine, and antagonist naltrexone as controls, in four different labs. See Results section for full description of the data. Results are given as mean ± SEM for 3-4 independent experiments, except for the MOR cells in the Janowsky lab (n=6) and thalamic membranes in the Janowsky lab (n=2, with measure of error equaling the range).

1 Maximal activity as percent of that elicited by DAMGO (see Methods)

2 No agonist activity detected

3 Versus morphine as agonist

4 Versus DAMGO as an agonist

5 Not determined

In CHO cells expressing hMORs (Rothman lab) and rMORs (Janowsky lab), ibogaine, noribogaine, and 18-MC did not stimulate [^35^S]GTPγS binding to an appreciable extent (Table 1). However, DAMGO stimulated [^35^S]GTPγS binding in these cells, with EC_50_ values of 72 ± 9 nM and 34 ± 4 nM in CHO hMOR and CHO rMOR cells, respectively (Table 1). 

### Antagonism of MOR Agonist Effects by Ibogaine, noribogaine, and 18-MC in MOR Expressing Cells

**Figure 3:**
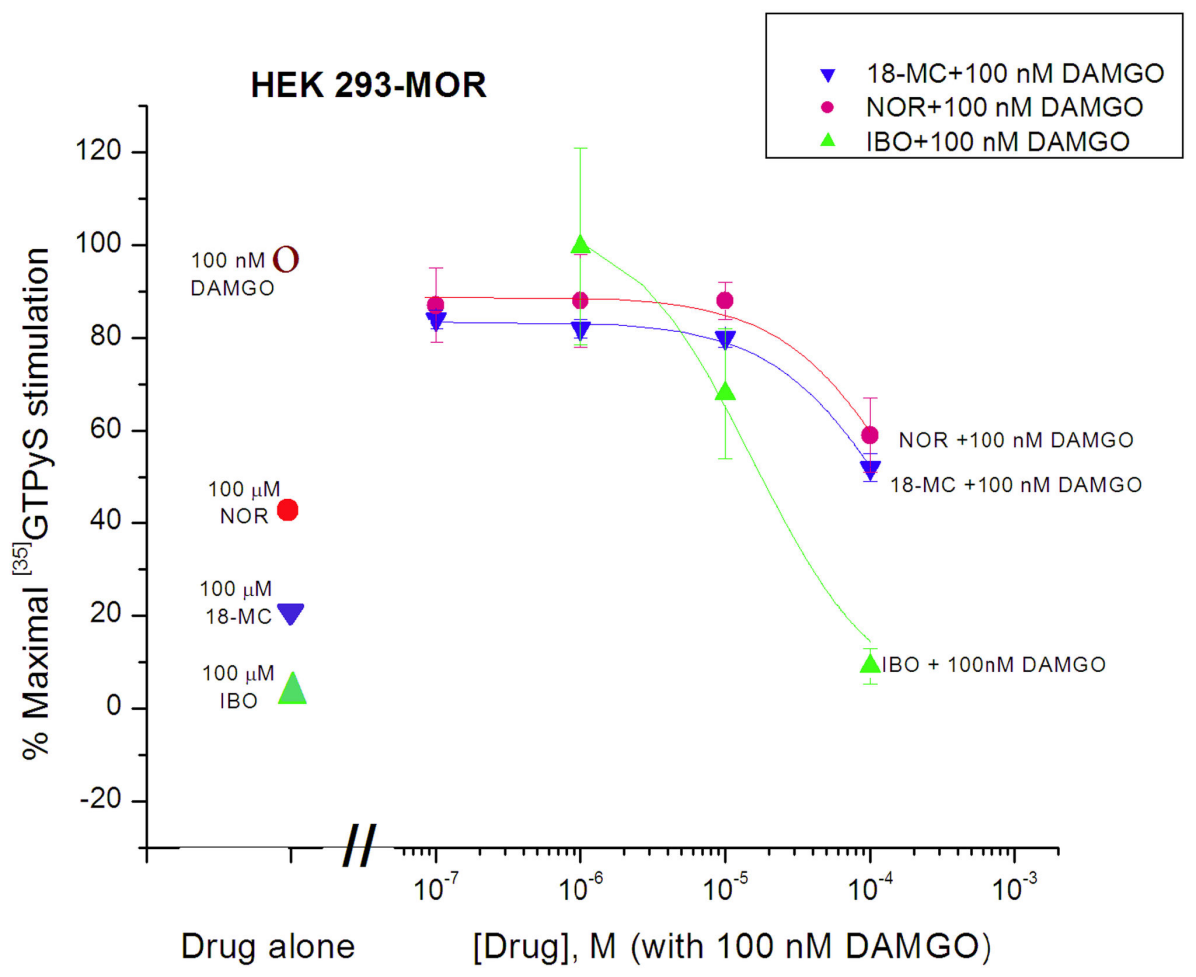
Antagonism of DAMGO (100 nM)-induced [^35^S]GTPγS binding in HEK 293-mMOR cells by ibogaine, noribogaine, and 18-MC (Reith lab). Degree of stimulation by drug alone, i.e.,100 nM DAMGO, 100 µM noribogaine (NOR), 100 µM 18-MC, or 100 µM ibogaine (IBO) is indicated by symbols on the left. The colored curves represent the effect of increasing the concentrations of ibogaine, noribogaine, or 18-MC co-incubated with 100 nM DAMGO. Otherwise as in Figure 2.

**Figure 4:**
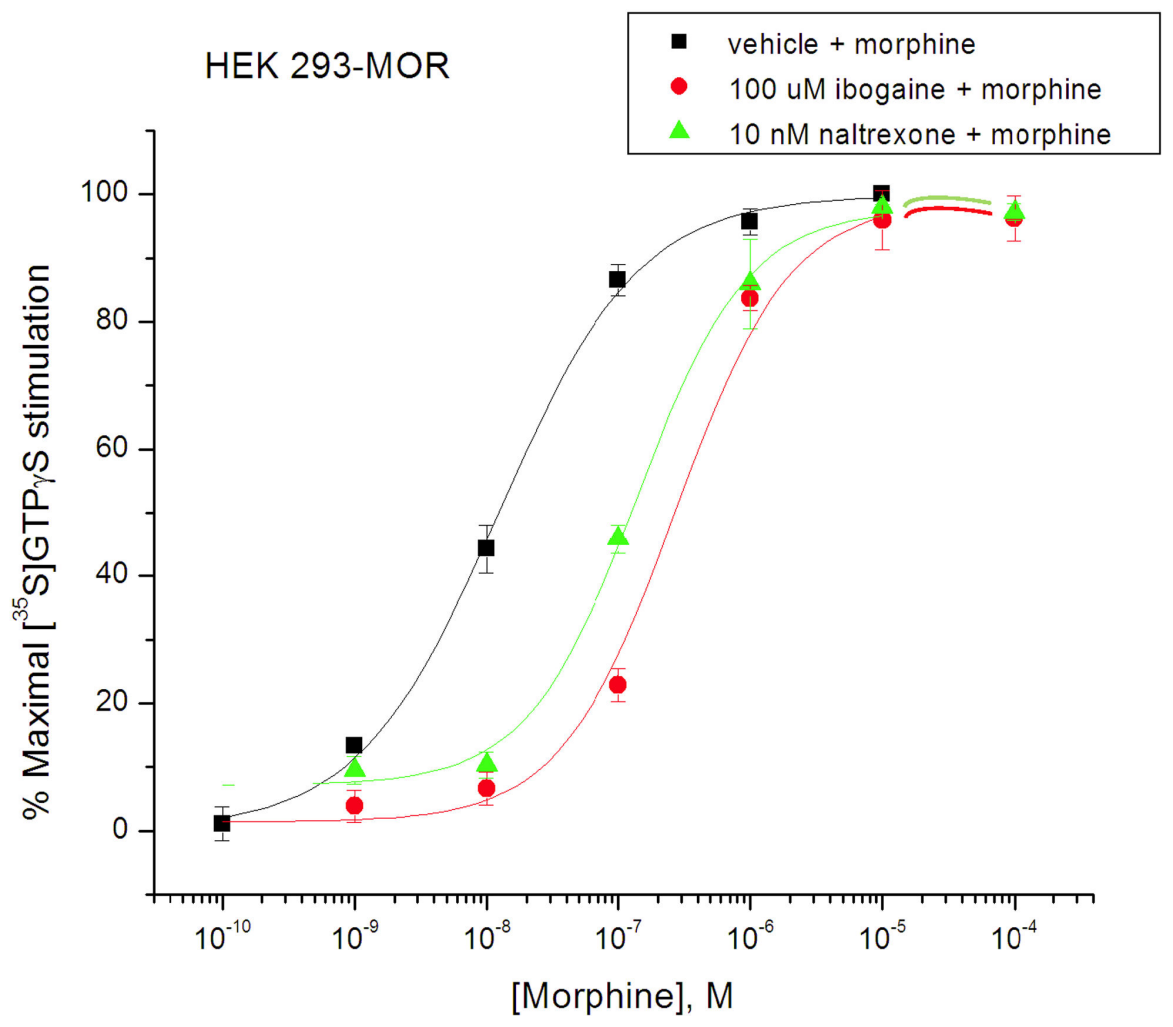
Rightward shift in morphine curves for stimulation of [^35^S]GTPγS binding in HEK 293-mMOR cells by ibogaine (100 µM) or naltrexone (10 nM) (Reith lab). The indicated fixed concentration of ibogaine was co-present with increasing concentrations of morphine (colored curves). Otherwise as in Figure 2 .

Ibogaine by itself did not stimulate [^35^S]GTPγS binding in MOR expressing HEK 293 cells, and when co-incubated with DAMGO (100 nM), it reduced the DAMGO stimulation with an IC_50_ of 17 ± 4 µM (Figure 3), consistent with a Ke value (i.e., functional dissociation constant akin to K_i_ for antagonist) of ibogaine of 1.94 µM (Table 1). Noribogaine also decreased the stimulation by DAMGO to a binding value resembling that of noribogaine alone (Figure 3). The respective IC_50_ values for noribogaine and 18-MC were estimated at 335 ± 15 µM and 167 ± 26 µM, indicating a Ke of 38.3 µM and 19.1 µM, respectively (Table 1). An antagonist effect was also observed when ibogaine (100 µM) was co-incubated with morphine (10^-10^ - 10^-4^ M) (Figure 4). The morphine stimulation curve shifted to the right in the presence of ibogaine, its EC_50_ increased from 12.3 ± 1.2 nM to 314 ± 42 nM, consistent with a Ke value of ibogaine of 4.08 µM (Table 1). Co-incubation with the classical antagonist naltrexone (10 nM) also shifted the morphine curve to the right, resulting in an EC_50_ of 141 ± 10 nM, indicating a Ke value of 0.96 nM (Figure 4; Table 1). The same phenomenon was seen in CHO cells expressing hMOR (Rothman lab); co-incubation with noribogaine (1 µM) reduced the apparent potency of DAMGO in stimulating [^35^S]GTPγS binding, increasing its EC_50_ value consistent with a Ke value for noribogaine of 1.48 µM (Table 1).

### Effect of ibogaine, noribogaine, and 18-MC compared with MOR agonist DAMGO and partial agonist buprenorphine in rat thalamic membranes

**Figure 5:**
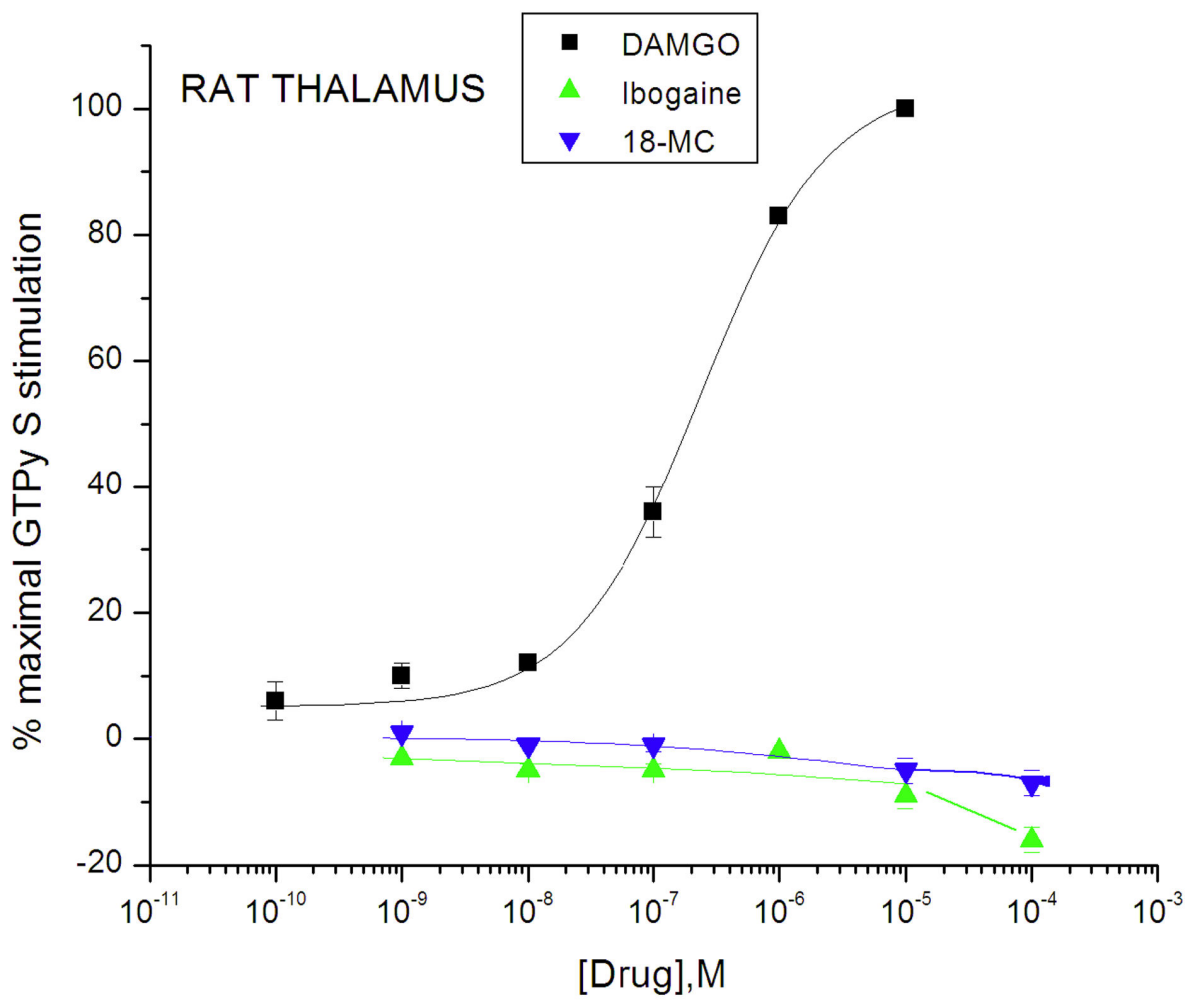
Effect of ibogaine and 18-MC on [^35^S]GTPγS binding in Sprague-Dawley rat thalamic membranes compared with DAMGO (Reith lab). Tissue suspension aliquots were incubated with indicated drug and 0.09 nM of [^35^S]GTPγS for 1 h at 30°C. Data are expressed as % of maximal stimulation by 10 µM DAMGO and presented as mean ± SEM (vertical bar) for 3 - 4 independent experiments assayed in triplicate.

**Figure 6:**
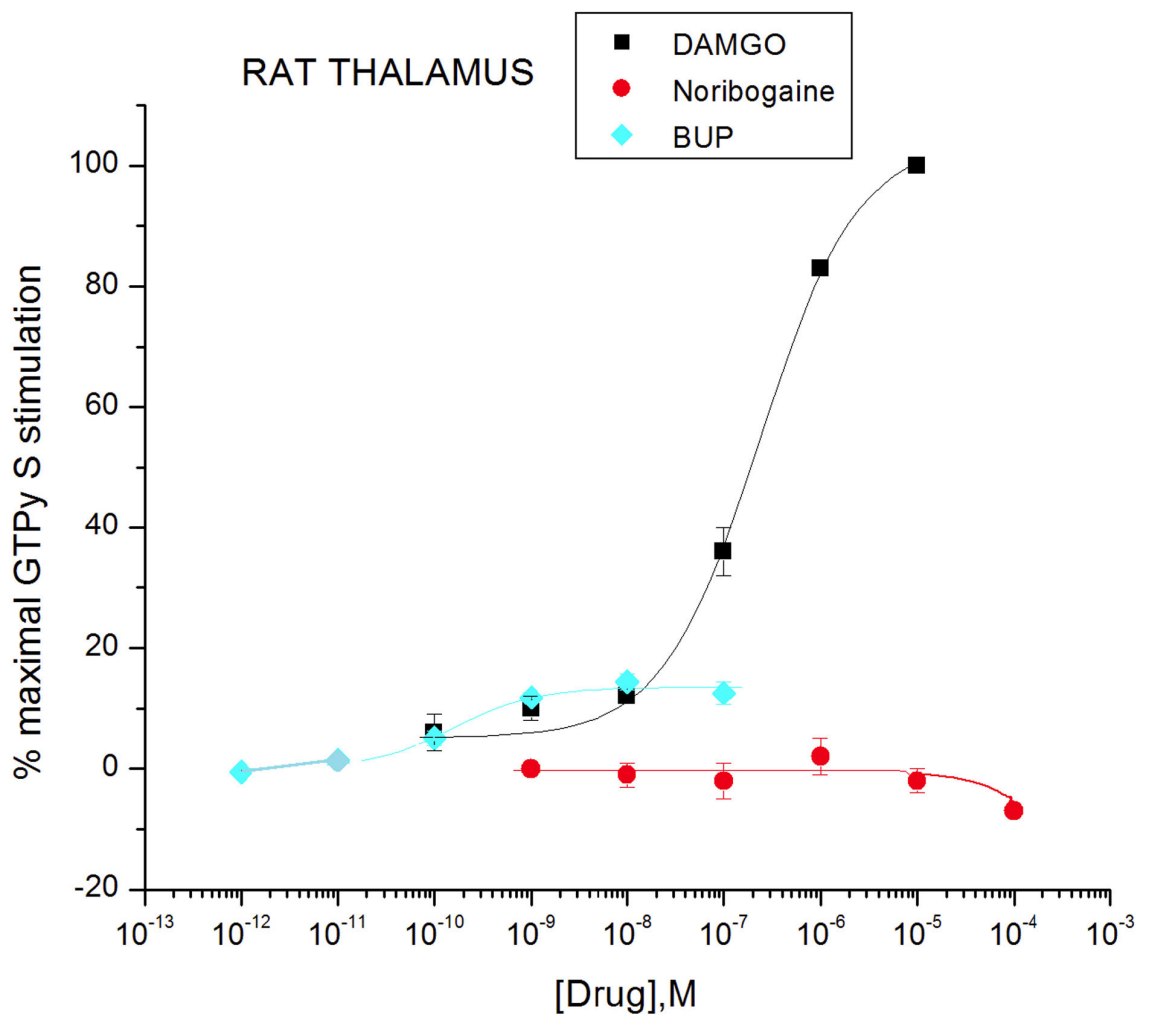
Effect of noribogaine on [^35^S]GTPγS binding in Sprague-Dawley rat thalamic membranes compared with DAMGO and buprenorphine (BUP) (Reith lab). Otherwise as in Figure 5.

**Figure 7:**
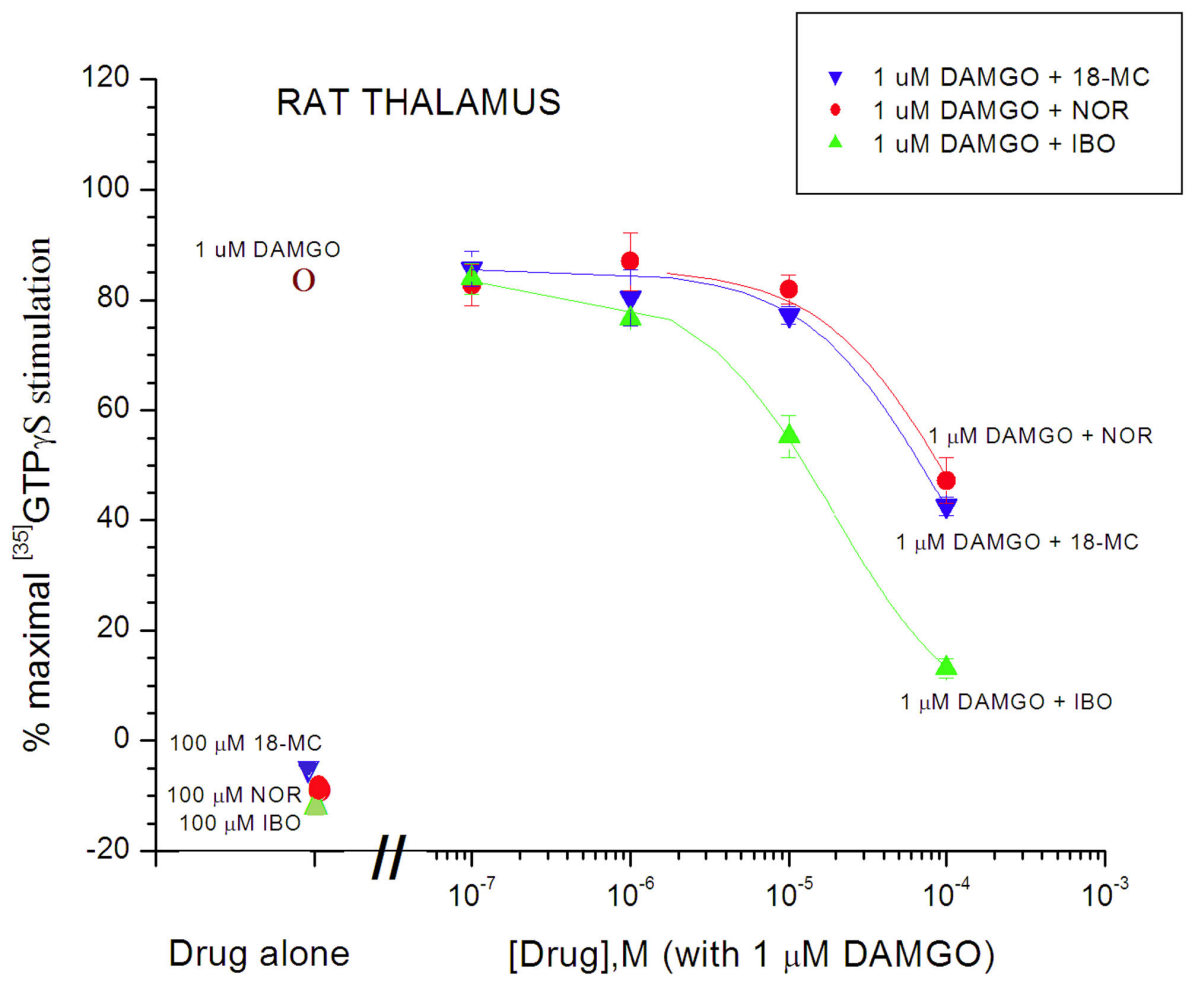
Antagonism of DAMGO (1 µM)-induced [^35^S]GTPγS binding in Sprague-Dawley rat thalamic membranes by ibogaine, noribogaine, and 18-MC (Reith lab). Degree of stimulation by drug alone, i.e., 1 µM DAMGO, 100 µM 18-MC, 100 µM noribogaine (NOR), or 100 µM ibogaine (IBO) is indicated by the symbols on the left. The colored curves represent the effect of increasing the concentration of the respective iboga alkaloids co-incubated with 1 µM DAMGO (5 independent experiments). Otherwise as in Figures 5 and 6.

**Figure 8:**
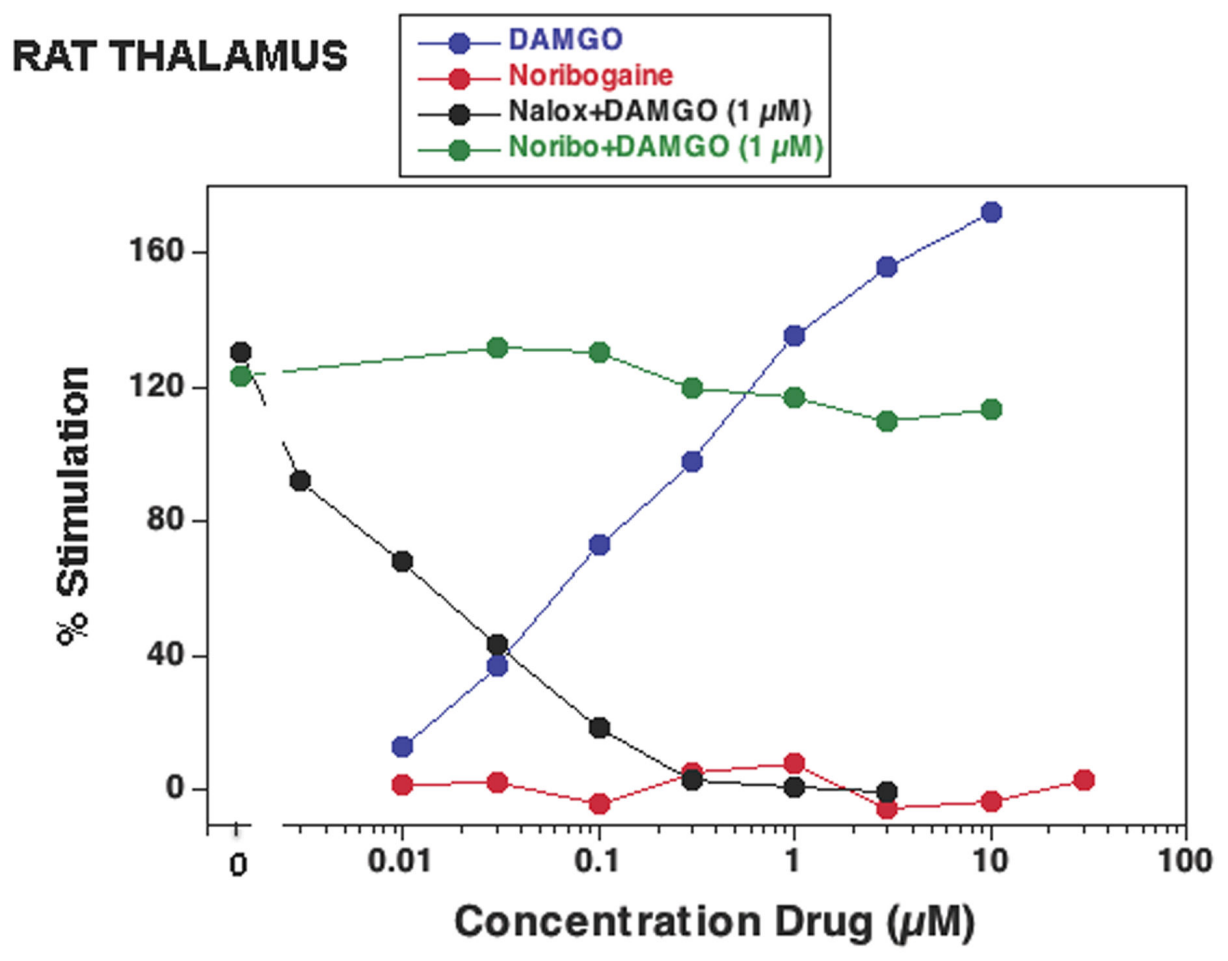
Effect on [^35^S]GTPγS binding of noribogaine (Noribo) by itself and in combination with DAMGO (1 µM) in Sprague-Dawley rat thalamic membranes (Childers lab). Tissue suspension aliquots were incubated with the indicated drug and 0.05 nM of [^35^S]GTPγS for 2 h at 30°C. DAMGO and naloxone (Nalox) were used as controls. Data are expressed as % of baseline and are from a representative experiment performed three times.

In thalamic membranes from Sprague-Dawley rats, DAMGO showed significant stimulation of [^35^S]GTPγS binding, with a maximal level of 214 ± 6% over basal, and an EC_50_ value of 238 ± 24 nM. In contrast, no significant stimulation was observed with concentrations up to up to 100 µM of ibogaine, noribogaine, or 18-MC (Figures 5, 6 and 7; Table 1). It is important to point out that the concentrations of noribogaine of 100 µM (Figures 6 and 7, Reith lab) or 30 µM (Figure 8, Childers lab) used in these separate experiments are far in excess of the EC_50_ of 324 nM reported previously by Pablo and Mash [43] for noribogaine in stimulating [^35^S]GTPγS binding in rat thalamic membranes. Concentrations of noribogaine that had no effect in this present study maximally stimulated [^35^S]GTPγS binding in rat thalamic membranes to a level observed with 10 µM DAMGO in the Pablo and Mash study. In a similar comparison regarding ibogaine, that study reported an approximate 20% stimulation by 100 µM ibogaine, whereas the results of this study indicated no stimulation (Figures 6 and 7; Table 1) (see below and Discussion). 

To confirm that MOR agonists of low G-protein stimulatory activity can be detected in Sprague-Dawley rat thalamic membranes, the partial agonist buprenorphine had a stimulatory effect with maximal efficacy of 14 ± 2% compared to DAMGO, and an EC_50_ value of 0.16 ± 0.02 nM (Table 1; Figure 6). The maximal efficacy of buprenorphine in thalamic membranes (14%) was much lower than that observed in transfected cells (61%) (Figures 2 and 6; Table 1). In comparing maximal stimulation of µ partial agonists in brain tissue vs. cells that express MORs at higher density, it is common to see relatively lower levels of maximal stimulation in brain tissue receptors [51,52]. Similarly, noribogaine and 18-MC, which were low efficacy partial agonists in cells (Figure 2: table 1), showed no efficacy in thalamic membranes of Sprague-Dawley rats (Figures 5, 6, 7 and 8; Table 1). Ibogaine at 10 and 100 µM reduced thalamic [^35^S]GTPγS binding below baseline (Figures 5 and 7); this phenomenon was not observed in cells and no further experiments were performed to provide evidence for potential inverse agonist action by ibogaine. 

Due to the discrepancy regarding the finding of ibogaine as a partial agonist and noribogaine as a full agonist published by Pablo and Mash [43] and the lack of agonist activity of either compound in thalamic membranes found in the present study as noted above, two separate sets of experiments were independently performed. In one set of experiments in the Janowsky lab utilizing thalamic membranes of Sprague Dawley and Fischer F344 rats, ibogaine, noribogaine, and 18-MC tested at concentrations up to 10^-5^ M did not stimulate [^35^S]GTPγS binding either with the procedures for tissue preparation and binding assay used in previous work in the Janowsky lab [49], or utilizing the procedures as specifically detailed by Pablo and Mash [43] (see Methods). However, DAMGO tested with the procedures used in previous work in the Janowsky lab [49], caused a robust increase in [^35^S]GTPγS binding with a EC_50_ value of 122 nM (Table 1). In another series of experiments in the Childers lab on thalamic membranes of Sprague-Dawley rats, DAMGO similarly stimulated [^35^S]GTPγS binding with an EC_50_ of 210 ± 33 nM, but noribogaine by itself at concentrations up to 30 µM did not have any effect (Figure 8; Table 1). 

In this study the Reith, Janowsky and Childers labs found values for EC_50_ for DAMGO in the [^35^S]GTPγS binding assay in membranes from rat thalamus of 238 nM, 122 nM, and 210 nM respectively (Table 1). These values contrast with the substantially lower value of 7.1 nM for EC_50_ of DAMGO in rat thalamic membranes reported in the Pablo and Mash study [43], which appears to be one to two orders of magnitude lower than values reported elsewhere in the literature for EC50 values for DAMGO stimulation of [^35^S]GTPγS binding in membranes from animal neural tissue. In addition to the good agreement among the collaborators in the present study, the results reported here replicate prior work in the Childers lab [52,53], and agree with two other independent labs that reported values for DAMGO EC_50_ in membranes from rat thalamus [54,55]. The agreement is not limited specifically to membranes from rat thalamus but extends generally to studies that utilized membranes from other neural tissues in the rat including striatum, hypothalamus and spinal cord [56,57], and other species including mouse [58,59], dog [60], and primate [61], all of which report EC_50_ values DAMGO in the [^35^S]GTPγS binding assay above 100 nM, in the range of 100 nM to 1200 nM. 

### Antagonism of MOR agonist effects by ibogaine, noribogaine, and 18-MC in rat thalamic membranes

As observed in cells, in Sprague-Dawley rat thalamic membranes ibogaine, noribogaine, and 18-MC inhibited the stimulatory response evoked by DAMGO (Figure 7). The stimulatory response evoked by 1 µM DAMGO (~ 83% of the maximal response evoked by 10 µM DAMGO) was inhibited by ibogaine, noribogaine, and 18-MC with an IC_50_ of 19.3 ± 4.3 µM, 84.0 ± 27.8 µM, and 83.3 ± 19.4 µM (i.e., functional K_i_ values of 3.05 µM, 13.3 µM, and 13.2 µM) respectively (Figure 7; table 1). In a separate set of experiments in the Childers lab, noribogaine was tested for antagonist effects. At concentrations up to 10 µM it did not inhibit the stimulation evoked by 1 µM DAMGO in thalamic membranes from Sprague-Dawley rats, in contrast to the decrease seen with naloxone (Figure 8; Table 1). Both Figures 7 and 8 indicate a similar lack of effect of 10 µM noribogaine when co-present with 1 µM DAMGO. The concentration of 10 µM of noribogaine is much lower than the IC_50_ of 84 µM that was observed in Sprague-Dawley rat thalamic membranes (Figure 7). Thus, the data from both the Reith and Childers labs indicate that noribogaine’s weak antagonist action against 1 µM DAMGO is apparent only at concentrations of noribogaine exceeding 10 µM.

### Evaluation utilizing autoradiography of activation of G-proteins by noribogaine in rat brain sections

**Figure 9:**
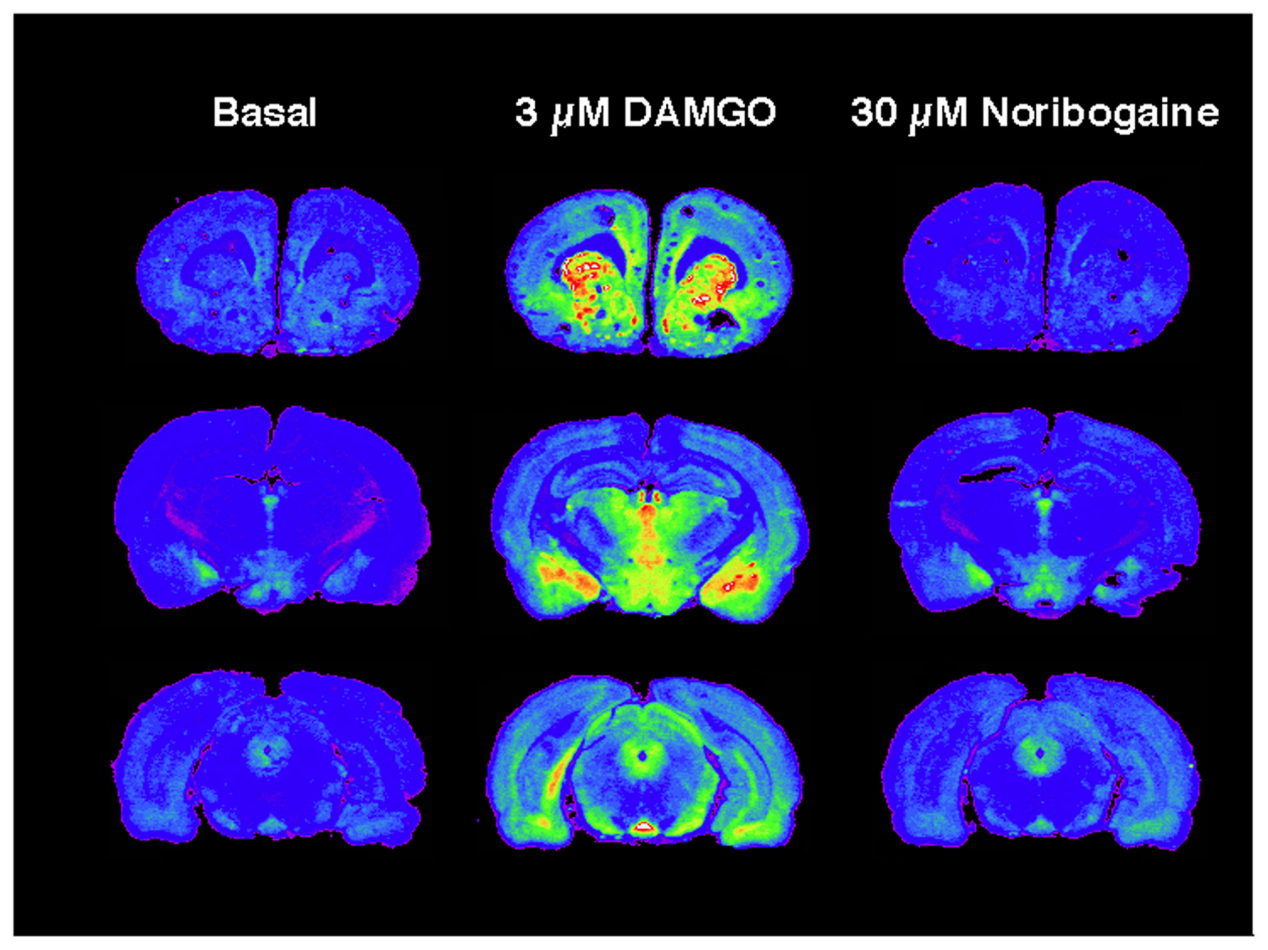
Effect of noribogaine (30 µM) compared with DAMGO (3 µM) on [^35^S]GTPγS binding measured by autoradiography in brain slices from Sprague-Dawley rats (Childers lab). Coronal sections were incubated with 0.04 nM of [^35^S]GTPγS for 2 h at 30°C with or without 3 µM DAMGO or 30 µM noribogaine. Basal binding was deducted to obtain net agonist-stimulated binding. Results shown are from a representative experiment, carried out three times in sections from three individual rats.

Although noribogaine had no effect on G-protein stimulation in membranes from rat thalamus, a brain region with high levels of MOR, it is possible that effects of noribogaine might be observed in other brain regions. To examine this possibility, activation of G-proteins by noribogaine was examined in brain sections using autoradiography (Childers lab). Figure 9 shows typical autoradiograms from three levels of Sprague-Dawley rat brain, showing high levels of MOR-stimulated [^35^S]GTPγS binding with 3 µM DAMGO in caudate, nucleus accumbens, and cingulate cortex (top level); thalamus, amygdala and hypothalamus (middle level); superior colliculus, periaqueductal grey and entopeduncular nucleus (bottom level). In contrast to the stimulation of [^35^S]GTPγS binding by DAMGO, noribogaine by itself at concentrations up to 30 µM had no effect on [^35^S]GTPγS binding in any brain region.

## Discussion

Ibogaine, noribogaine and 18-MC were MOR antagonists with functional Ke values ranging from 3 uM (ibogaine) to 13 uM (noribogaine and 18MC) in rat thalamic membranes. Both noribogaine and 18-MC were inactive, or weak partial agonists respectively in CHO rMOR and HEK mMOR cells. Ibogaine did not stimulate [^35^S]GTPγS binding in any MOR expressing cells. Ibogaine and noribogaine were antagonists in HEK mMOR cells and CHO hMOR cells respectively. Noribogaine did not stimulate [^35^S]GTPγS binding measured by autoradiography in rat brain slices. The relative lack of functional activation of the MOR by ibogaine, noribogaine, and 18-MC observed in this study suggests that the effects of these iboga alkaloids on opioid withdrawal are not mediated by an MOR agonist action. 

While the functional effects of ibogaine in humans and animal models appear to indicate that it is not acting as an orthosteric MOR agonist, the potentiation of morphine analgesia by ibogaine, without an analgesic effect by ibogaine itself [23,30-35] leaves open the possibility that it could be an allosteric MOR agonist. Here the use of the of the [^35^S]GTPγS binding assay indicates that ibogaine is not an allosteric MOR agonist, because it behaved as an antagonist in the presence of morphine and DAMGO. The present data do not rule out the possibility that ibogaine at concentrations lower than those displaying antagonist effects could enhance the stimulation of [^35^S]GTPγS binding by low concentrations of agonist, as this particular combination was not tested in this work. However, this seems unlikely because opioid detoxification, the indication for which ibogaine is most commonly used [4-6], is unlikely to be mediated by an allosteric agonist action because ibogaine itself is not an orthosteric agonist and detoxification does not require opioid co-administration. Furthermore, ibogaine's lack of primary analgesic effect in vivo, and its not producing opioid overdose in nontolerant individuals argue against the idea that ibogaine is acting as an allosteric MOR agonist with regard to potentiation of endogenous opioids. 

The iboga alkaloid mechanism of action appears to differ from non-opioid agents known to have clinical effects on opioid tolerance and withdrawal, including antagonists of N-Methyl-D-aspartate-type glutamate (NMDA) receptor or α_2_ adrenergic receptor agonists. Ibogaine is an NMDA receptor antagonist [62], and NMDA antagonists such as memantine diminish signs of opioid withdrawal in preclinical models [63] and humans [64]. However, the example of 18-MC suggests that NMDA antagonism is not critical to the iboga alkaloid mechanism of action. 18-MC is a product of rational pharmaceutical design with the aim of developing a safer congener that differs from ibogaine at three of the 21 positions on the ibogamine skeleton [65] (Figure 1). Although 18-MC lacks significant affinity for the NMDA receptor [26], it is equally effective as ibogaine in the animal model of withdrawal. A mechanism of action involving agonist action at α_2_ adrenergic or imidazoline receptors similar to imidazoline α_2_ agonists such as clonidine also seems unlikely, because ibogaine has no significant affinity for the α_2_ receptor [28,29]. 

Ibogaine and 18-MC are antagonists of the α3β4 nicotinic acetylcholine receptor (nAChR). Antagonism of the α3β4 nAChR is a leading theory regarding the mechanism of action of the effect of iboga alkaloids on drug self-administration [66-70], but does not appear to readily explain the effect of iboga alkaloids in acute opioid detoxification, or prolonged effects that appear to persist beyond pharmacokinetic elimination [71]. In one study combinations of low doses of drugs with a common action of inhibition of the α3β4 nAChR were given to rats utilizing the naltrexone-precipitated withdrawal paradigm, with the intention of producing additive effects at the α3β4 nAChR while minimizing effects mediated by their actions at other receptors [67]. Only two signs of withdrawal, diarrhea and weight loss were reduced, which could be attributed to actions at peripheral receptor sites, and the overall effect on withdrawal signs was small relative to that of 18-MC administered centrally or systemically. 

It appears unlikely that a laboratory impurity in the iboga alkaloids utilized in this study could account for the results. These iboga alkaloid compounds, which produced similar effects on [^35^S]GTPγS binding are the product of distinct manufacturing processes. Ibogaine was extracted from *T. iboga* root bark and noribogaine produced by demethylation of ibogaine, whereas 18-MC in contrast is a product of a total synthesis. Please see Supporting Information Files S1 and S2 for a more extensive discussion along with the analyses of the samples used in this study. 

The results reported here differ from a prior study in which noribogaine was reported to be a full MOR agonist in thalamic membranes [43]. In the present study, noribogaine was only a partial agonist in HEK cells but did not stimulate [^35^S]GTPγS binding in CHO cells with overexpressed MOR. Three separate labs in this study found that noribogaine did not stimulate [^35^S]GTPγS binding in thalamic membranes. This included a set of experiments in the Janowsky lab in which noribogaine did not stimulate [^35^S]GTPγS binding in thalamic membranes either with the procedures for tissue preparation and binding assay used in previous work in the Janowsky lab [49], or following the procedures as specifically detailed in the prior study in which noribogaine was reported to be a full MOR agonist [43]. Additionally in thalamic membranes noribogaine was not an agonist, but an antagonist versus DAMGO as an agonist. Autoradiography also indicated a lack of an effect of noribogaine on [^35^S]GTPγS binding in brain slices from other rat brain regions. 

The timing of the onset of the effect of ibogaine in opioid withdrawal relative to its conversion to noribogaine may additionally be inconsistent with a hypothesis that a putative MOR agonist action of noribogaine mediates the effect of ibogaine in opioid detoxification. In the naltrexone-precipitated withdrawal paradigm, ibogaine has been given intraperitoneally (i.p.) 30 minutes prior to the administration of naltrexone [72], an interval following i.p. administration at which ibogaine absorption is maximal (S.D. Glick personal communication), and a time at which most ibogaine may not yet have been transformed to noribogaine in view of the estimated half-life of ibogaine in the rat of 1 to 3 hours [73-75]. Similarly, ibogaine treatment providers observe improvement in opioid withdrawal symptoms in humans within an hour following ingestion [4,5,27], apparently coinciding with the onset of oral absorption and preceding most of the conversion of ibogaine, which has an estimated half-life of 4 to 7 hours in humans [6,11]. Additionally, 18-MC, which was without an MOR agonist effect, is equally efficacious as ibogaine in the rat in the naloxone-precipitated withdrawal paradigm [12,13]. Although noribogaine has been hypothesized to be involved in effects of ibogaine on self-administration [74,76,77], this present study suggests that this would not be mediated by an MOR agonist action. 

If the in vivo effects of ibogaine are not explained by direct interaction with MORs, what entities and mechanisms are involved? Perhaps the target of ibogaine is better understood as a structural motif or homology rather than a single identified receptor protein. The mechanism of action of iboga alkaloids in addiction appears unexplained on the basis of orthosteric binding to known receptors, channels or transporters. An unknown target protein appears to be involved, but it is possible that it carries a motif that is shared with other known targets of iboga alkaloids. Structural biological investigations of iboga alkaloids have mainly involved two proteins, the serotonin transporter (SERT) and the nAChR, both of which ibogaine inhibits allosterically [78,79]. Future crystallographic investigation of the binding sites of iboga alkaloids in the LeuT, a bacterial homolog of the SERT [80], *Torpedo* nAChR [81], adenylate cyclases (ACs) [82], or other target proteins will help elucidate a possible structural motif for iboga alkaloids.

It is possible that the target for iboga alkaloids resides in pathways linked to the MOR, downstream from the action of opioid agonists at the MOR itself, or even downstream from an agonist action at non-opioid receptors. The negative coupling of AC to the MOR, and the upregulation of AC in opioid withdrawal are cardinal MOR-related signaling events [83-85]. Rabin and Winter [39] reported that ibogaine and noribogaine potentiate the inhibition of AC by morphine, and also by serotonin, while having no effect on AC when given alone. This suggests that iboga alkaloids can act downstream from receptor-coupled G protein activation, possibly directly at AC, mediating effects on opioid withdrawal that are unexplained by MOR-coupled G protein activation [27,39]. The speculation that ibogaine modulates AC directly fits in with the current perception of AC as a signal transduction machine that has orthosteric and allosteric ligands [82]. Within the orthosteric catalytic site, ACs carries allosteric sites for compounds such as P-site inhibitors [86], and by analogy it might be considered that the allosteric region interacting with Gi protein (from MOR activation) carries an additional allosteric interaction site for compounds such as ibogaine. Clearly, as this is downstream from MOR activation with the exchange of GTP for GDP, the [^35^S]GTPγS assay in the present experiments cannot detect such a phenomenon. 

Some in vivo evidence appears consistent with the hypothesis that iboga alkaloids allosterically inhibit AC. Ibogaine and noribogaine potentiate morphine analgesia, but do not produce analgesia when administered alone [23,30-35], which is also consistent with an effect of inhibition of AC in view of the upregulation of AC in pain sensitization associated with opioid withdrawal [87] and analgesic effects of drugs targeting the inhibition of AC [88,89]. Allosteric inhibition of AC could also explain why iboga alkaloids administered alone do not produce signs of opioid overdose, but potentiate the toxicity of opioids [30,36,73,90,91], and why iboga alkaloids diminish, rather than exacerbate withdrawal signs in opioid dependence [4-6,12-25] despite the present finding of an MOR antagonist action. It is also questionable whether the concentrations at which antagonism occurs are pharmacologically relevant; the salient finding to be reported here may be simply that the [^35^S]GTPγS assay results indicate that these compounds are not MOR agonists. 

In many of the above observations, there is opioid tolerance by pretreatment or prior exposure to opioid agonists. Other findings also support an apparent selectivity of functional effects of iboga alkaloids on neuroadaptations associated with opioid tolerance. Ibogaine and noribogaine diminish tolerance in morphine-tolerant mice [33,35,36], and dose-dependently potentiate the antinociceptive effect of morphine in morphine-tolerant but not in morphine-naïve mice [35]. Ibogaine has relatively selective effects on dopamine efflux in the nucleus accumbens [92] and locomotor activity [93,94] in morphine-tolerant versus non-tolerant rats. Ibogaine’s clinical effect of opioid detoxification without causing opioid overdose in non-tolerant individuals also suggests selectivity for neuroadaptations associated with prior exposure. 

 In conclusion, we report that Ibogaine, noribogaine, and 18-MC were weak antagonists without any agonist or partial agonist effects in rat thalamic membranes, and were either inactive or antagonists, or had very low efficacy as partial agonists in cells overexpressing the MOR. These results appear to indicate that an MOR agonist effect does not account for the mechanism of action of iboga alkaloids on opioid withdrawal. Taken together with existing evidence that their mechanism of action also differs from that of other non-opioids with clinical effects on opioid tolerance and withdrawal, these findings suggest a novel mechanism of action, and support the search for the targets of iboga alkaloids as a potentially interesting approach to drug discovery and neurobiological investigation.

## Supporting Information

File S1 **Supporting GC-MS and HPLC-MS graphs and NMR spectral data for Ibogaine sources A and B, noribogaine sources C and D, and 18-MC.** The respective graphs/analytic data for each compound are presented following order: ibogaine source A, ibogaine source B, noribogaine source C, noribogaine source D, and 18-MC. Within this Supporting Information File, pages are labeled and numbered separately for each compound. On each page, the respective iboga alkaloid and source are indicated as headers at top center and the page numbers appear on the right upper corner.
(PDF)

File S2 **Supporting text to accompany File S1.** The supporting text references the graphs/analytic data for each compound and source according to headers and page numbers indicated above in File S1.
(PDF)
