# Supplementary material for: Effect of Iboga Alkaloids on µ-Opioid Receptor-Coupled G Protein Activation
Source: PLoS One. 2013 Oct 16;8(10):e77262. doi: 10.1371/journal.pone.0077262 (PMC3818563; doi:10.1371/journal.pone.0077262)
Supplement: File S1 — Supporting GC-MS and HPLC-MS graphs and NMR spectral data for Ibogaine sources A and B, noribogaine sources C and D, and 18-MC. The respective graphs/analytic data for each compound are presented following order: ibogaine source A, ibogaine source B, noribogaine source C, noribogaine source D, and 18-MC. Within this Supporting Information File, pages are labeled and numbered separately for each compound. On each page, the respective iboga alkaloid and source are indicated as headers at top center and the page numbers appear on the right upper corner. (PDF) [file pone.0077262.s001.pdf]

MS Data Review Active Chromatogram and Spectrum Plots - 6/27/2013 2:28 PM

File: c:\varianws\data\martin kuehne\bor mk 5-13_5-13-2010_1.sms
Sample: BOR MK 5-13 Operator: Bruce
Scan Range: 1 - 1625 Time Range: 0.00 - 25.64 min. Date: 5/13/2010 1:59 PM

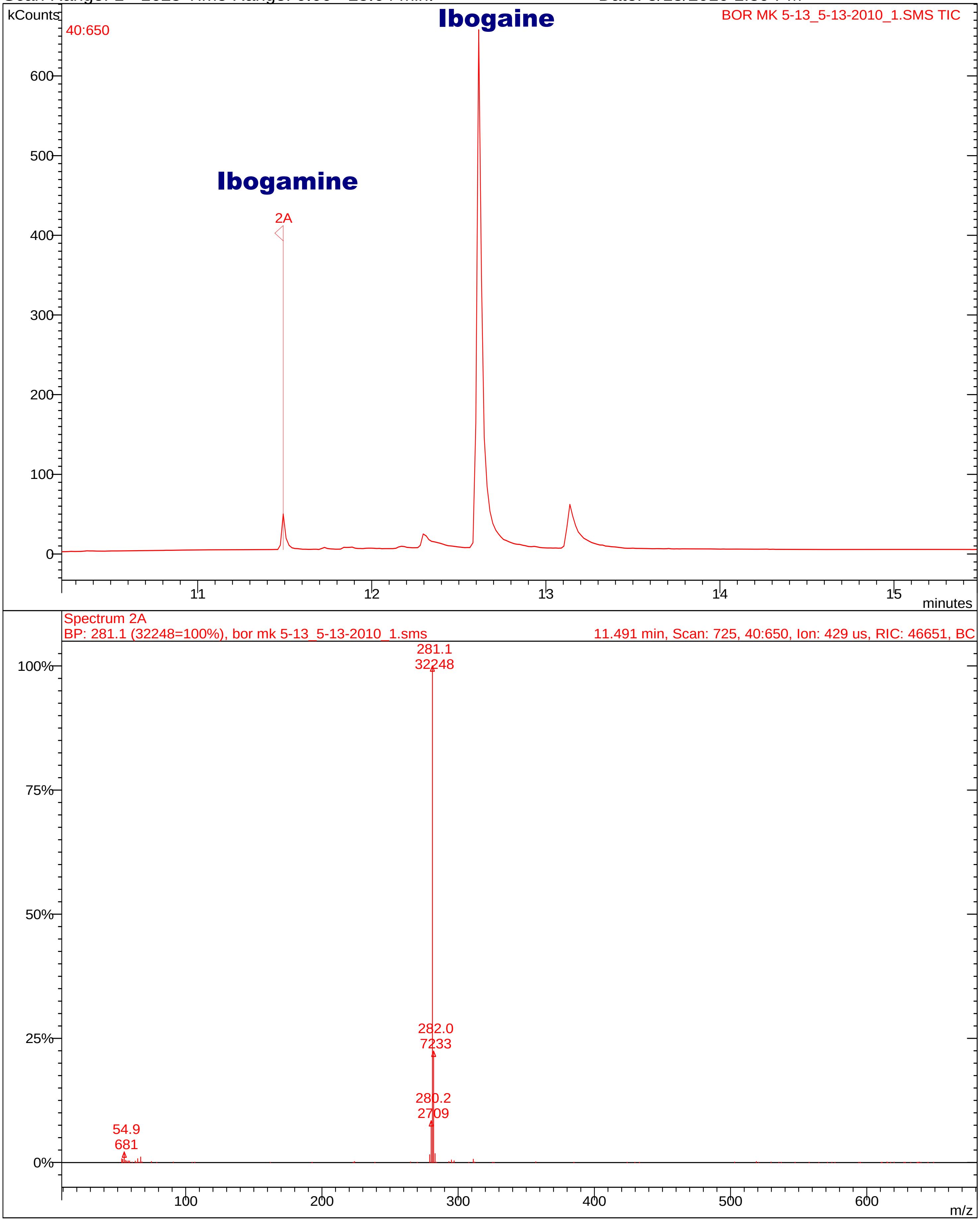

Ibogaine source A

MS Data Review All Plots - 6/27/2013 2:30 PM

File: c:\varianws\data\martin kuehne\bor mk 5-13_5-13-2010_1.sms
Sample: BOR MK 5-13 Operator: Bruce
Scan Range: 1 - 1625 Time Range: 0.00 - 25.64 min. Date: 5/13/2010 1:59 PM

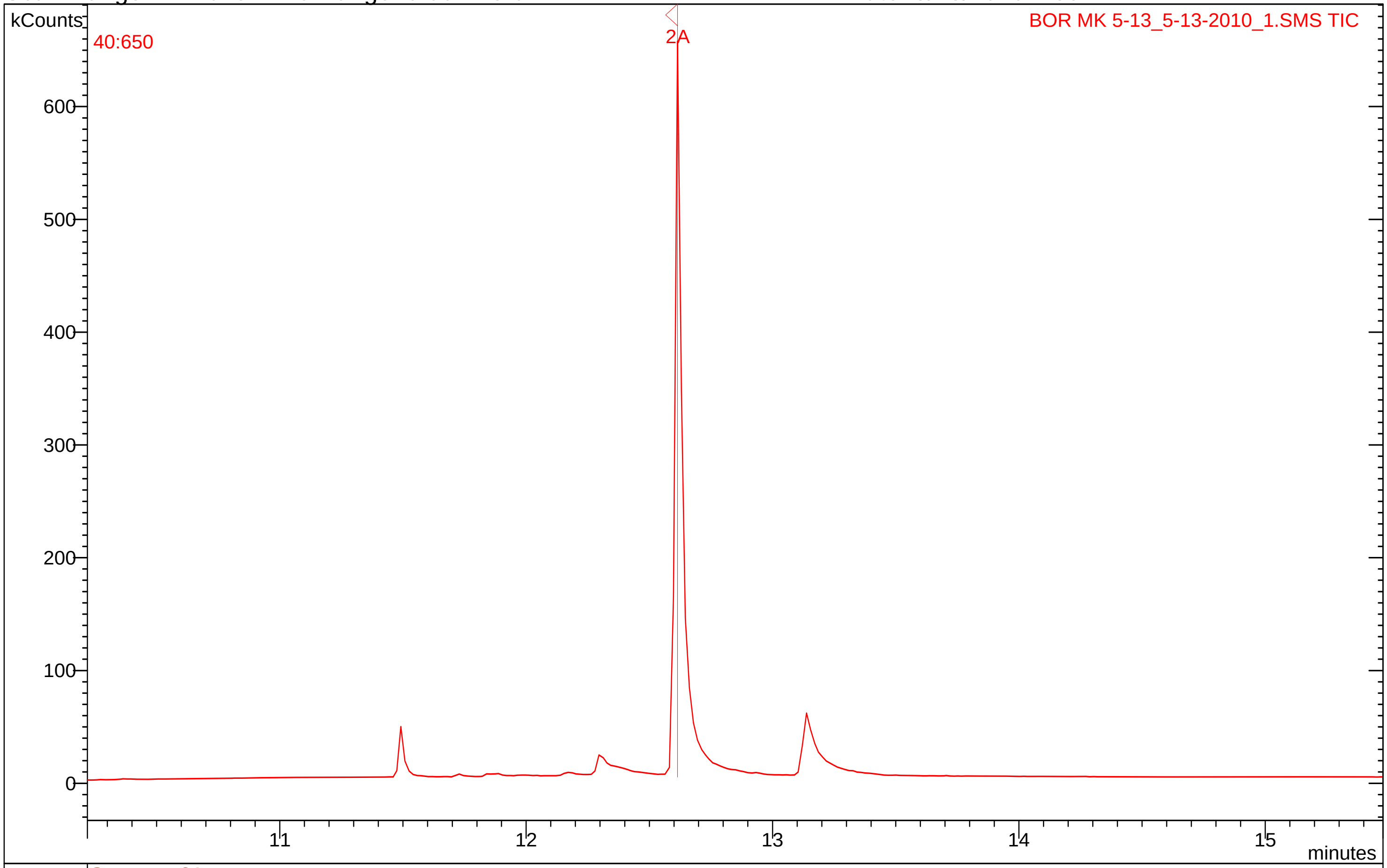

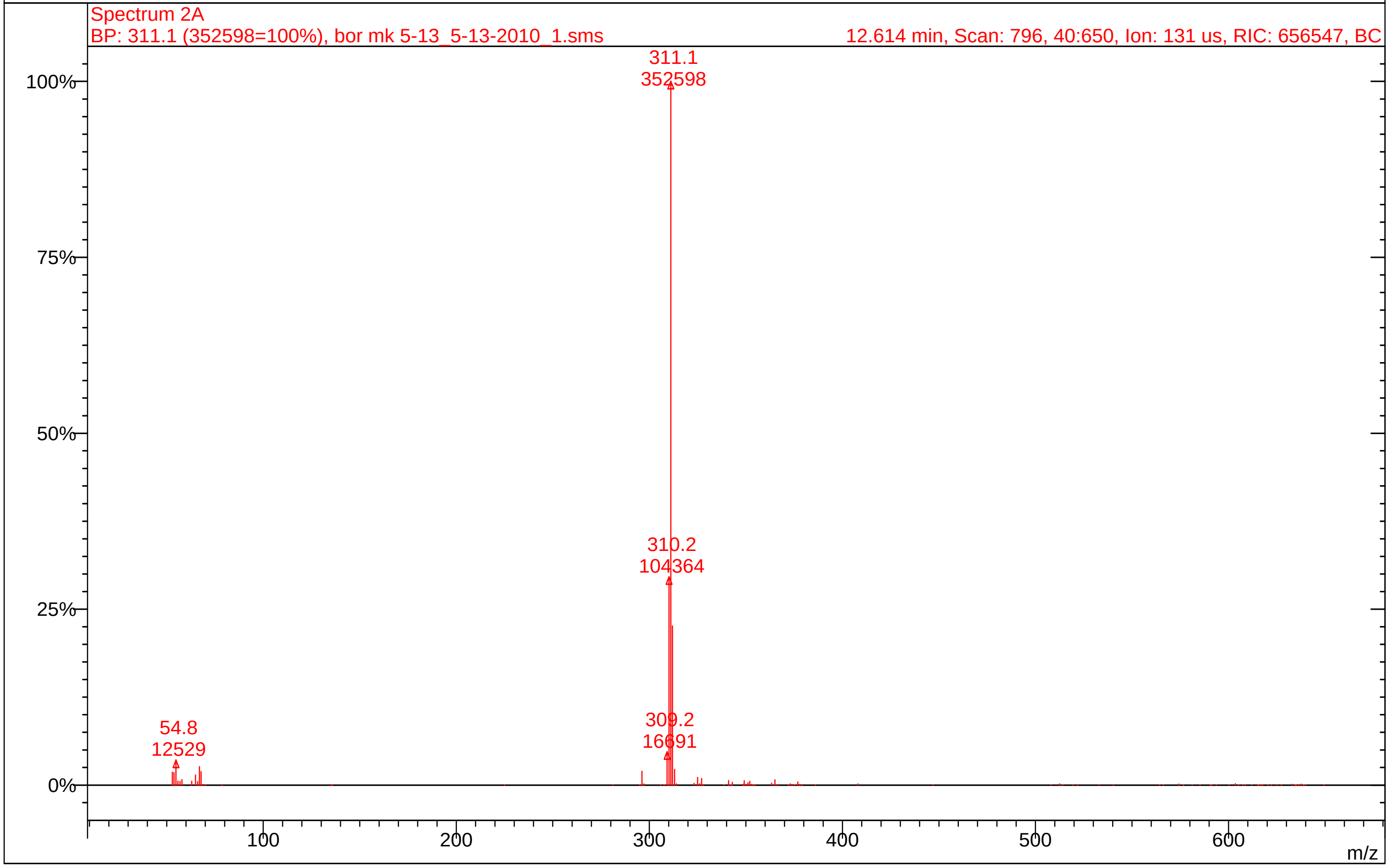

MS Data Review Active Chromatogram and Spectrum Plots - 6/27/2013 2:31 PM

File: c:\varianws\data\martin kuehne\bor mk 5-13_5-13-2010_1.sms
Sample: BOR MK 5-13 Operator: Bruce
Scan Range: 1 - 1625 Time Range: 0.00 - 25.64 min. Date: 5/13/2010 1:59 PM

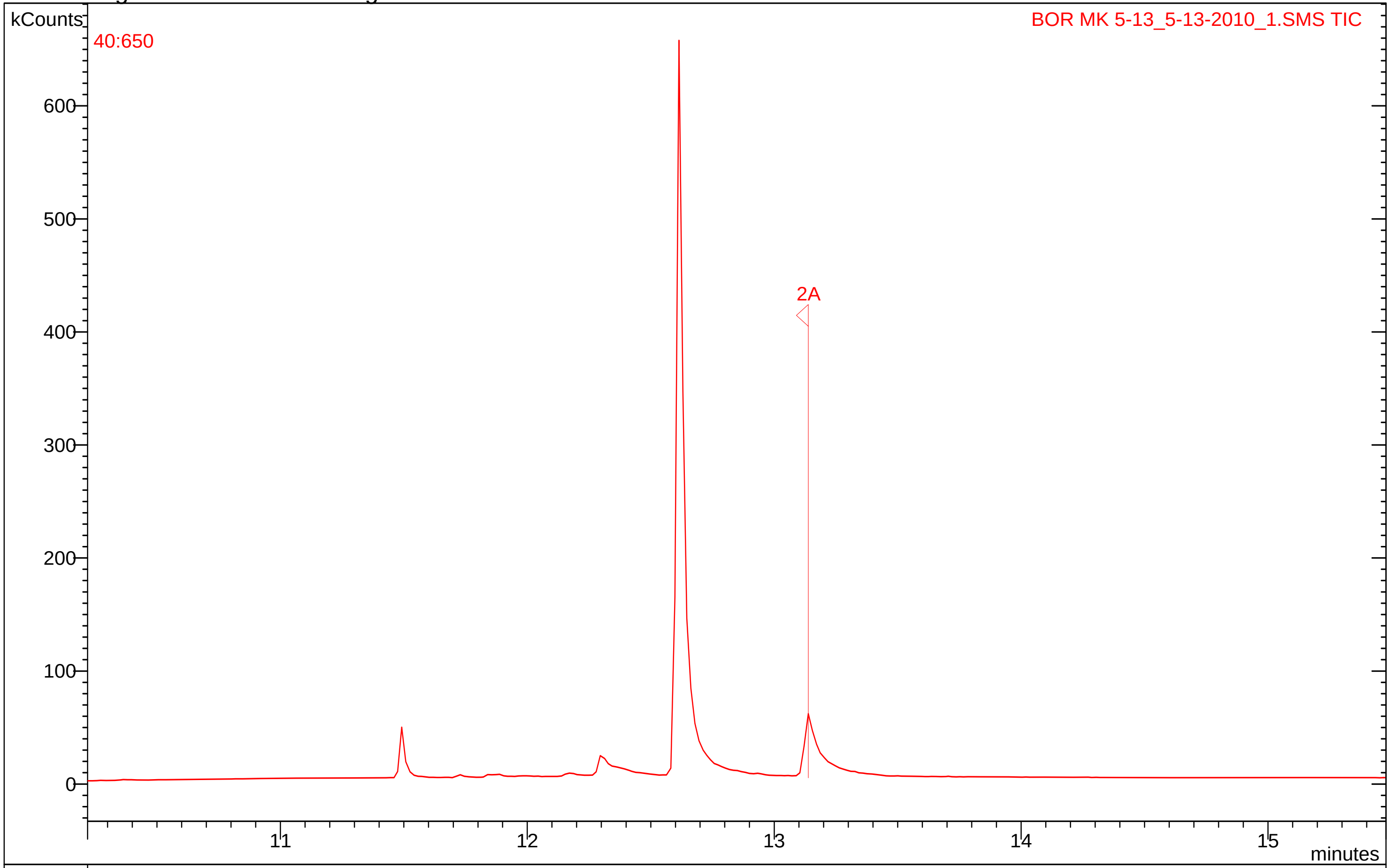

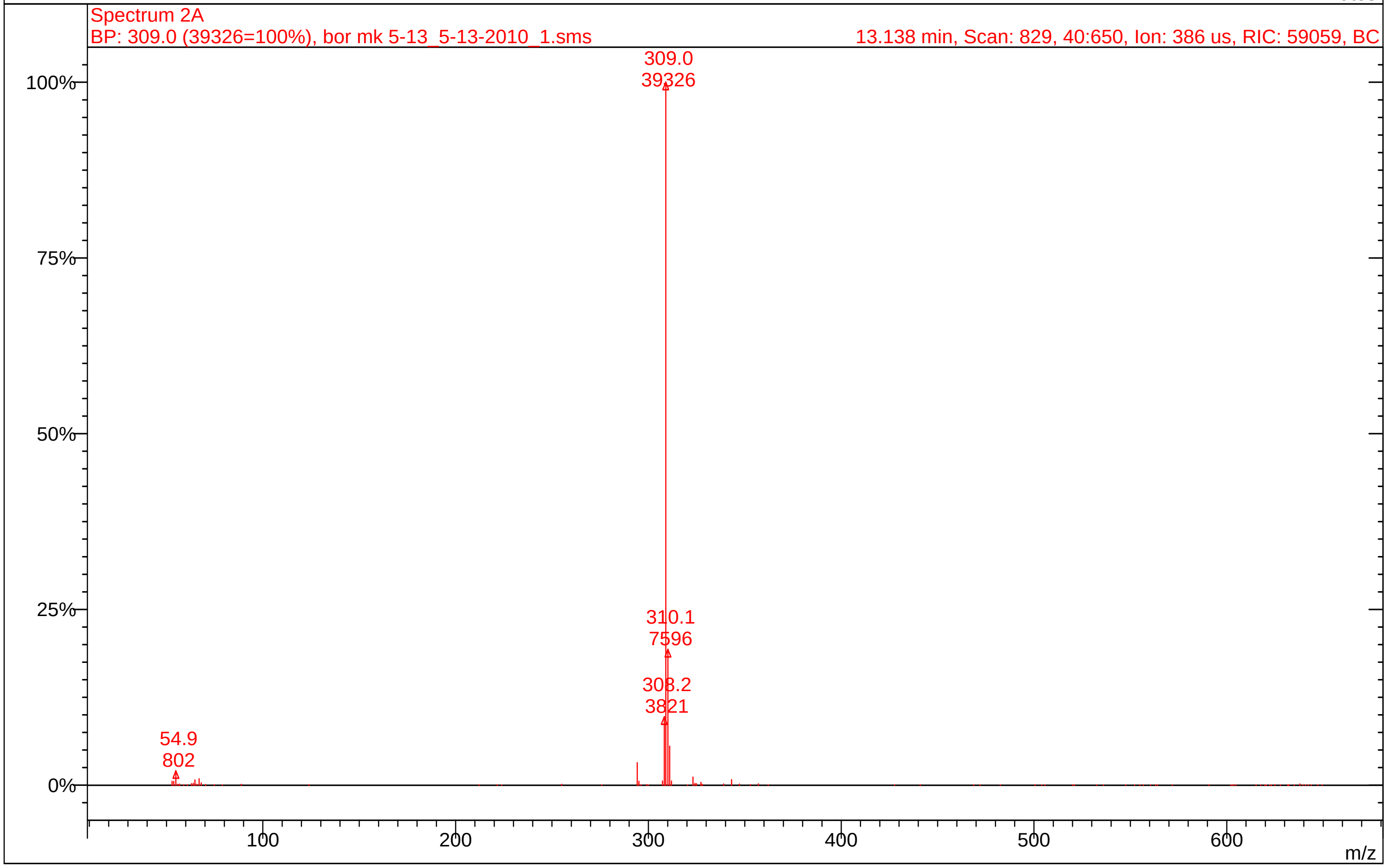

RTI INTERNATIONAL*

DATA SHEET

Ibogaine HCl

RTI Log Number: 3857-194 (Sigma Lot No. 32H0349)

**Chemical Abstracts Name**	12-Methoxyibogamine hydrochloride
**DEA Drug Code Number**	7260
**DEA Schedule**	1
**CAS Number**	36415-61-9
**INN Name**	None

Structure:

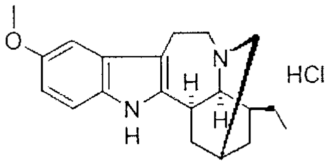

HCl

Molecular Formula: $C_{20}H_{26}N_2O{\cdot}HCl$

Molecular Weight: 346.9

Appearance: Beige crystalline powder

Analyses: Chromatographic Purity: 97.2 ± 0.06% pure by Reversed Phase HPLC (total area analysis)

NMR: The ^{1}H spectrum is consistent with the structure of Ibogaine HCl and identical to the respective spectrum provided by the supplier (performed in 2009).

Mass Spectrometry: Mass spectrum matches with reference library and conforms to known structure.

TLC: On Silica ($^{60}F_{254}$) using "Clarke's Isolation and Identification of Drugs" System TA (100 mL MeOH:1.5 mL ammonium hydroxide). Visualization by UV. Single spot Rf 0.50. No impurities detected.

Water: 1.03 ± 0.01% (Karl Fischer)

Melting Point: Mettler Toledo MP70 Melting Point System: darkened @ 256 °C
Merck Index, 14th Edition, 2006; Decomposes at 299 - 300 °C

Analyses Performed: February 2012 (unless noted otherwise).

Analyses Reference: 13406-198

Storage: Store in a cool, dark place. Protect from moisture.

Caution: **NOT FOR HUMAN USE.**

Please Note: Technical questions about this compound should be directed to:
Dr. Poonam Pande at (919) 541-7210.

Approved by: P Pande Date 07/05/12

==

SHIPMENT REFERENCE ONLY

Quantity: .02097g

Reference: SAF# 017618

RTI International is a trade name of Research Triangle Institute.

Ibogaine source B

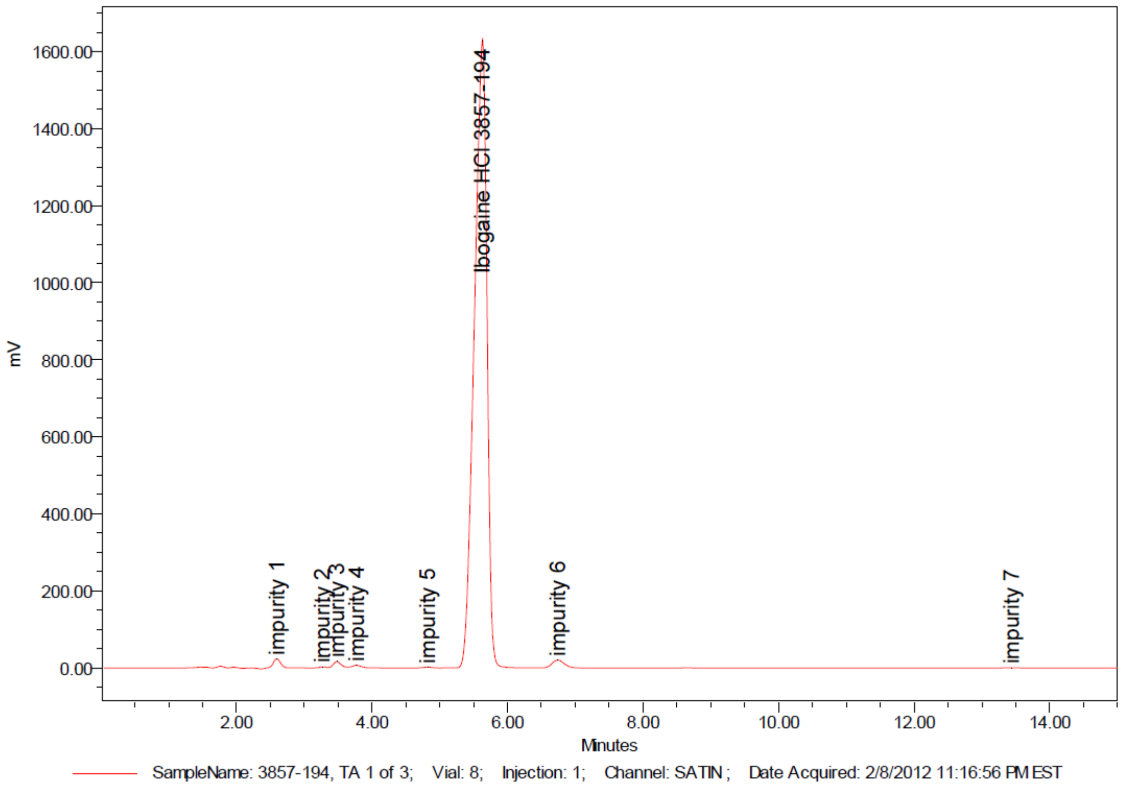

Ibogaine source B

File : C:\MSDChem\1\DATA\ANTIMONY\NTP\Seq-2012-02-17-131222338UTC\02
... 161203.D
Operator : Anderson O. Cox
Instrument : Antimony
Acquired : 17 Feb 2012 9:35 using AcqMethod 012512A.M
Sample Name: 3858-40-2 Ibogaine
Misc Info : 13406

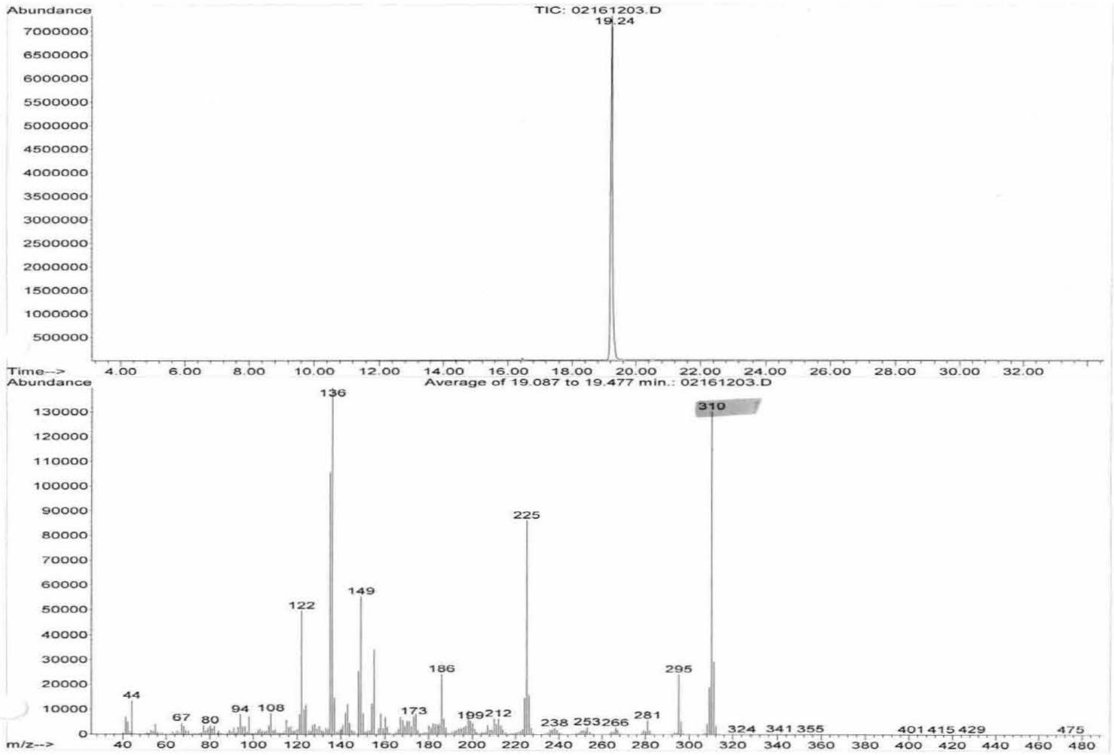

Noribogaine source C

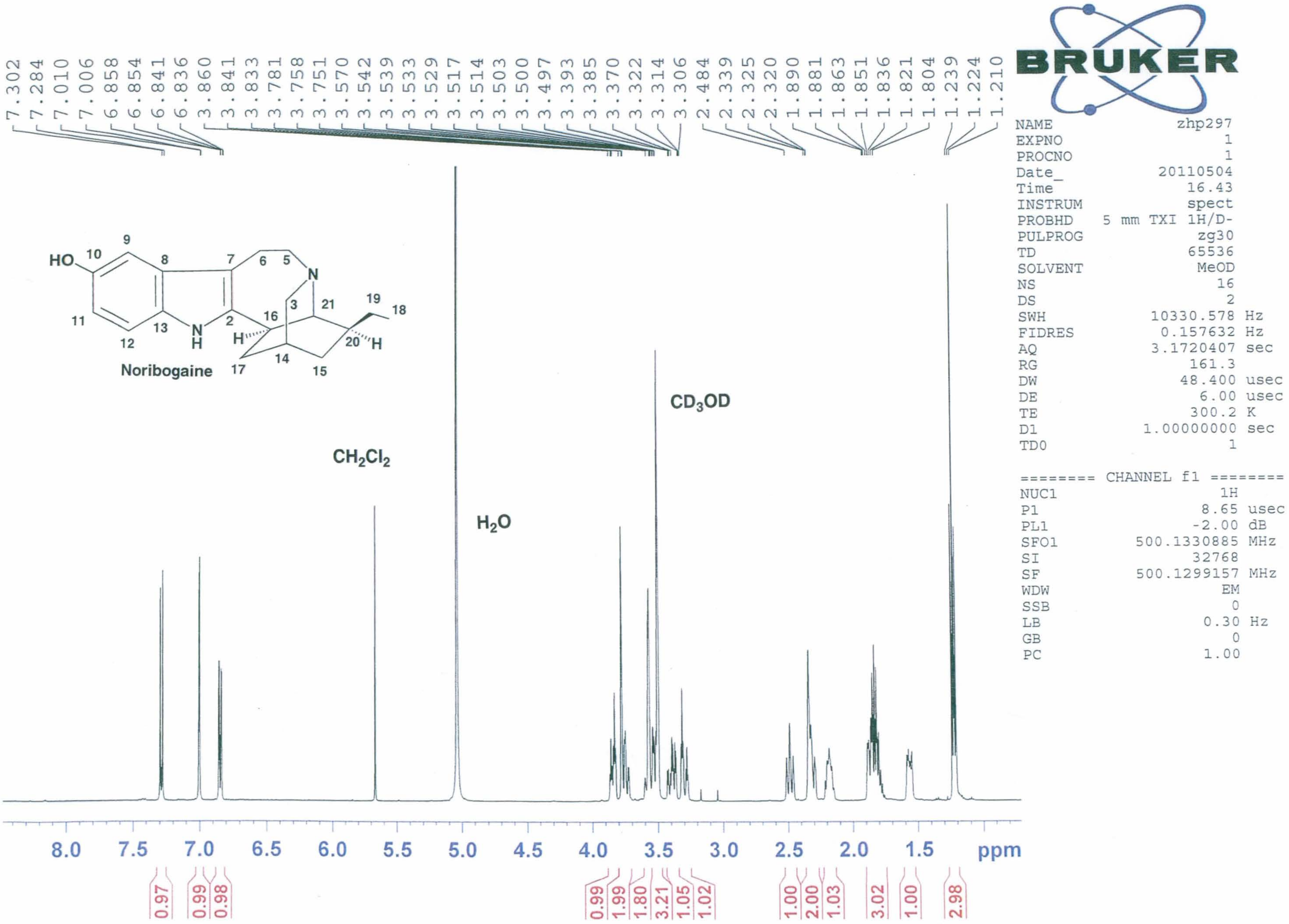

Noribogaine source C

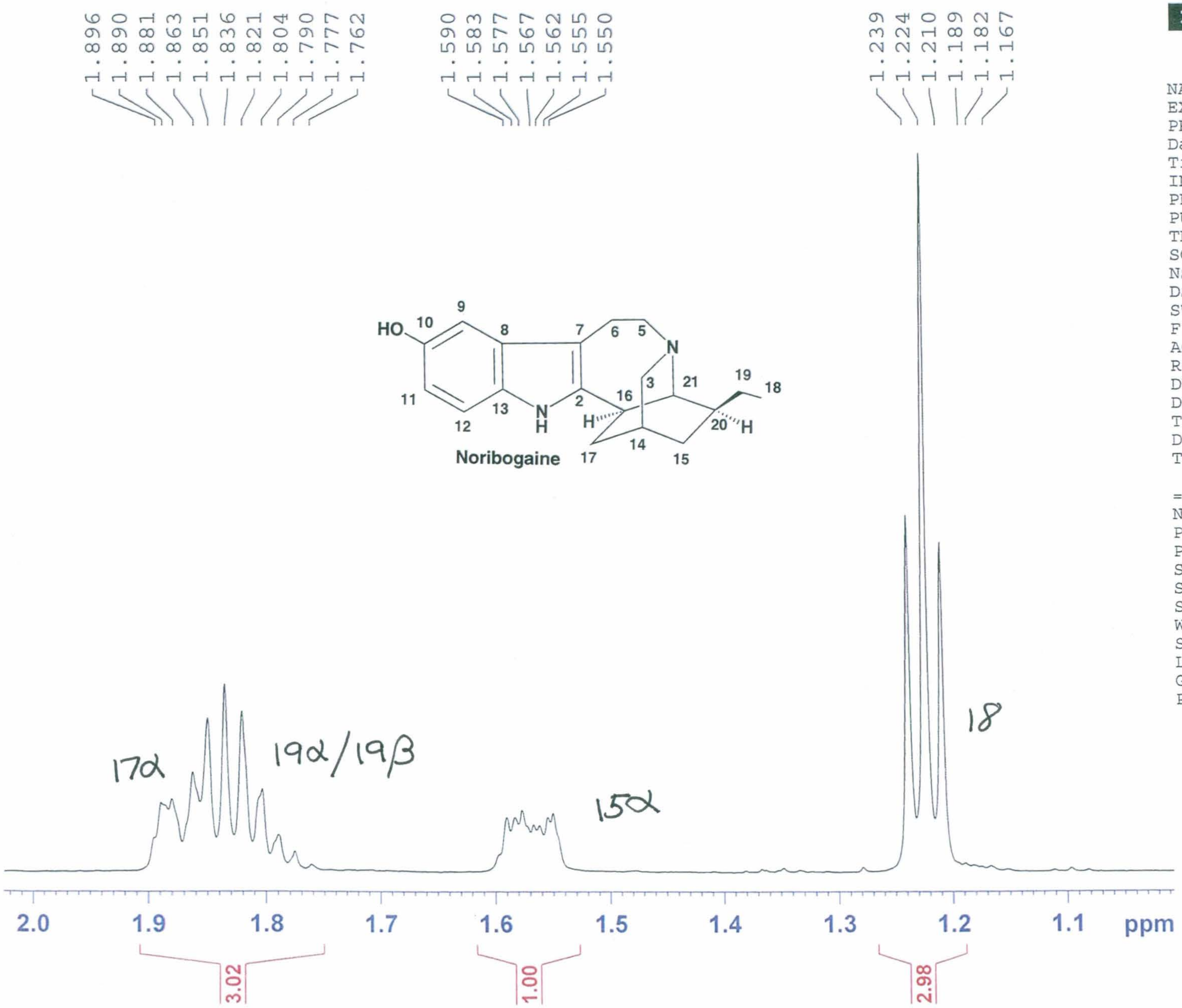

NAME	zhp297
EXPNO	1
PROCNO	1
Date_	20110504
Time	16.43
INSTRUM	spect
PROBHD	5 mm TXI 1H/D-
PULPROG	zg30
TD	65536
SOLVENT	MeOD
NS	16
DS	2
SWH	10330.578 Hz
FIDRES	0.157632 Hz
AQ	3.1720407 sec
RG	161.3
DW	48.400 usec
DE	6.00 usec
TE	300.2 K
D1	1.00000000 sec
TD0	1

======== CHANNEL f1 ========

NUC1	1H
P1	8.65 usec
PL1	-2.00 dB
SFO1	500.1330885 MHz
SI	32768
SF	500.1299157 MHz
WDW	EM
SSB	0
LB	0.30 Hz
GB	0
PC	1.00

Noribogaine source C

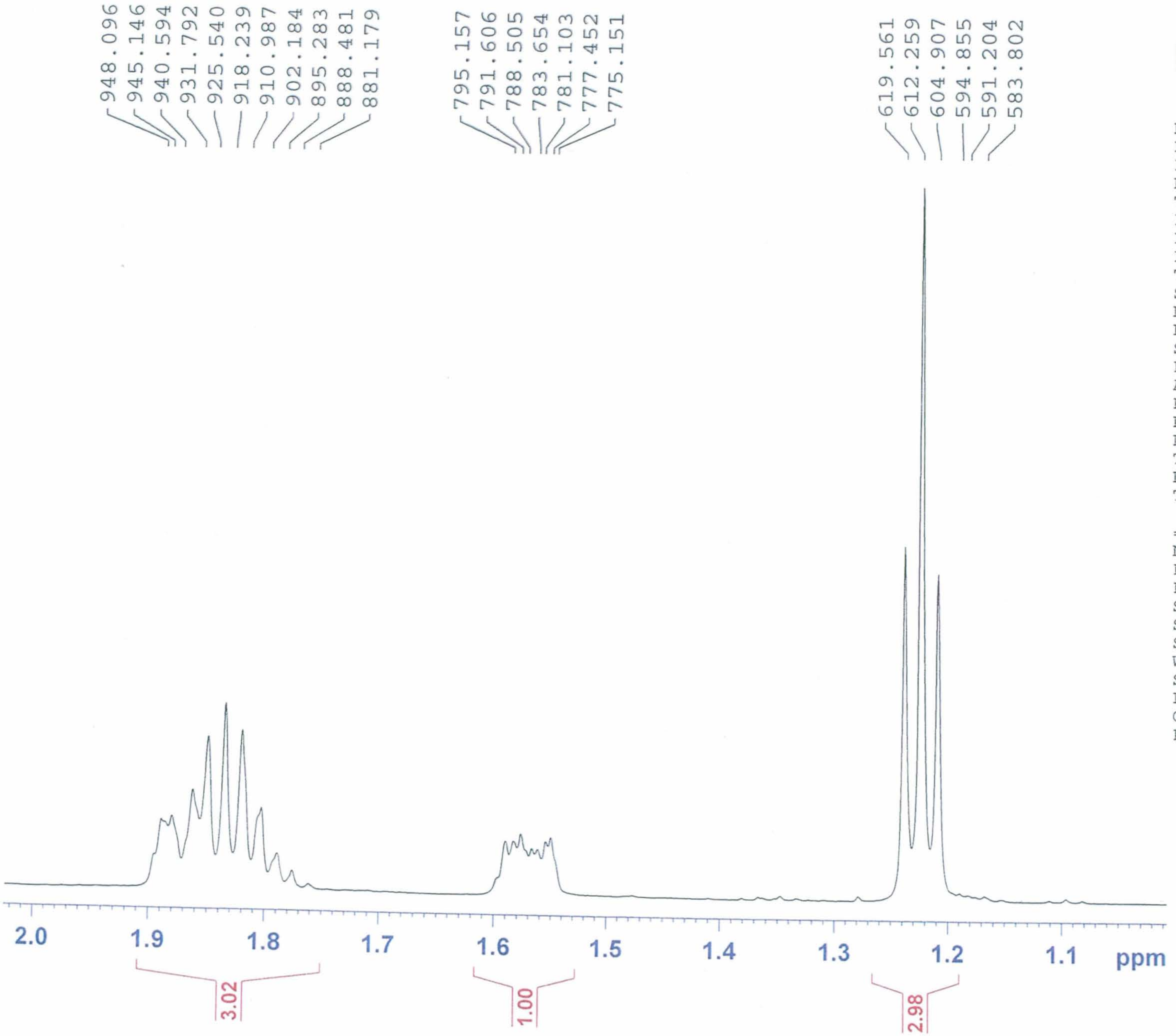

BRUKER

NAME	zhp297
EXPNO	1
PROCNO	1
Date_	20110504
Time	16.43
INSTRUM	spect
PROBHD	5 mm TXI 1H/D-
PULPROG	zg30
TD	65536
SOLVENT	MeOD
NS	16
DS	2
SWH	10330.578 Hz
FIDRES	0.157632 Hz
AQ	3.1720407 sec
RG	161.3
DW	48.400 usec
DE	6.00 usec
TE	300.2 K
D1	1.00000000 sec
TD0	1

======== CHANNEL f1 ========

NUC1	1H
P1	8.65 usec
PL1	-2.00 dB
SFO1	500.1330885 MHz
SI	32768
SF	500.1299157 MHz
WDW	EM
SSB	0
LB	0.30 Hz
GB	0
PC	1.00

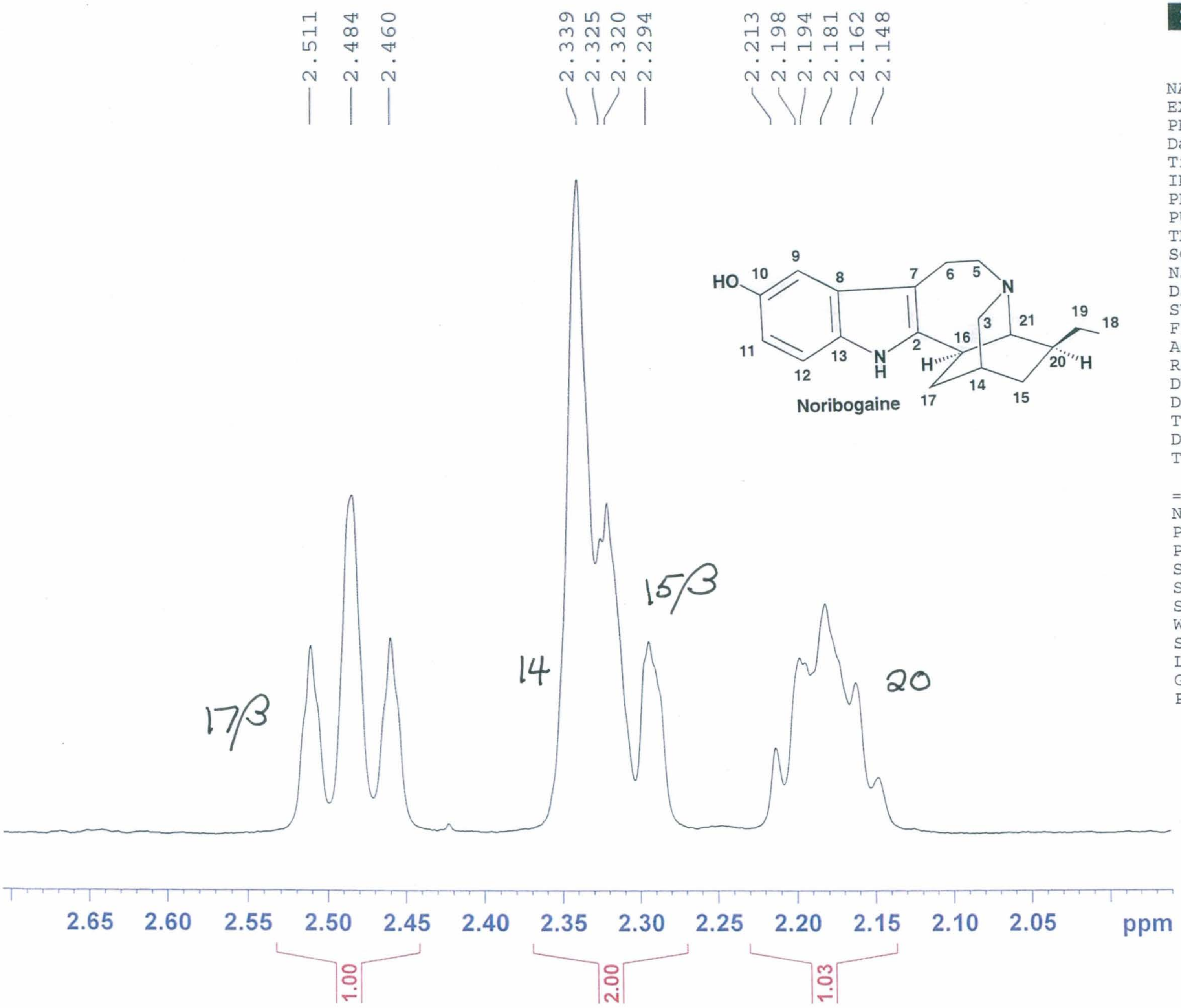

NAME	zhp297
EXPNO	1
PROCNO	1
Date_	20110504
Time	16.43
INSTRUM	spect
PROBHD	5 mm TXI 1H/D-
PULPROG	zg30
TD	65536
SOLVENT	MeOD
NS	16
DS	2
SWH	10330.578 Hz
FIDRES	0.157632 Hz
AQ	3.1720407 sec
RG	161.3
DW	48.400 usec
DE	6.00 usec
TE	300.2 K
D1	1.00000000 sec
TD0	1

======== CHANNEL f1 ========

NUC1	1H
P1	8.65 usec
PL1	-2.00 dB
SFO1	500.1330885 MHz
SI	32768
SF	500.1299157 MHz
WDW	EM
SSB	0
LB	0.30 Hz
GB	0
PC	1.00

Noribogaine source C

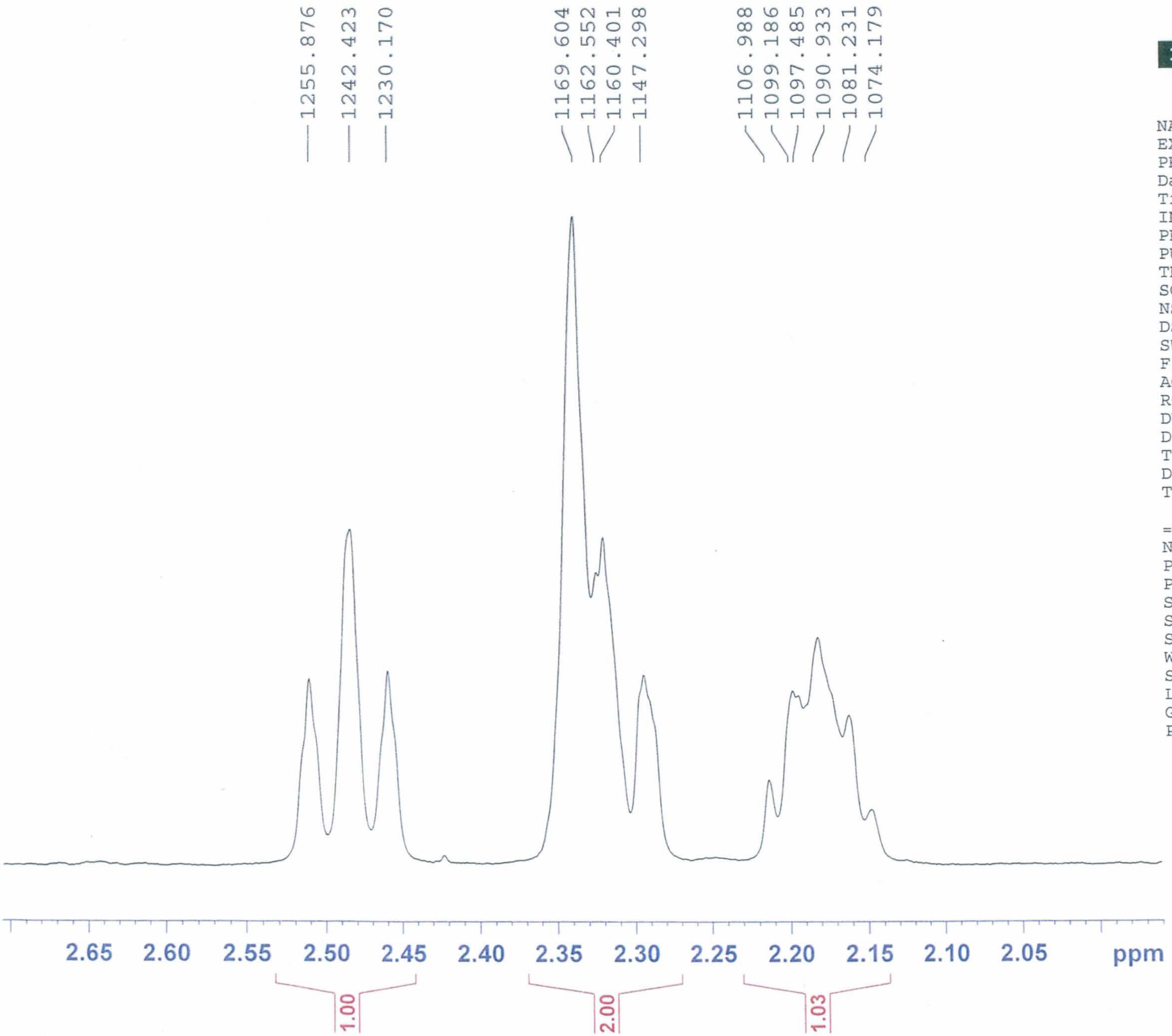

NAME	zhp297	
EXPNO	1	
PROCNO	1	
Date_	20110504	
Time	16.43	
INSTRUM	spect	
PROBHD	5 mm TXI 1H/D-	
PULPROG	zg30	
TD	65536	
SOLVENT	MeOD	
NS	16	
DS	2	
SWH	10330.578	Hz
FIDRES	0.157632	Hz
AQ	3.1720407	sec
RG	161.3	
DW	48.400	usec
DE	6.00	usec
TE	300.2	K
D1	1.00000000	sec
TD0	1	

======== CHANNEL f1 ========

NUC1	1H	
P1	8.65	usec
PL1	-2.00	dB
SFO1	500.1330885	MHz
SI	32768	
SF	500.1299157	MHz
WDW	EM	
SSB	0	
LB	0.30	Hz
GB	0	
PC	1.00	

Noribogaine source C

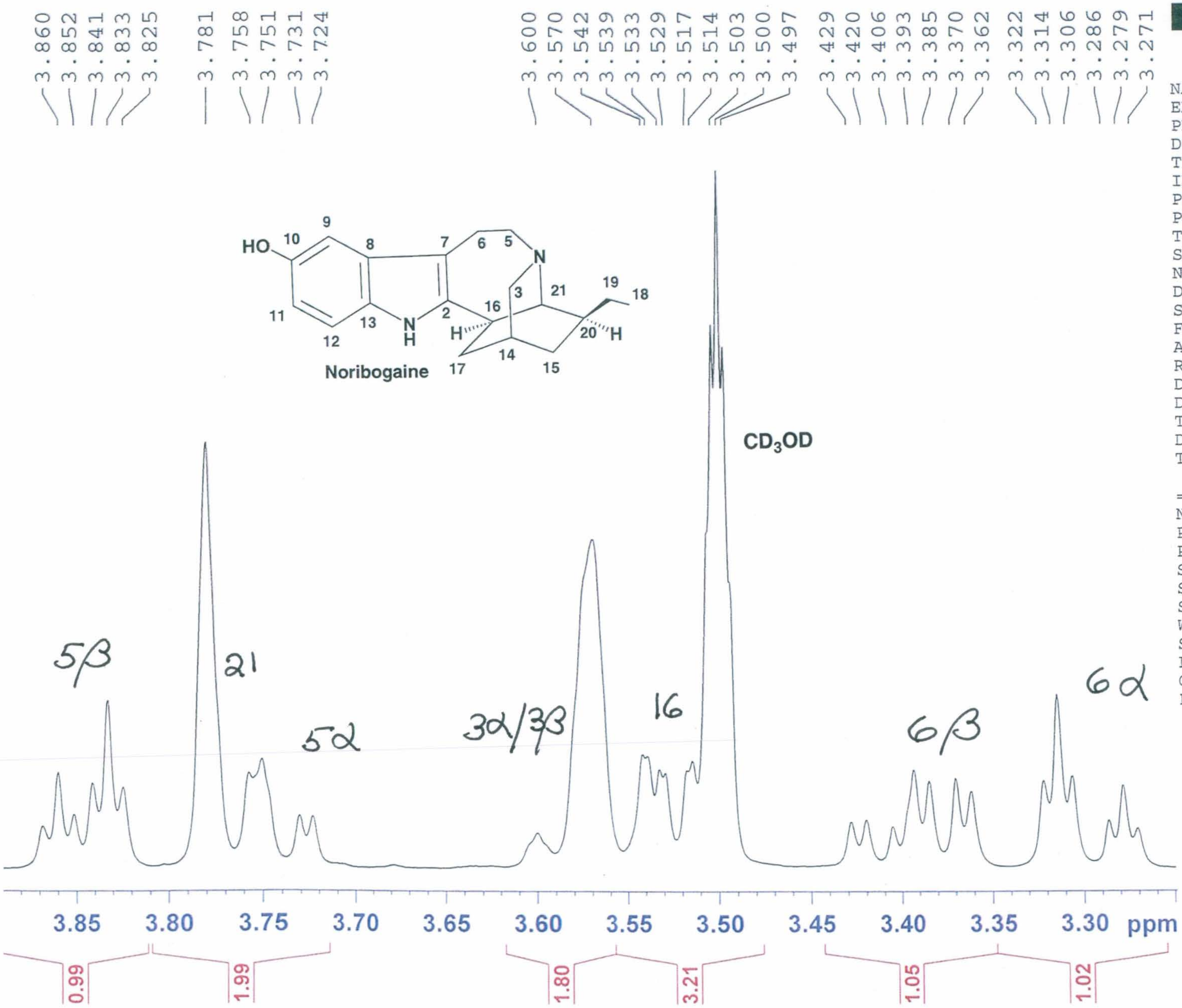

BRUKER

NAME	zhp297
EXPNO	1
PROCNO	1
Date_	20110504
Time	16.43
INSTRUM	spect
PROBHD	5 mm TXI 1H/D-
PULPROG	zg30
TD	65536
SOLVENT	MeOD
NS	16
DS	2
SWH	10330.578 Hz
FIDRES	0.157632 Hz
AQ	3.1720407 sec
RG	161.3
DW	48.400 usec
DE	6.00 usec
TE	300.2 K
D1	1.00000000 sec
TD0	1

======== CHANNEL f1 ========

NUC1	1H
P1	8.65 usec
PL1	-2.00 dB
SFO1	500.1330885 MHz
SI	32768
SF	500.1299157 MHz
WDW	EM
SSB	0
LB	0.30 Hz
GB	0
PC	1.00

Noribogaine source C

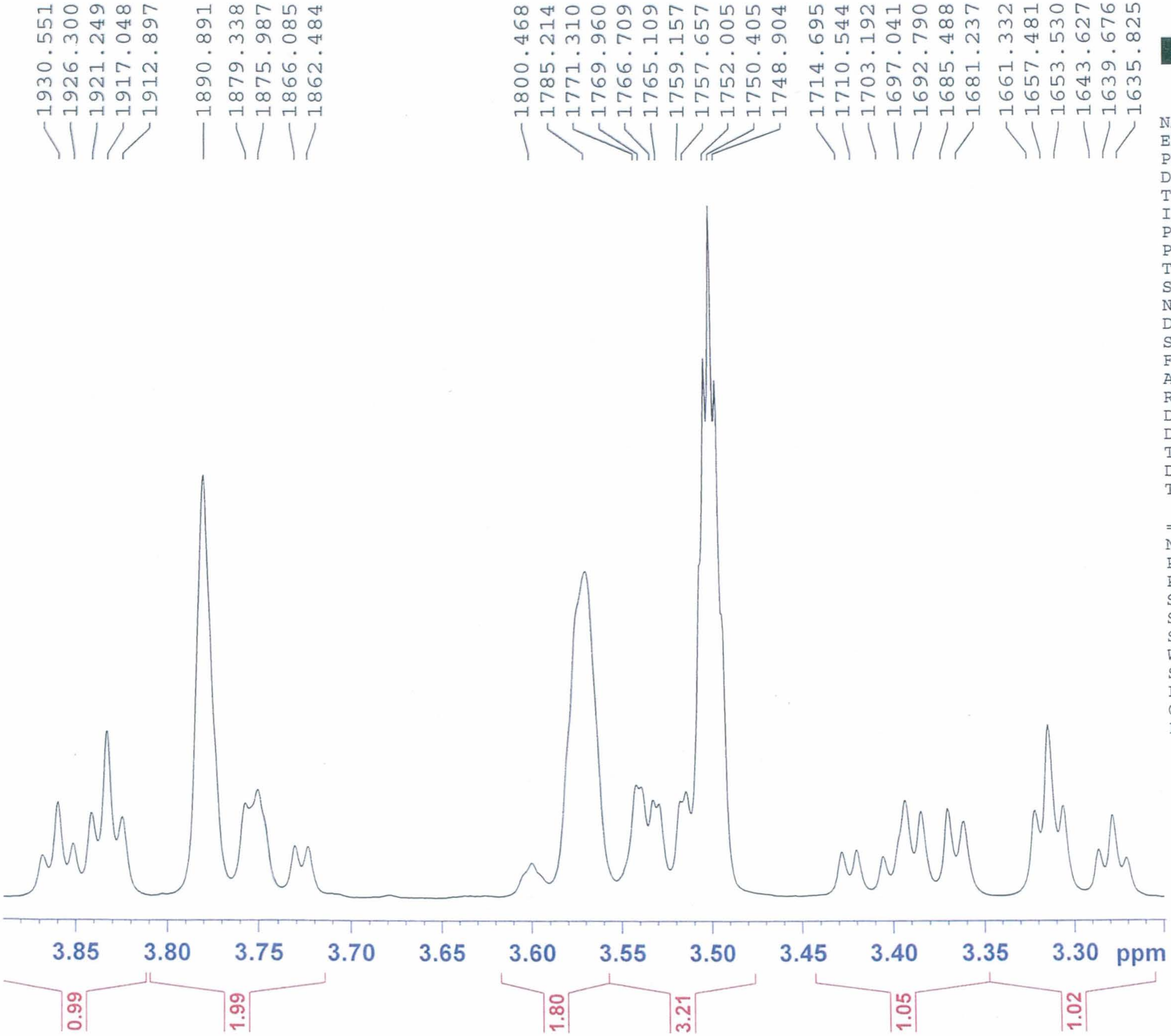

NAME	zhp297
EXPNO	1
PROCNO	1
Date_	20110504
Time	16.43
INSTRUM	spect
PROBHD	5 mm TXI 1H/D-
PULPROG	zg30
TD	65536
SOLVENT	MeOD
NS	16
DS	2
SWH	10330.578 Hz
FIDRES	0.157632 Hz
AQ	3.1720407 sec
RG	161.3
DW	48.400 usec
DE	6.00 usec
TE	300.2 K
D1	1.00000000 sec
TD0	1

======== CHANNEL f1 ========

NUC1	1H
P1	8.65 usec
PL1	-2.00 dB
SFO1	500.1330885 MHz
SI	32768
SF	500.1299157 MHz
WDW	EM
SSB	0
LB	0.30 Hz
GB	0
PC	1.00

Noribogaine source C

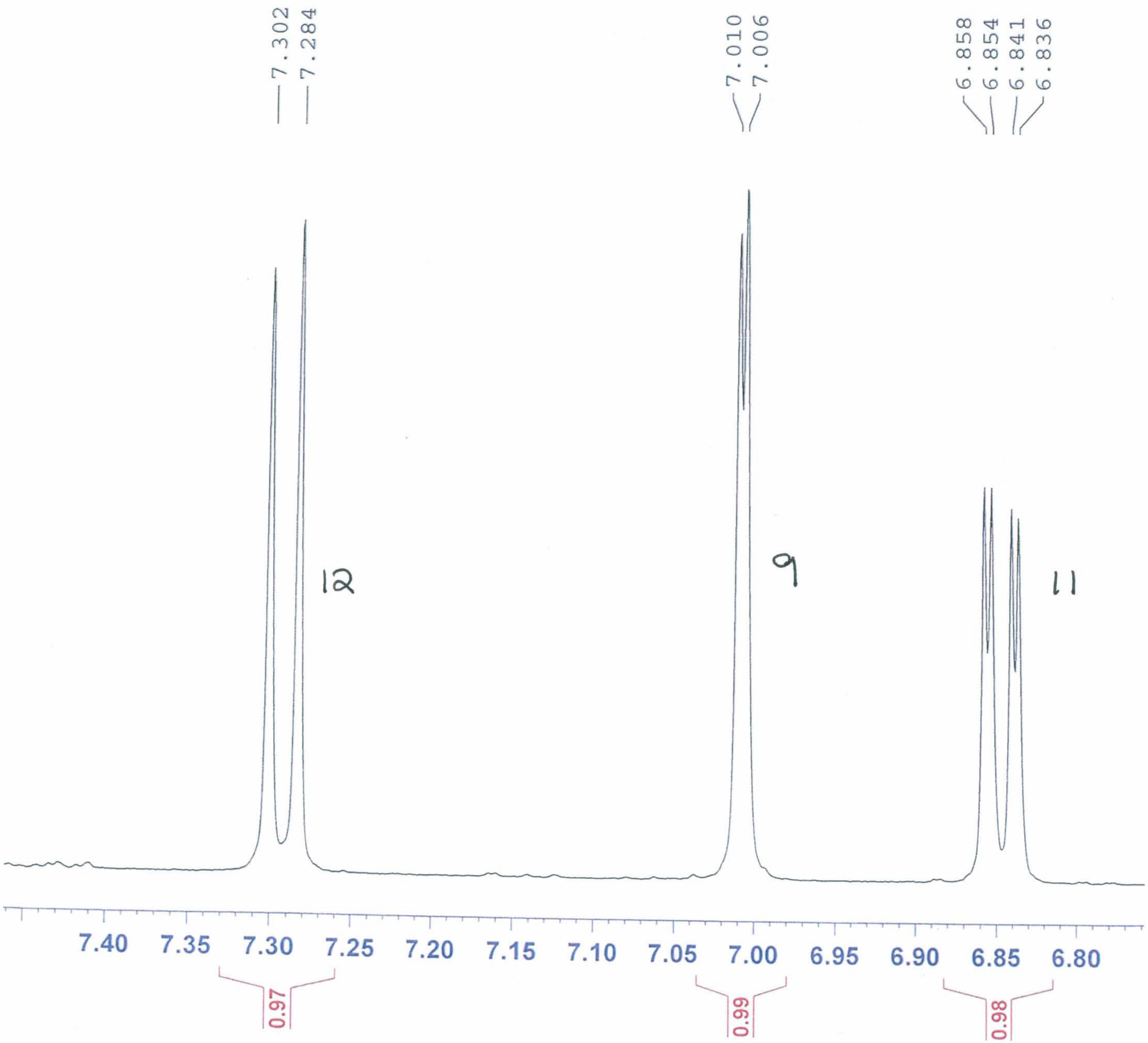

BRUKER

NAME	zhp297
EXPNO	1
PROCNO	1
Date_	20110504
Time	16.43
INSTRUM	spect
PROBHD	5 mm TXI 1H/D-
PULPROG	zg30
TD	65536
SOLVENT	MeOD
NS	16
DS	2
SWH	10330.578 Hz
FIDRES	0.157632 Hz
AQ	3.1720407 sec
RG	161.3
DW	48.400 usec
DE	6.00 usec
TE	300.2 K
D1	1.00000000 sec
TD0	1

======== CHANNEL f1 ========

NUC1	1H
P1	8.65 usec
PL1	-2.00 dB
SFO1	500.1330885 MHz
SI	32768
SF	500.1299157 MHz
WDW	EM
SSB	0
LB	0.30 Hz
GB	0
PC	1.00

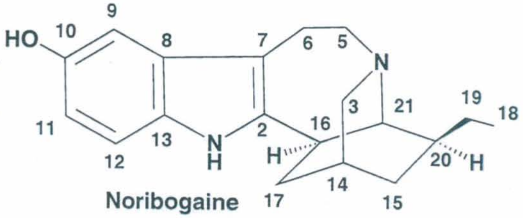

Noribogaine

Noribogaine source C

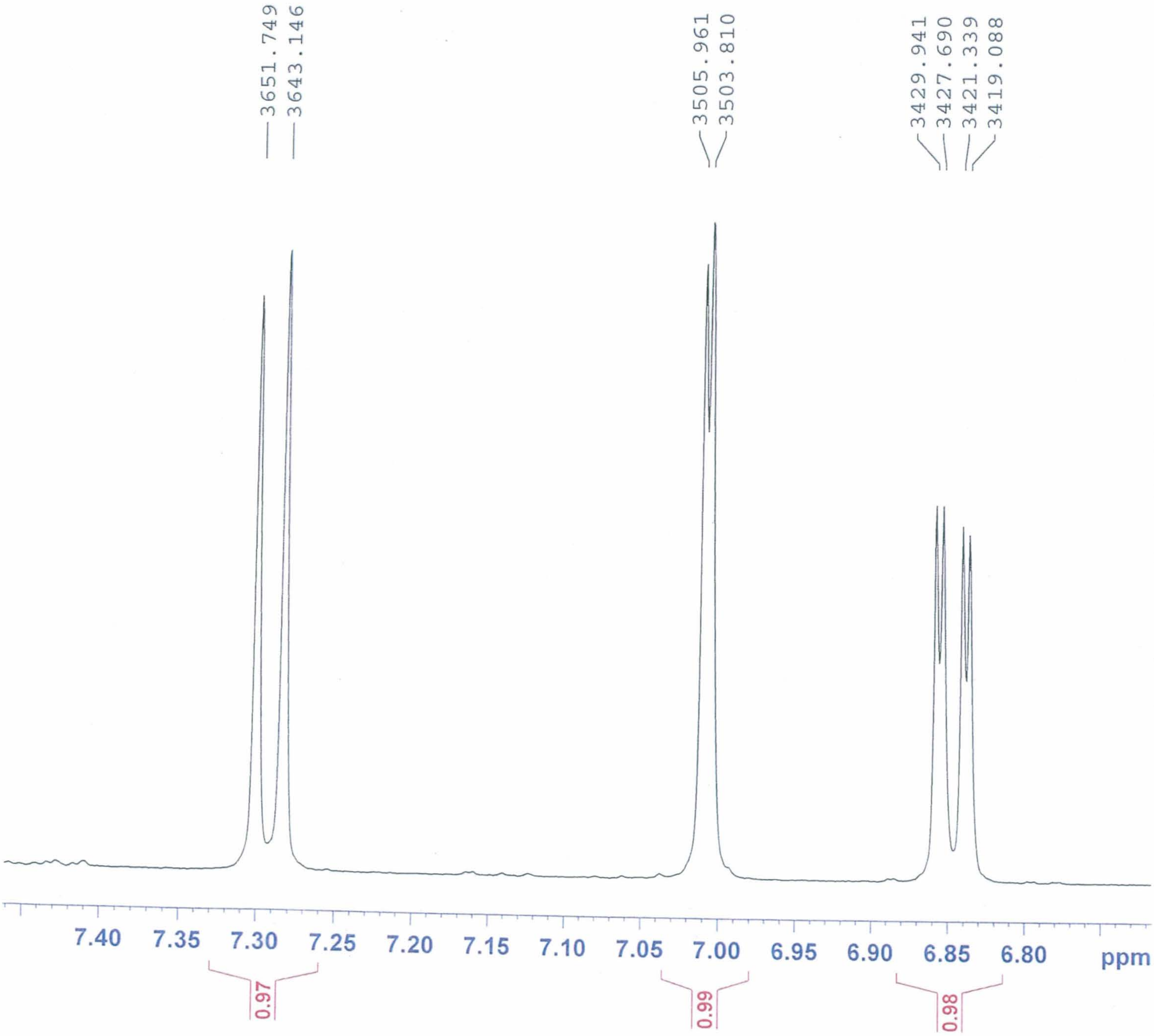

BRUKER

NAME	zhp297
EXPNO	1
PROCNO	1
Date_	20110504
Time	16.43
INSTRUM	spect
PROBHD	5 mm TXI 1H/D-
PULPROG	zg30
TD	65536
SOLVENT	MeOD
NS	16
DS	2
SWH	10330.578 Hz
FIDRES	0.157632 Hz
AQ	3.1720407 sec
RG	161.3
DW	48.400 usec
DE	6.00 usec
TE	300.2 K
D1	1.00000000 sec
TD0	1

======== CHANNEL f1 ========

NUC1	1H
P1	8.65 usec
PL1	-2.00 dB
SFO1	500.1330885 MHz
SI	32768
SF	500.1299157 MHz
WDW	EM
SSB	0
LB	0.30 Hz
GB	0
PC	1.00

Noribogaine source C

martin 297

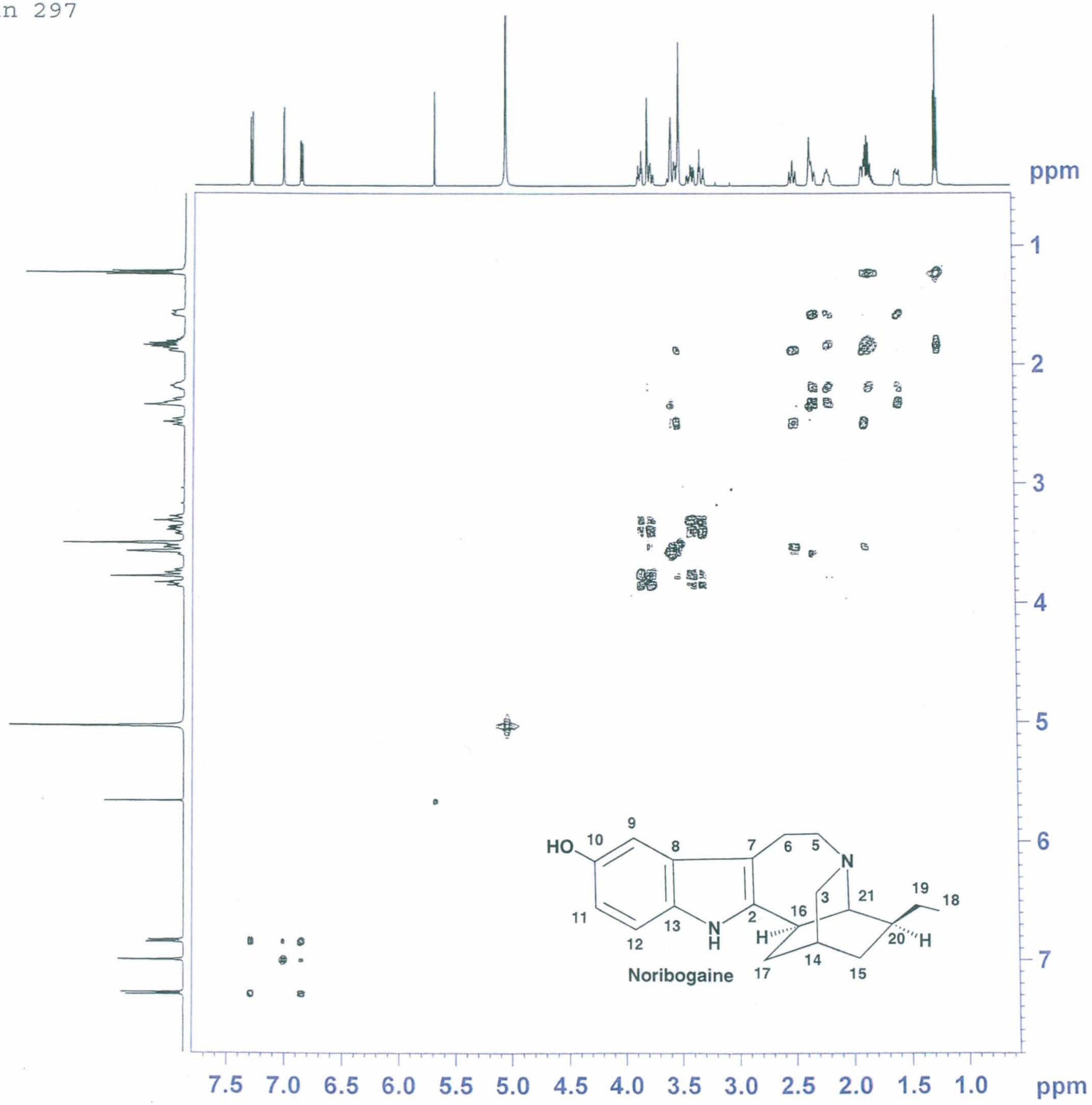

NAME	zhp297
EXPNO	4
PROCNO	1
Date_	20110504
Time	21.08
INSTRUM	spect
PROBHD	5 mm TXI 1H/D-
PULPROG	cosygpqf
TD	2048
SOLVENT	MeOD
NS	1
DS	8
SWH	3623.188 Hz
FIDRES	1.769135 Hz
AQ	0.2828120 sec
RG	64
DW	138.000 usec
DE	6.00 usec
TE	301.2 K
d0	0.00000300 sec
D1	1.35786796 sec
d13	0.00000400 sec
D16	0.00020000 sec
IN0	0.00027600 sec
======== CHANNEL f1 ========	
NUC1	1H
P0	8.65 usec
P1	8.65 usec
PL1	-2.00 dB
SFO1	500.1320027 MHz
====== GRADIENT CHANNEL =====	
GPNAM1	SINE.100
GPNAM2	SINE.100
GPZ1	10.00 %
GPZ2	10.00 %
P16	1000.00 usec
ND0	1
TD	128
SFO1	500.132 MHz
FIDRES	28.306160 Hz
SW	7.244 ppm
FnMODE	QF
SI	1024
SF	500.1299147 MHz
WDW	SINE
SSB	0
LB	0.00 Hz
GB	0
PC	1.40
SI	1024
MC2	QF
SF	500.1299131 MHz
WDW	SINE
SSB	0
LB	0.00 Hz
GB	0

Noribogaine source C

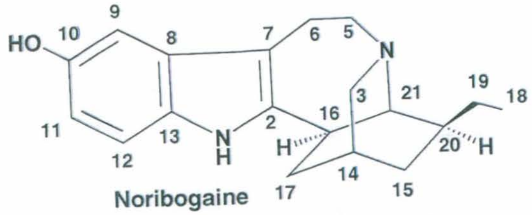

Noribogaine

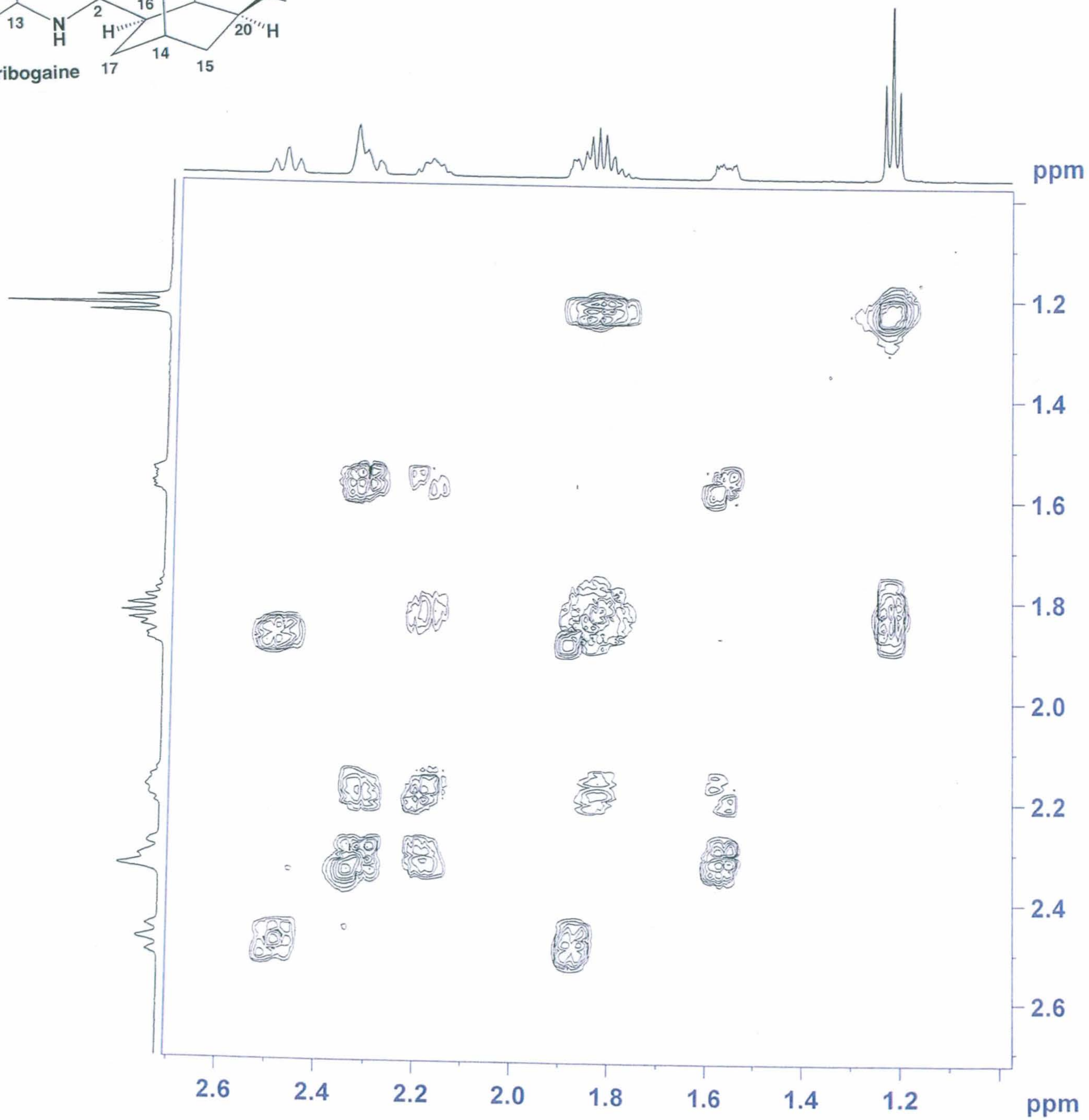

BRUKER

NAME	zhp297	
EXPNO	4	
PROCNO	1	
Date_	20110504	
Time	21.08	
INSTRUM	spect	
PROBHD	5 mm TXI 1H/D-	
PULPROG	cosygpqf	
TD	2048	
SOLVENT	MeOD	
NS	1	
DS	8	
SWH	3623.188	Hz
FIDRES	1.769135	Hz
AQ	0.2828120	sec
RG	64	
DW	138.000	usec
DE	6.00	usec
TE	301.2	K
d0	0.00000300	sec
D1	1.35786796	sec
d13	0.00000400	sec
D16	0.00020000	sec
IN0	0.00027600	sec
======== CHANNEL f1 ========		
NUC1	1H	
P0	8.65	usec
P1	8.65	usec
PL1	-2.00	dB
SFO1	500.1320027	MHz
====== GRADIENT CHANNEL =====		
GPNAM1	SINE.100	
GPNAM2	SINE.100	
GPZ1	10.00	%
GPZ2	10.00	%
P16	1000.00	usec
ND0	1	
TD	128	
SFO1	500.132	MHz
FIDRES	28.306160	Hz
SW	7.244	ppm
FnMODE	QF	
SI	1024	
SF	500.1299147	MHz
WDW	SINE	
SSB	0	
LB	0.00	Hz
GB	0	
PC	1.40	
SI	1024	
MC2	QF	
SF	500.1299131	MHz
WDW	SINE	
SSB	0	
LB	0.00	Hz
GB	0	

Noribogaine source C

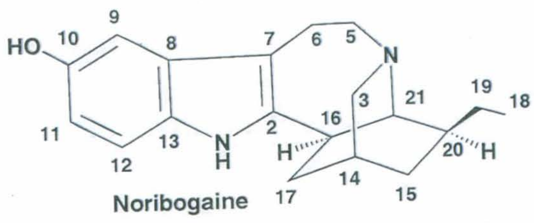

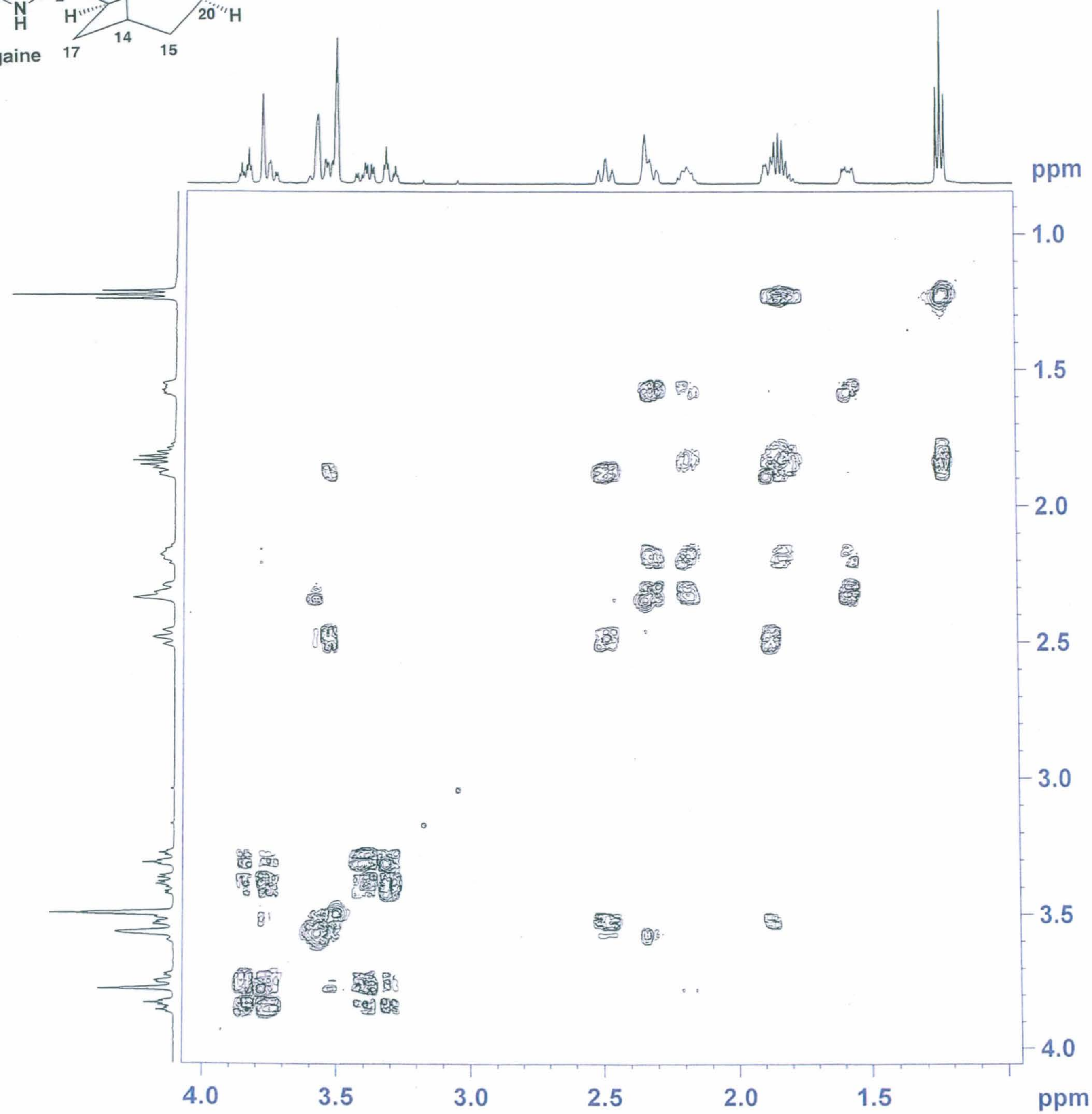

BRUKER

```
NAME            zhp297
EXPNO                4
PROCNO               1
Date_         20110504
Time             21.08
INSTRUM          spect
PROBHD   5 mm TXI 1H/D-
PULPROG      cosygpqf
TD                2048
SOLVENT           MeOD
NS                   1
DS                   8
SWH           3623.188 Hz
FIDRES        1.769135 Hz
AQ           0.2828120 sec
RG                  64
DW             138.000 usec
DE                6.00 usec
TE               301.2 K
d0          0.00000300 sec
D1          1.35786796 sec
d13         0.00000400 sec
D16         0.00020000 sec
IN0         0.00027600 sec

======== CHANNEL f1 ========
NUC1                1H
P0                8.65 usec
P1                8.65 usec
PL1              -2.00 dB
SFO1       500.1320027 MHz

====== GRADIENT CHANNEL =====
GPNAM1       SINE.100
GPNAM2       SINE.100
GPZ1            10.00 %
GPZ2            10.00 %
P16           1000.00 usec
ND0                 1
TD                128
SFO1          500.132 MHz
FIDRES      28.306160 Hz
SW              7.244 ppm
FnMODE             QF
SI               1024
SF        500.1299147 MHz
WDW              SINE
SSB                 0
LB               0.00 Hz
GB                  0
PC               1.40
SI               1024
MC2                QF
SF        500.1299131 MHz
WDW              SINE
SSB                 0
LB               0.00 Hz
GB                  0
```

Noribogaine source C

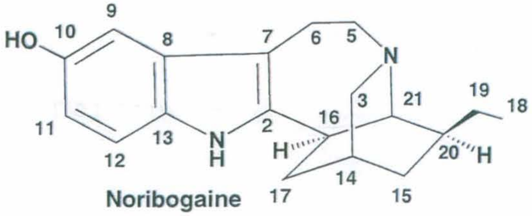

Noribogaine

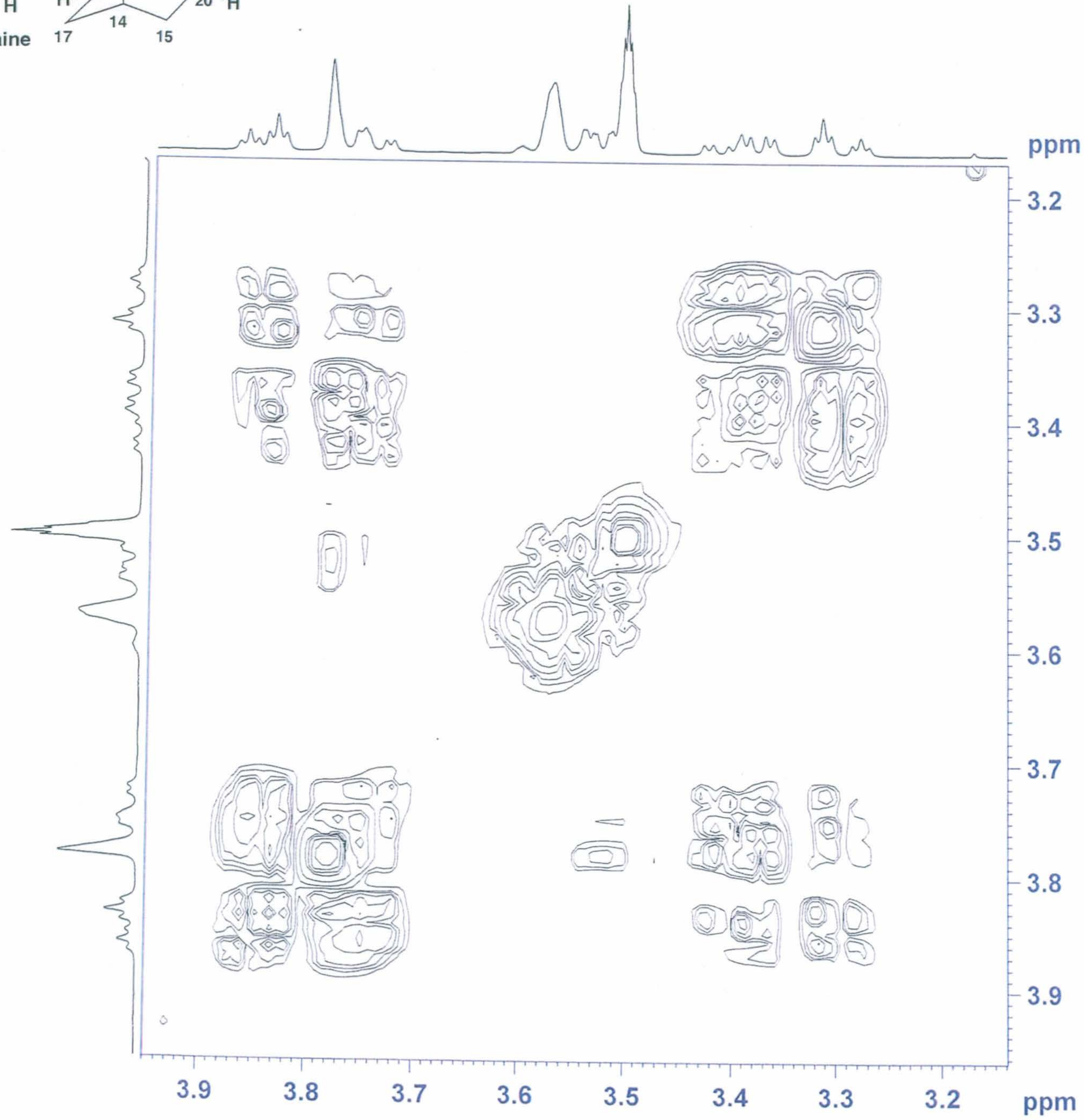

BRUKER

```
NAME           zhp297
EXPNO               4
PROCNO              1
Date_        20110504
Time            21.08
INSTRUM         spect
PROBHD   5 mm TXI 1H/D-
PULPROG      cosygpqf
TD               2048
SOLVENT          MeOD
NS                  1
DS                  8
SWH          3623.188 Hz
FIDRES       1.769135 Hz
AQ          0.2828120 sec
RG                 64
DW            138.000 usec
DE               6.00 usec
TE              301.2 K
d0         0.00000300 sec
D1         1.35786796 sec
d13        0.00000400 sec
D16        0.00020000 sec
IN0        0.00027600 sec

======== CHANNEL f1 ========
NUC1               1H
P0               8.65 usec
P1               8.65 usec
PL1             -2.00 dB
SFO1      500.1320027 MHz

====== GRADIENT CHANNEL =====
GPNAM1       SINE.100
GPNAM2       SINE.100
GPZ1            10.00 %
GPZ2            10.00 %
P16           1000.00 usec
ND0                 1
TD                128
SFO1          500.132 MHz
FIDRES      28.306160 Hz
SW              7.244 ppm
FnMODE             QF
SI               1024
SF        500.1299147 MHz
WDW              SINE
SSB                 0
LB               0.00 Hz
GB                  0
PC               1.40
SI               1024
MC2                QF
SF        500.1299131 MHz
WDW              SINE
SSB                 0
LB               0.00 Hz
GB                  0
```

Noribogaine source C

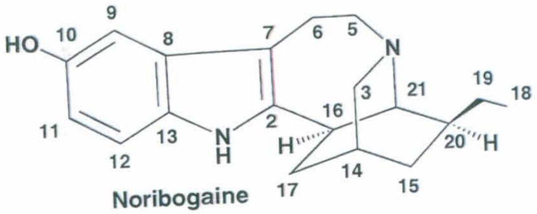

Noribogaine

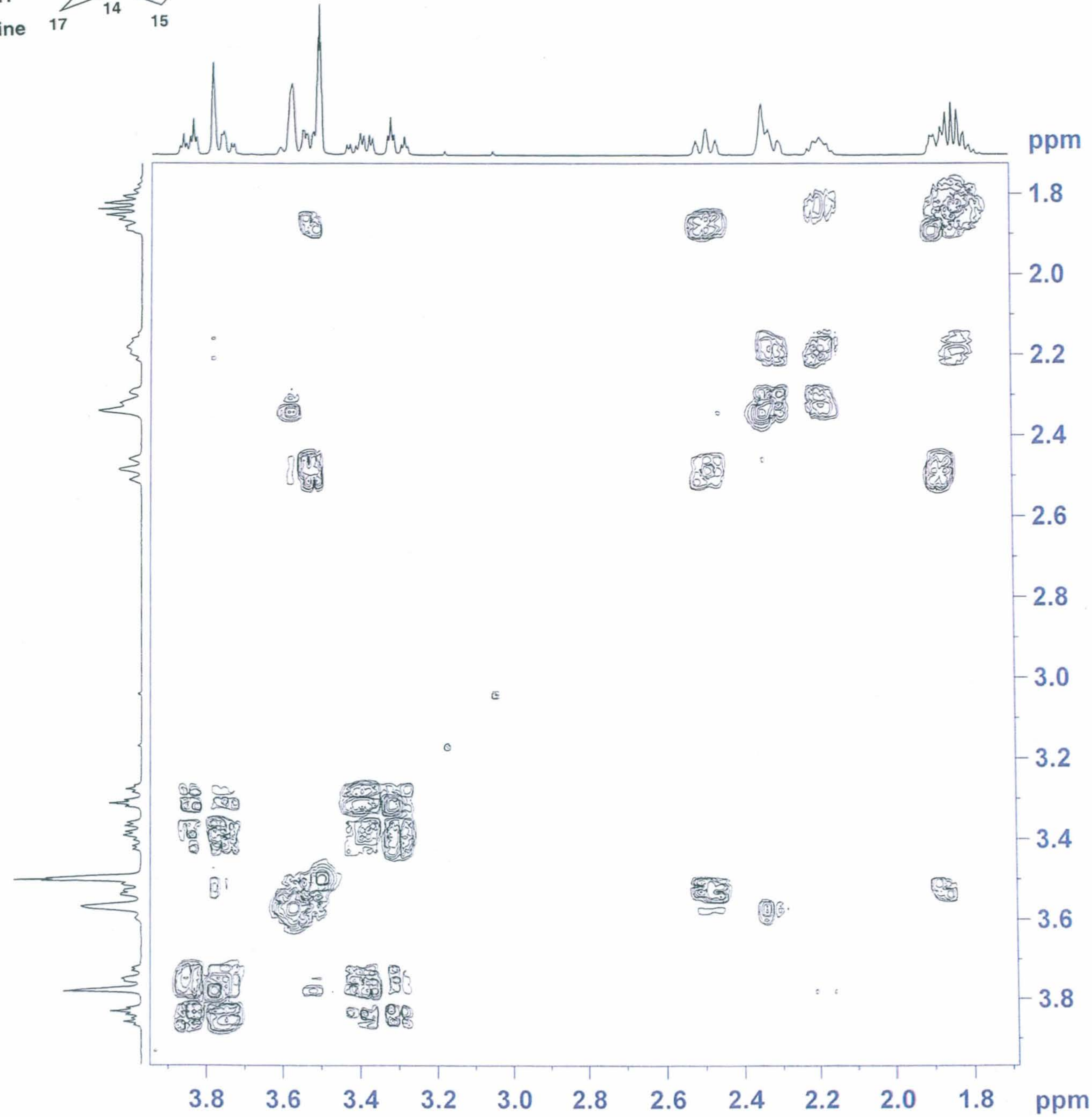

NAME	zhp297
EXPNO	4
PROCNO	1
Date_	20110504
Time	21.08
INSTRUM	spect
PROBHD	5 mm TXI 1H/D-
PULPROG	cosygpqf
TD	2048
SOLVENT	MeOD
NS	1
DS	8
SWH	3623.188 Hz
FIDRES	1.769135 Hz
AQ	0.2828120 sec
RG	64
DW	138.000 usec
DE	6.00 usec
TE	301.2 K
d0	0.00000300 sec
D1	1.35786796 sec
d13	0.00000400 sec
D16	0.00020000 sec
IN0	0.00027600 sec
======== CHANNEL f1 ========	
NUC1	1H
P0	8.65 usec
P1	8.65 usec
PL1	-2.00 dB
SFO1	500.1320027 MHz
====== GRADIENT CHANNEL =====	
GPNAM1	SINE.100
GPNAM2	SINE.100
GPZ1	10.00 %
GPZ2	10.00 %
P16	1000.00 usec
ND0	1
TD	128
SFO1	500.132 MHz
FIDRES	28.306160 Hz
SW	7.244 ppm
FnMODE	QF
SI	1024
SF	500.1299147 MHz
WDW	SINE
SSB	0
LB	0.00 Hz
GB	0
PC	1.40
SI	1024
MC2	QF
SF	500.1299131 MHz
WDW	SINE
SSB	0
LB	0.00 Hz
GB	0

Noribogaine source C

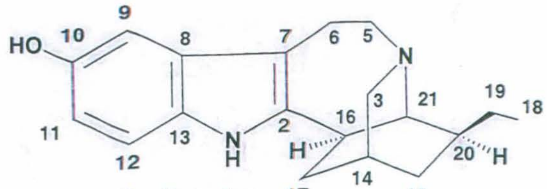

Noribogaine

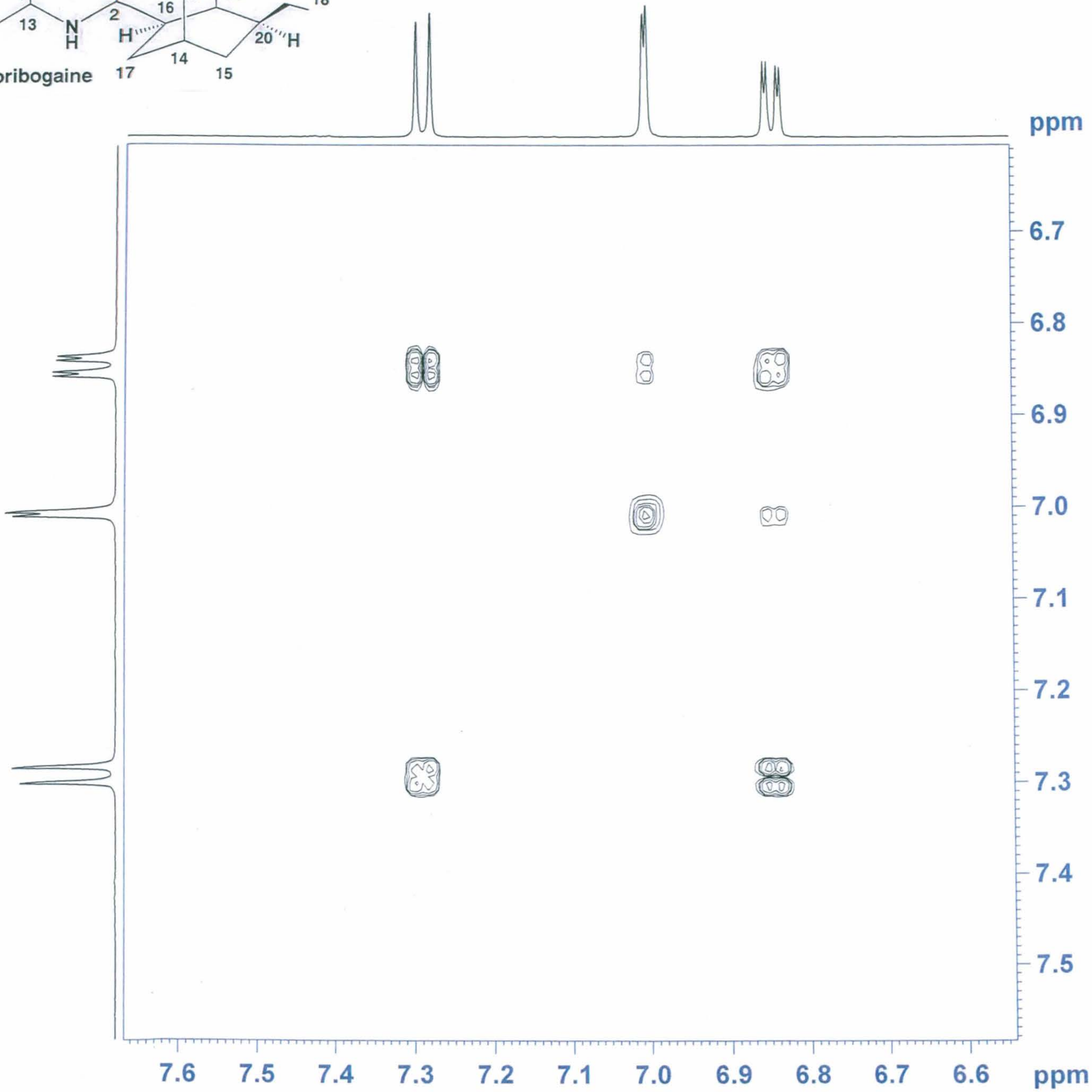

NAME zhp297
EXPNO 4
PROCNO 1
Date_ 20110504
Time 21.08
INSTRUM spect
PROBHD 5 mm TXI 1H/D-
PULPROG cosygpqf
TD 2048
SOLVENT MeOD
NS 1
DS 8
SWH 3623.188 Hz
FIDRES 1.769135 Hz
AQ 0.2828120 sec
RG 64
DW 138.000 usec
DE 6.00 usec
TE 301.2 K
d0 0.00000300 sec
D1 1.35786796 sec
d13 0.00000400 sec
D16 0.00020000 sec
IN0 0.00027600 sec

======== CHANNEL f1 ========
NUC1 1H
P0 8.65 usec
P1 8.65 usec
PL1 -2.00 dB
SFO1 500.1320027 MHz

====== GRADIENT CHANNEL =====
GPNAM1 SINE.100
GPNAM2 SINE.100
GPZ1 10.00 %
GPZ2 10.00 %
P16 1000.00 usec
ND0 1
TD 128
SFO1 500.132 MHz
FIDRES 28.306160 Hz
SW 7.244 ppm
FnMODE QF
SI 1024
SF 500.1299147 MHz
WDW SINE
SSB 0
LB 0.00 Hz
GB 0
PC 1.40
SI 1024
MC2 QF
SF 500.1299131 MHz
WDW SINE
SSB 0
LB 0.00 Hz
GB 0

Noribogaine source C

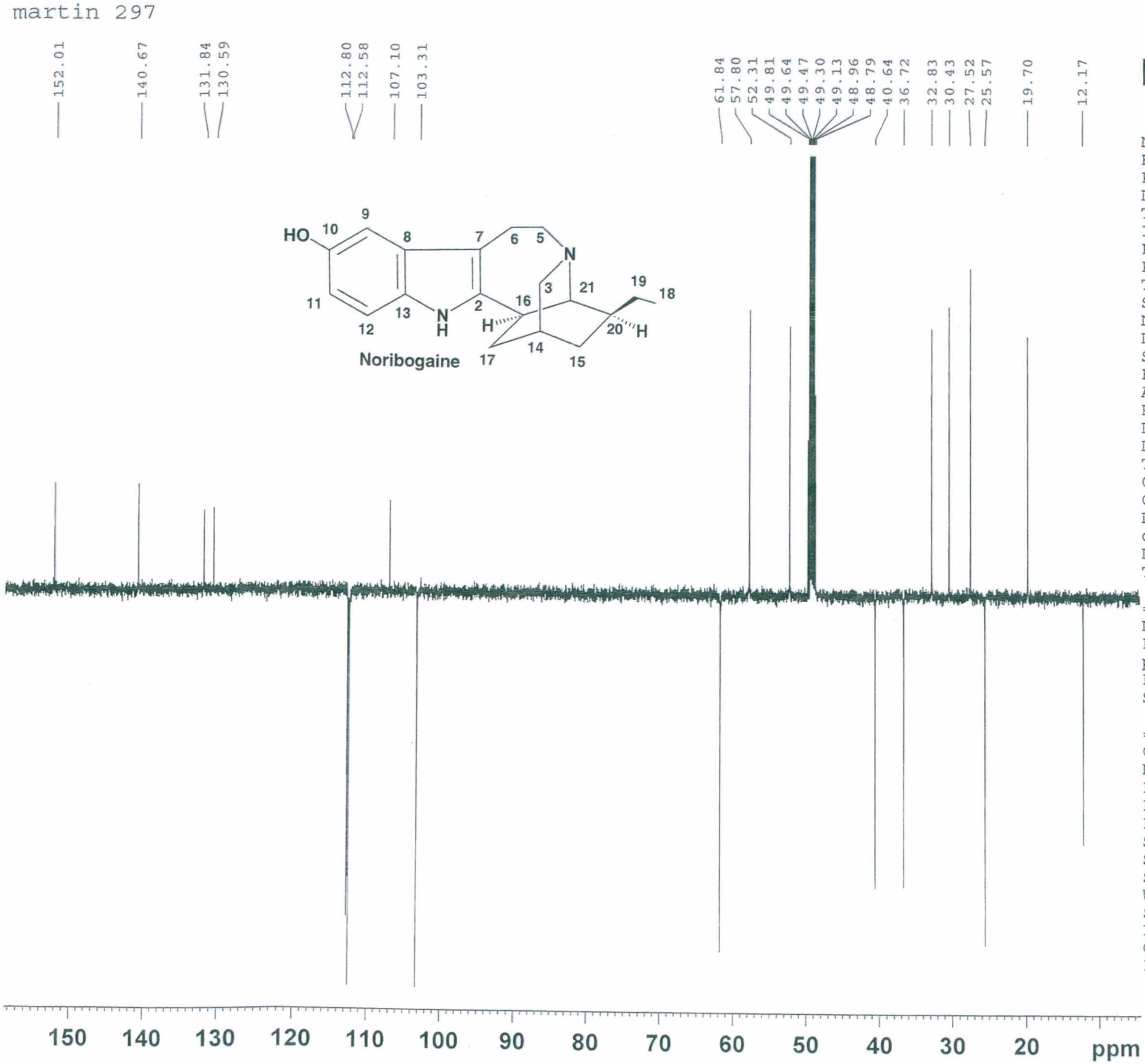

BRUKER

NAME	zhp297
EXPNO	3
PROCNO	1
Date_	20110504
Time	16.51
INSTRUM	spect
PROBHD	5 mm TXI 1H/D-
PULPROG	jmod
TD	65536
SOLVENT	MeOD
NS	5000
DS	4
SWH	30030.029 Hz
FIDRES	0.458222 Hz
AQ	1.0912410 sec
RG	16384
DW	16.650 usec
DE	6.00 usec
TE	301.2 K
CNST2	145.0000000
CNST11	1.0000000
D1	2.00000000 sec
d20	0.00689655 sec
DELTA	0.00001432 sec
TD0	1

======== CHANNEL f1 ========

NUC1	13C
P1	11.25 usec
p2	22.50 usec
PL1	-5.50 dB
SFO1	125.7703643 MHz

======== CHANNEL f2 ========

CPDPRG2	waltz16
NUC2	1H
PCPD2	90.00 usec
PL2	-2.00 dB
PL12	18.50 dB
SFO2	500.1320005 MHz
SI	32768
SF	125.7575757 MHz
WDW	EM
SSB	0
LB	1.00 Hz
GB	0
PC	1.40

Noribogaine source C

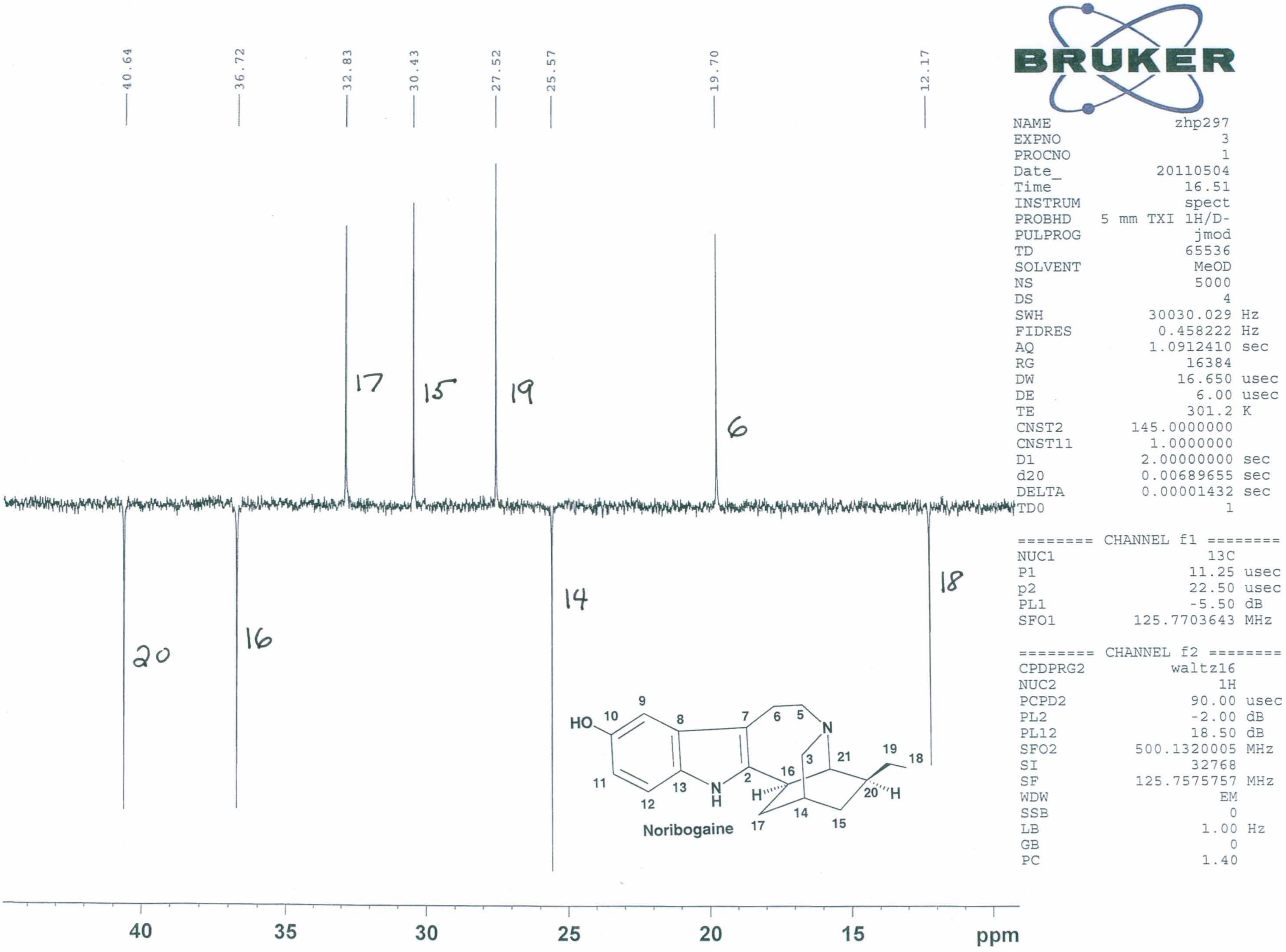

Noribogaine source C

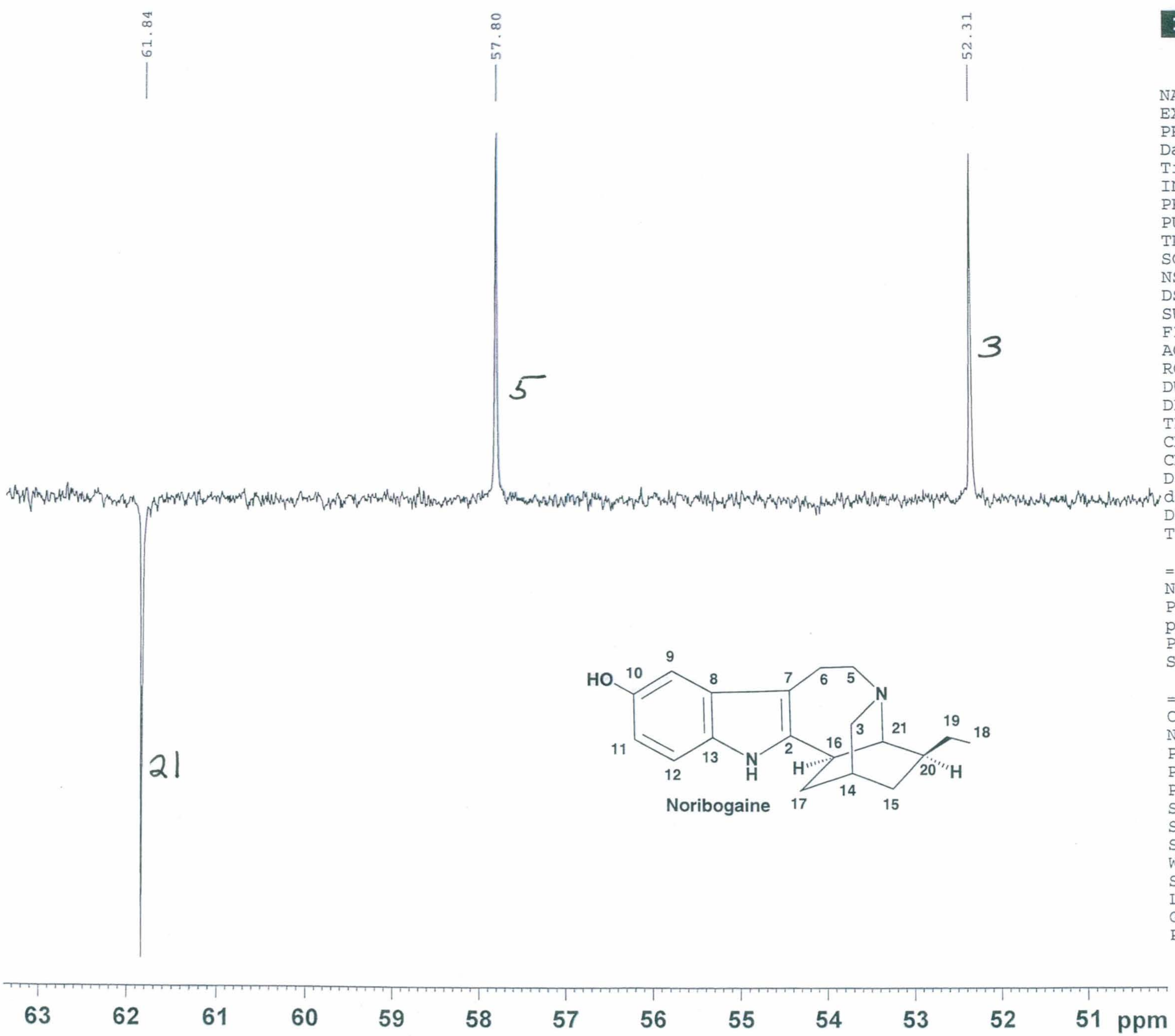

NAME	zhp297
EXPNO	3
PROCNO	1
Date_	20110504
Time	16.51
INSTRUM	spect
PROBHD	5 mm TXI 1H/D-
PULPROG	jmod
TD	65536
SOLVENT	MeOD
NS	5000
DS	4
SWH	30030.029 Hz
FIDRES	0.458222 Hz
AQ	1.0912410 sec
RG	16384
DW	16.650 usec
DE	6.00 usec
TE	301.2 K
CNST2	145.0000000
CNST11	1.0000000
D1	2.00000000 sec
d20	0.00689655 sec
DELTA	0.00001432 sec
TD0	1

======== CHANNEL f1 ========

NUC1	13C
P1	11.25 usec
p2	22.50 usec
PL1	-5.50 dB
SFO1	125.7703643 MHz

======== CHANNEL f2 ========

CPDPRG2	waltz16
NUC2	1H
PCPD2	90.00 usec
PL2	-2.00 dB
PL12	18.50 dB
SFO2	500.1320005 MHz
SI	32768
SF	125.7575757 MHz
WDW	EM
SSB	0
LB	1.00 Hz
GB	0
PC	1.40

Noribogaine source C

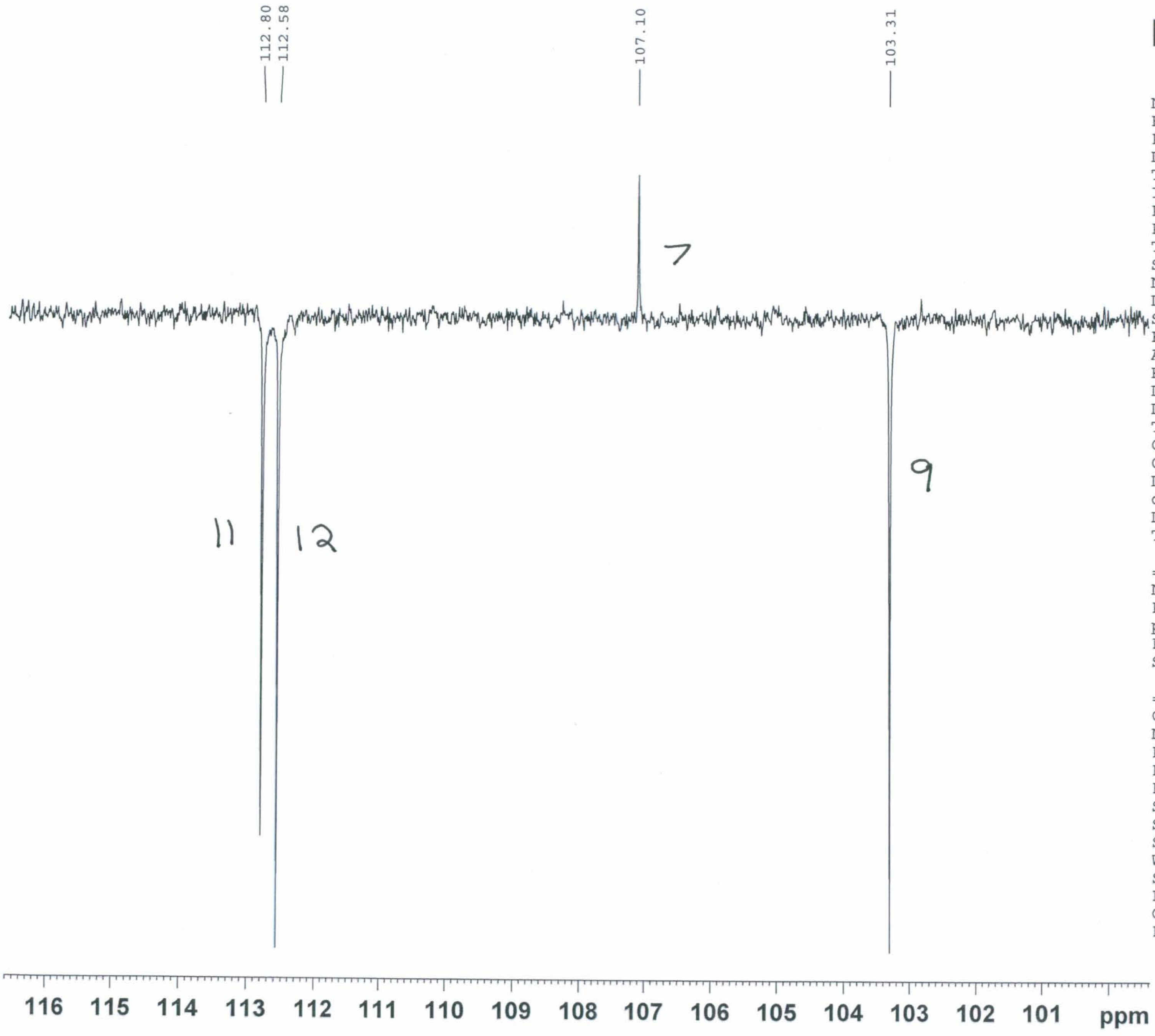

NAME	zhp297
EXPNO	3
PROCNO	1
Date_	20110504
Time	16.51
INSTRUM	spect
PROBHD	5 mm TXI 1H/D-
PULPROG	jmod
TD	65536
SOLVENT	MeOD
NS	5000
DS	4
SWH	30030.029 Hz
FIDRES	0.458222 Hz
AQ	1.0912410 sec
RG	16384
DW	16.650 usec
DE	6.00 usec
TE	301.2 K
CNST2	145.0000000
CNST11	1.0000000
D1	2.00000000 sec
d20	0.00689655 sec
DELTA	0.00001432 sec
TD0	1

======== CHANNEL f1 ========

NUC1	13C
P1	11.25 usec
p2	22.50 usec
PL1	-5.50 dB
SFO1	125.7703643 MHz

======== CHANNEL f2 ========

CPDPRG2	waltz16
NUC2	1H
PCPD2	90.00 usec
PL2	-2.00 dB
PL12	18.50 dB
SFO2	500.1320005 MHz
SI	32768
SF	125.7575757 MHz
WDW	EM
SSB	0
LB	1.00 Hz
GB	0
PC	1.40

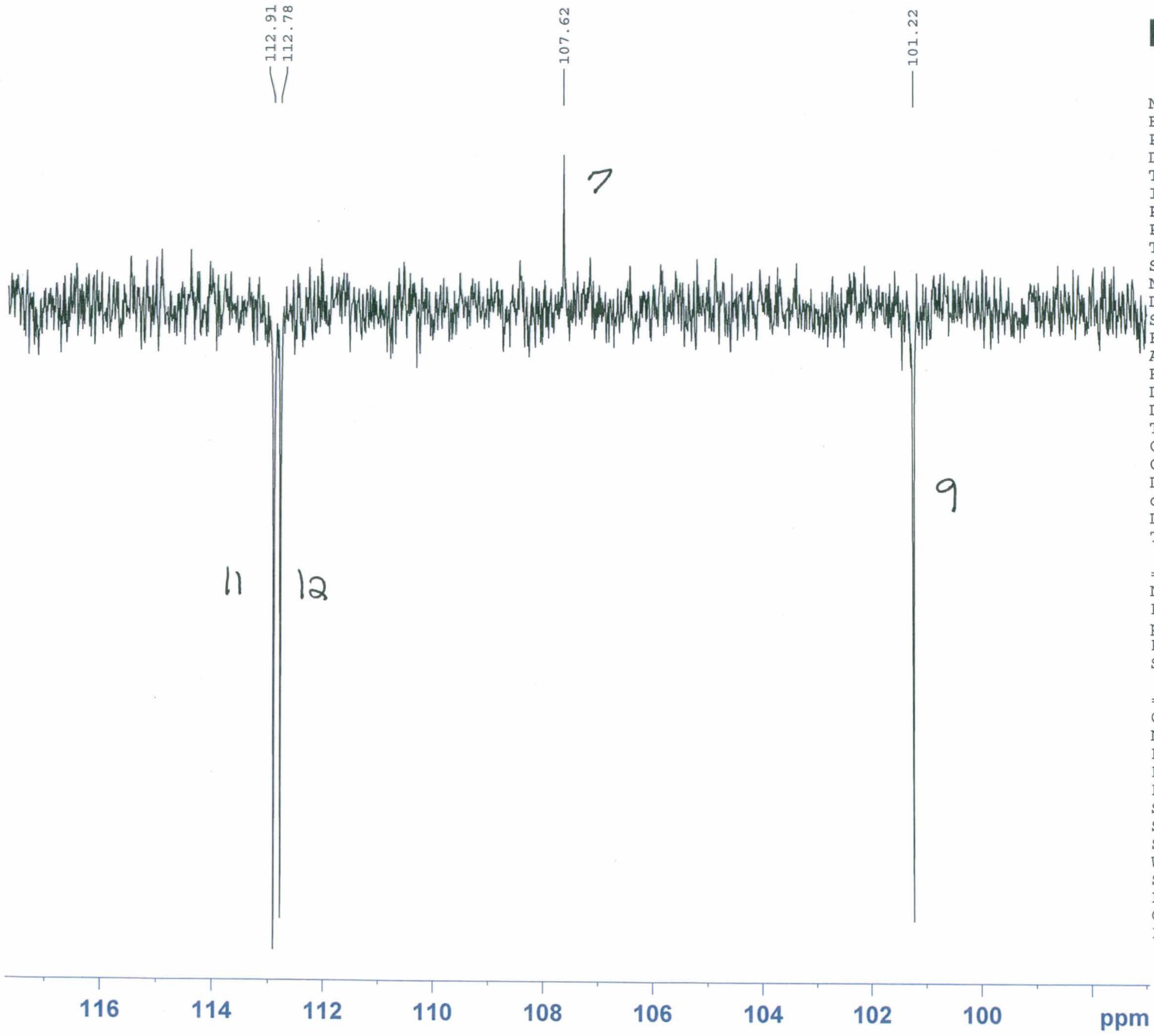

NAME	zhp311	
EXPNO	3	
PROCNO	1	
Date_	20110505	
Time	7.11	
INSTRUM	spect	
PROBHD	5 mm TXI 1H/D-	
PULPROG	jmod	
TD	65536	
SOLVENT	MeOD	
NS	5000	
DS	4	
SWH	30030.029	Hz
FIDRES	0.458222	Hz
AQ	1.0912410	sec
RG	16384	
DW	16.650	usec
DE	6.00	usec
TE	300.2	K
CNST2	145.0000000	
CNST11	1.0000000	
D1	2.00000000	sec
d20	0.00689655	sec
DELTA	0.00001432	sec
TD0	1	
======== CHANNEL f1 ========		
NUC1	13C	
P1	11.25	usec
p2	22.50	usec
PL1	-5.50	dB
SFO1	125.7703643	MHz
======== CHANNEL f2 ========		
CPDPRG2	waltz16	
NUC2	1H	
PCPD2	90.00	usec
PL2	-2.00	dB
PL12	18.50	dB
SFO2	500.1320005	MHz
SI	32768	
SF	125.7575794	MHz
WDW	EM	
SSB	0	
LB	1.00	Hz
GB	0	
PC	1.40	

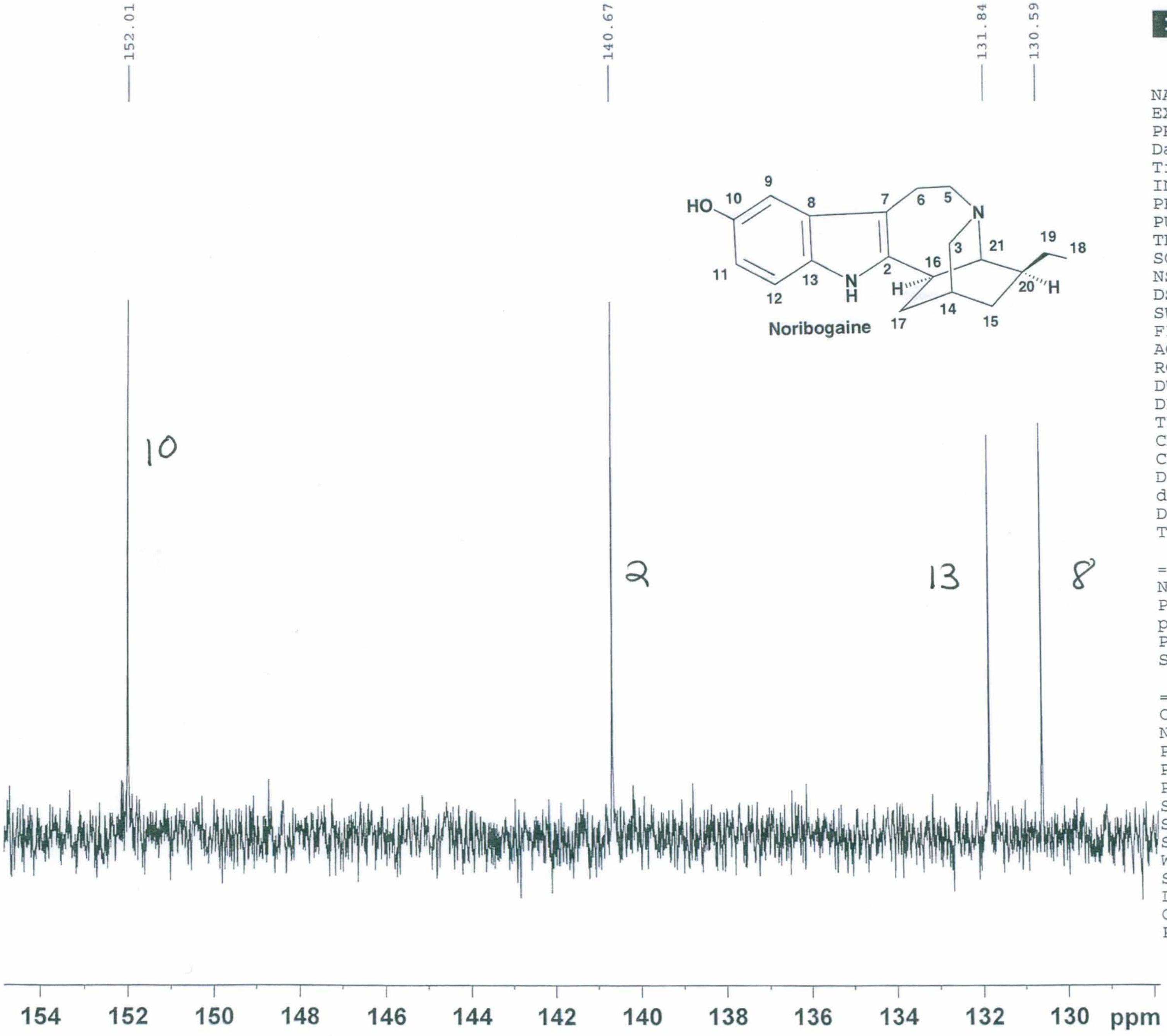

NAME	zhp297	
EXPNO	3	
PROCNO	1	
Date_	20110504	
Time	16.51	
INSTRUM	spect	
PROBHD	5 mm TXI 1H/D-	
PULPROG	jmod	
TD	65536	
SOLVENT	MeOD	
NS	5000	
DS	4	
SWH	30030.029	Hz
FIDRES	0.458222	Hz
AQ	1.0912410	sec
RG	16384	
DW	16.650	usec
DE	6.00	usec
TE	301.2	K
CNST2	145.0000000	
CNST11	1.0000000	
D1	2.00000000	sec
d20	0.00689655	sec
DELTA	0.00001432	sec
TD0	1	
======== CHANNEL f1 ========		
NUC1	13C	
P1	11.25	usec
p2	22.50	usec
PL1	-5.50	dB
SFO1	125.7703643	MHz
======== CHANNEL f2 ========		
CPDPRG2	waltz16	
NUC2	1H	
PCPD2	90.00	usec
PL2	-2.00	dB
PL12	18.50	dB
SFO2	500.1320005	MHz
SI	32768	
SF	125.7575757	MHz
WDW	EM	
SSB	0	
LB	1.00	Hz
GB	0	
PC	1.40	

Noribogaine source C

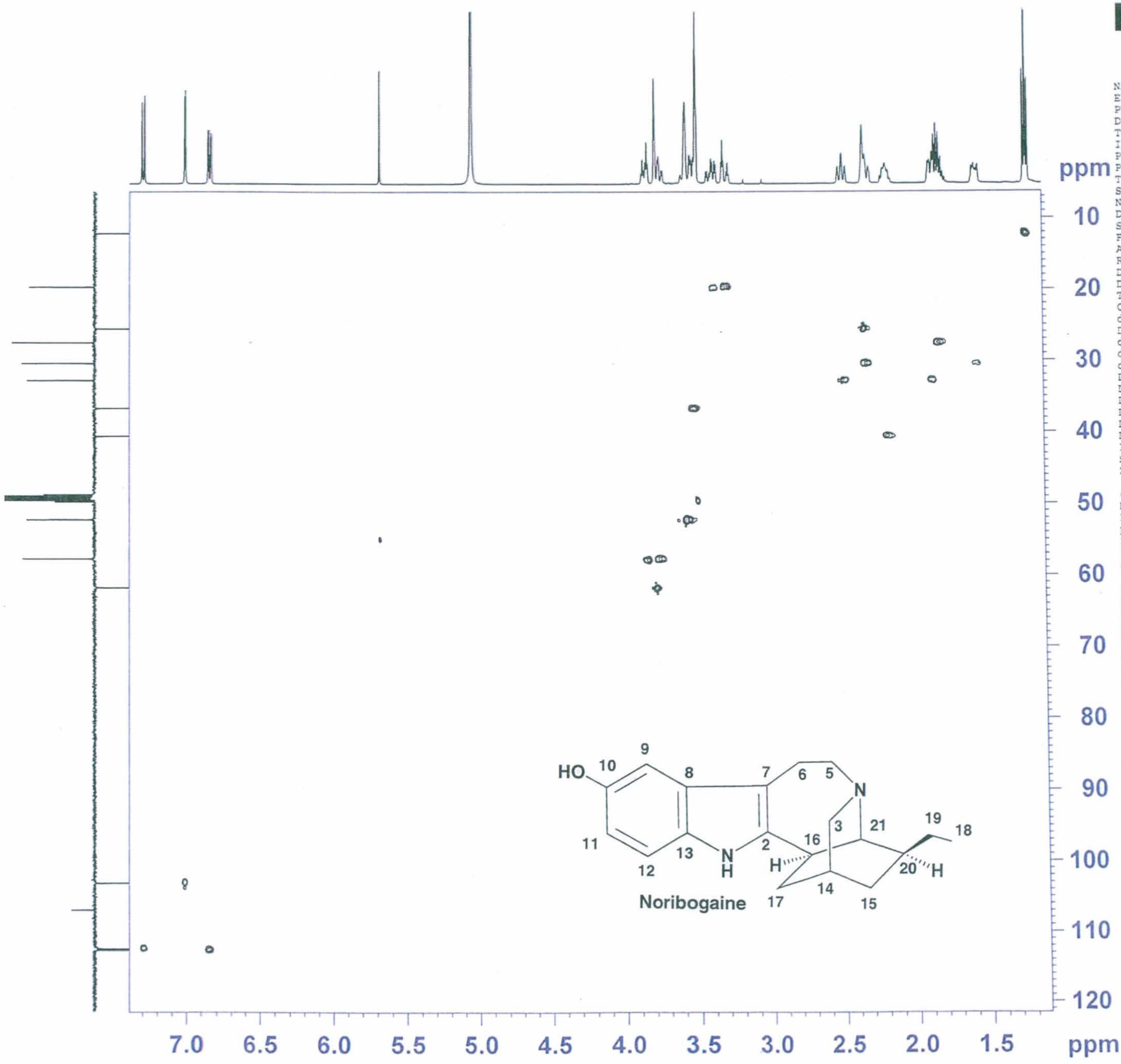

NAME	zhp297	
EXPNO	5	
PROCNO	1	
Date_	20110504	
Time	21.12	
INSTRUM	spect	
PROBHD	5 mm TXI 1H/D-	
PULPROG	hsqcetgpsi2	
TD	1024	
SOLVENT	MeOD	
NS	4	
DS	16	
SWH	3623.188	Hz
FIDRES	3.538270	Hz
AQ	0.1415000	sec
RG	18390.4	
DW	138.000	usec
DE	6.00	usec
TE	301.2	K
CNST2	145.0000000	
d0	0.00000300	sec
D1	1.43548799	sec
d4	0.00172414	sec
d11	0.03000000	sec
d13	0.00000400	sec
D16	0.00020000	sec
D24	0.00086207	sec
DELTA	0.00127330	sec
DELTA1	0.00120800	sec
DELTA2	0.00006207	sec
DELTA3	0.00052414	sec
IN0	0.00002400	sec
ST1CNT	0	
ZGOPTNS		
======== CHANNEL f1 ========		
NUC1	1H	
P1	8.65	usec
p2	17.30	usec
P28	0.10	usec
PL1	-2.00	dB
SFO1	500.1320027	MHz
======== CHANNEL f2 ========		
CPDPRG2	garp	
NUC2	13C	
P3	11.25	usec
p4	22.50	usec
PCPD2	75.00	usec
PL2	-5.50	dB
PL12	10.98	dB
SFO2	125.7672177	MHz
====== GRADIENT CHANNEL =====		
GPNAM1	SINE.100	
GPNAM2	SINE.100	
GPNAM3	SINE.100	
GPNAM4	SINE.100	
GPZ1	80.00	%
GPZ2	20.10	%
GPZ3	11.00	%
GPZ4	-5.00	%
P16	1000.00	usec
P19	600.00	usec
ND0	2	
TD	256	
SFO1	125.7672	MHz
FIDRES	81.380211	Hz
SW	165.650	ppm
FnMODE	Echo-Antiecho	
SI	1024	
SF	500.1299131	MHz
WDW	QSINE	
SSB	2	
LB	0.00	Hz
GB	0	
PC	1.40	
SI	1024	
MC2	echo-antiecho	
SF	125.7575747	MHz
WDW	QSINE	
SSB	2	
LB	0.00	Hz
GB	0	

Noribogaine source C

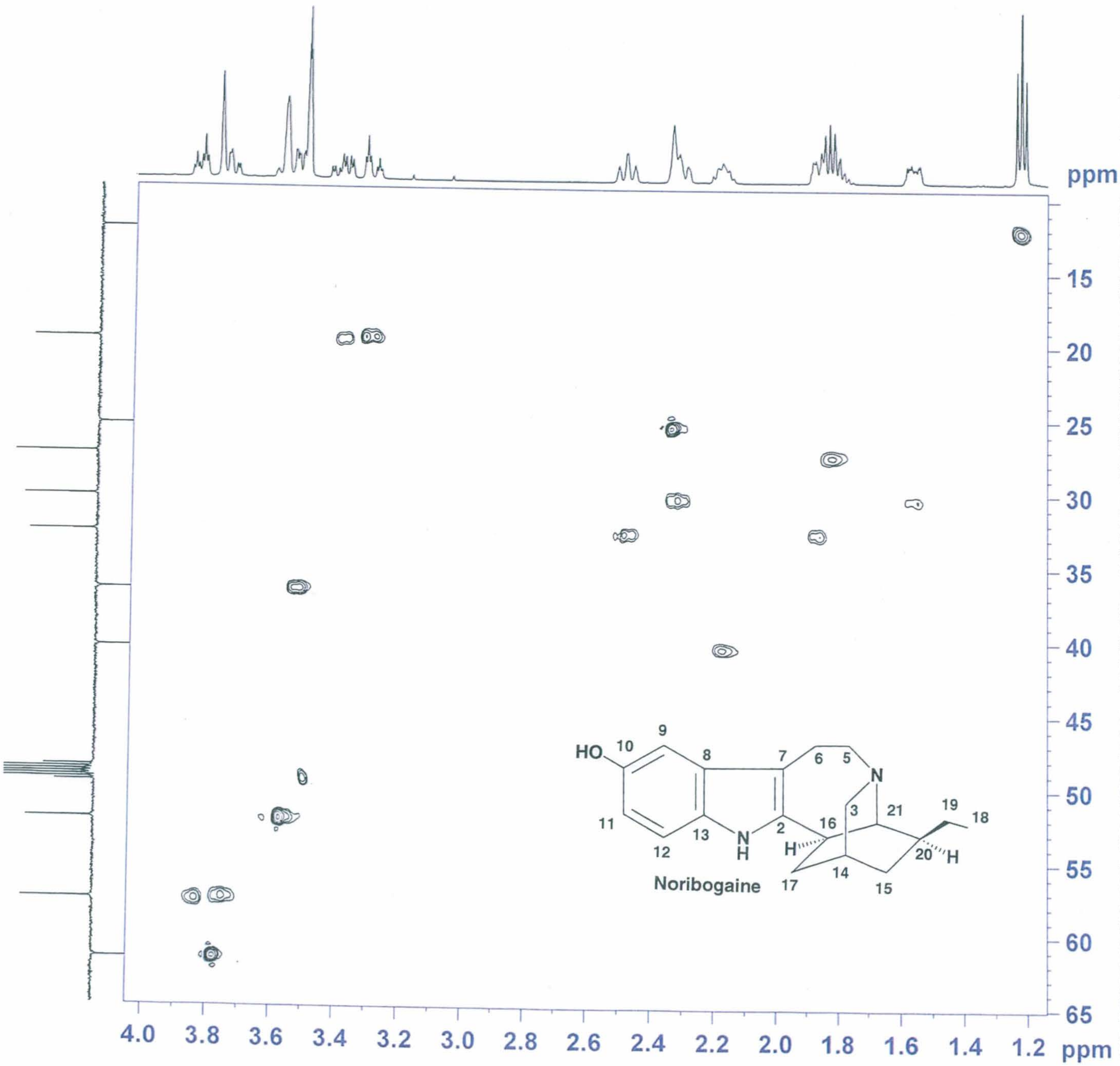

NAME	zhp297	
EXPNO	5	
PROCNO	1	
Date_	20110504	
Time	21.12	
INSTRUM	spect	
PROBHD	5 mm TXI 1H/D-	
PULPROG	hsqcetgpsi2	
TD	1024	
SOLVENT	MeOD	
NS	4	
DS	16	
SWH	3623.188	Hz
FIDRES	3.538270	Hz
AQ	0.1415000	sec
RG	18390.4	
DW	138.000	usec
DE	6.00	usec
TE	301.2	K
CNST2	145.0000000	
d0	0.00000300	sec
D1	1.43548799	sec
d4	0.00172414	sec
d11	0.03000000	sec
d13	0.00000400	sec
D16	0.00020000	sec
D24	0.00086207	sec
DELTA	0.00127330	sec
DELTA1	0.00120800	sec
DELTA2	0.00006207	sec
DELTA3	0.00052414	sec
IN0	0.00002400	sec
ST1CNT	0	
ZGOPTNS		
======== CHANNEL f1 ========		
NUC1	1H	
P1	8.65	usec
p2	17.30	usec
P28	0.10	usec
PL1	-2.00	dB
SFO1	500.1320027	MHz
======== CHANNEL f2 ========		
CPDPRG2	garp	
NUC2	13C	
P3	11.25	usec
p4	22.50	usec
PCPD2	75.00	usec
PL2	-5.50	dB
PL12	10.98	dB
SFO2	125.7672177	MHz
====== GRADIENT CHANNEL =====		
GPNAM1	SINE.100	
GPNAM2	SINE.100	
GPNAM3	SINE.100	
GPNAM4	SINE.100	
GPZ1	80.00	%
GPZ2	20.10	%
GPZ3	11.00	%
GPZ4	-5.00	%
P16	1000.00	usec
P19	600.00	usec
ND0	2	
TD	256	
SFO1	125.7672	MHz
FIDRES	81.380211	Hz
SW	165.650	ppm
FnMODE	Echo-Antiecho	
SI	1024	
SF	500.1299131	MHz
WDW	QSINE	
SSB	2	
LB	0.00	Hz
GB	0	
PC	1.40	
SI	1024	
MC2	echo-antiecho	
SF	125.7575747	MHz
WDW	QSINE	
SSB	2	
LB	0.00	Hz
GB	0	

Noribogaine source C

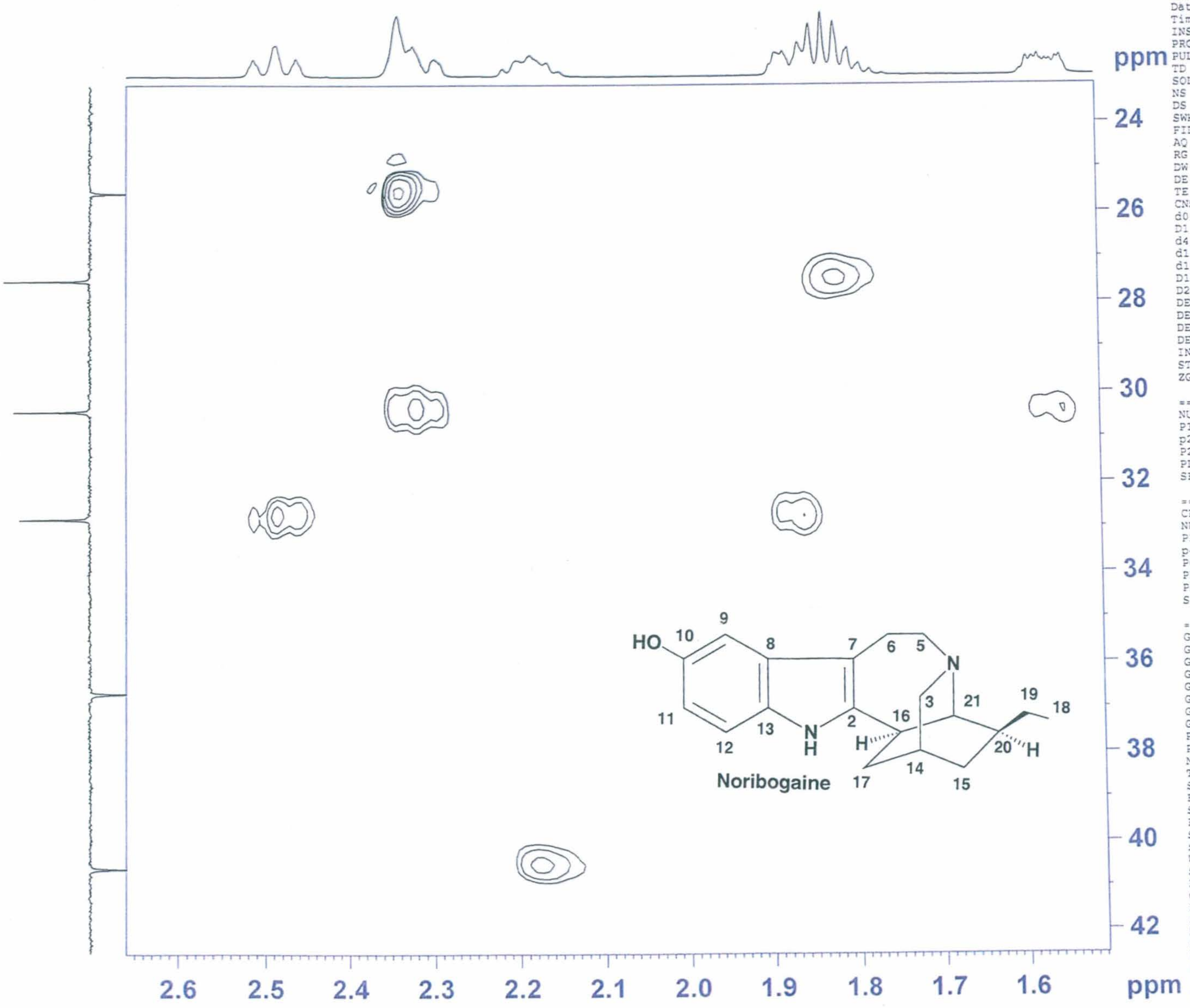

NAME	zhp297	
EXPNO	5	
PROCNO	1	
Date_	20110504	
Time	21.12	
INSTRUM	spect	
PROBHD	5 mm TXI 1H/D-	
PULPROG	hsqcetgpsi2	
TD	1024	
SOLVENT	MeOD	
NS	4	
DS	16	
SWH	3623.188	Hz
FIDRES	3.538270	Hz
AQ	0.1415000	sec
RG	18390.4	
DW	138.000	usec
DE	6.00	usec
TE	301.2	K
CNST2	145.0000000	
d0	0.00000300	sec
D1	1.43548799	sec
d4	0.00172414	sec
d11	0.03000000	sec
d13	0.00000400	sec
D16	0.00020000	sec
D24	0.00086207	sec
DELTA	0.00127330	sec
DELTA1	0.00120800	sec
DELTA2	0.00006207	sec
DELTA3	0.00052414	sec
IN0	0.00002400	sec
ST1CNT	0	
ZGOPTNS		
======== CHANNEL f1 ========		
NUC1	1H	
P1	8.65	usec
p2	17.30	usec
P28	0.10	usec
PL1	-2.00	dB
SFO1	500.1320027	MHz
======== CHANNEL f2 ========		
CPDPRG2	garp	
NUC2	13C	
P3	11.25	usec
p4	22.50	usec
PCPD2	75.00	usec
PL2	-5.50	dB
PL12	10.98	dB
SFO2	125.7672177	MHz
====== GRADIENT CHANNEL =====		
GPNAM1	SINE.100	
GPNAM2	SINE.100	
GPNAM3	SINE.100	
GPNAM4	SINE.100	
GPZ1	80.00	%
GPZ2	20.10	%
GPZ3	11.00	%
GPZ4	-5.00	%
P16	1000.00	usec
P19	600.00	usec
ND0	2	
TD	256	
SFO1	125.7672	MHz
FIDRES	81.380211	Hz
SW	165.650	ppm
FnMODE	Echo-Antiecho	
SI	1024	
SF	500.1299131	MHz
WDW	QSINE	
SSB	2	
LB	0.00	Hz
GB	0	
PC	1.40	
SI	1024	
MC2	echo-antiecho	
SF	125.7575747	MHz
WDW	QSINE	
SSB	2	
LB	0.00	Hz
GB	0	

Noribogaine source C

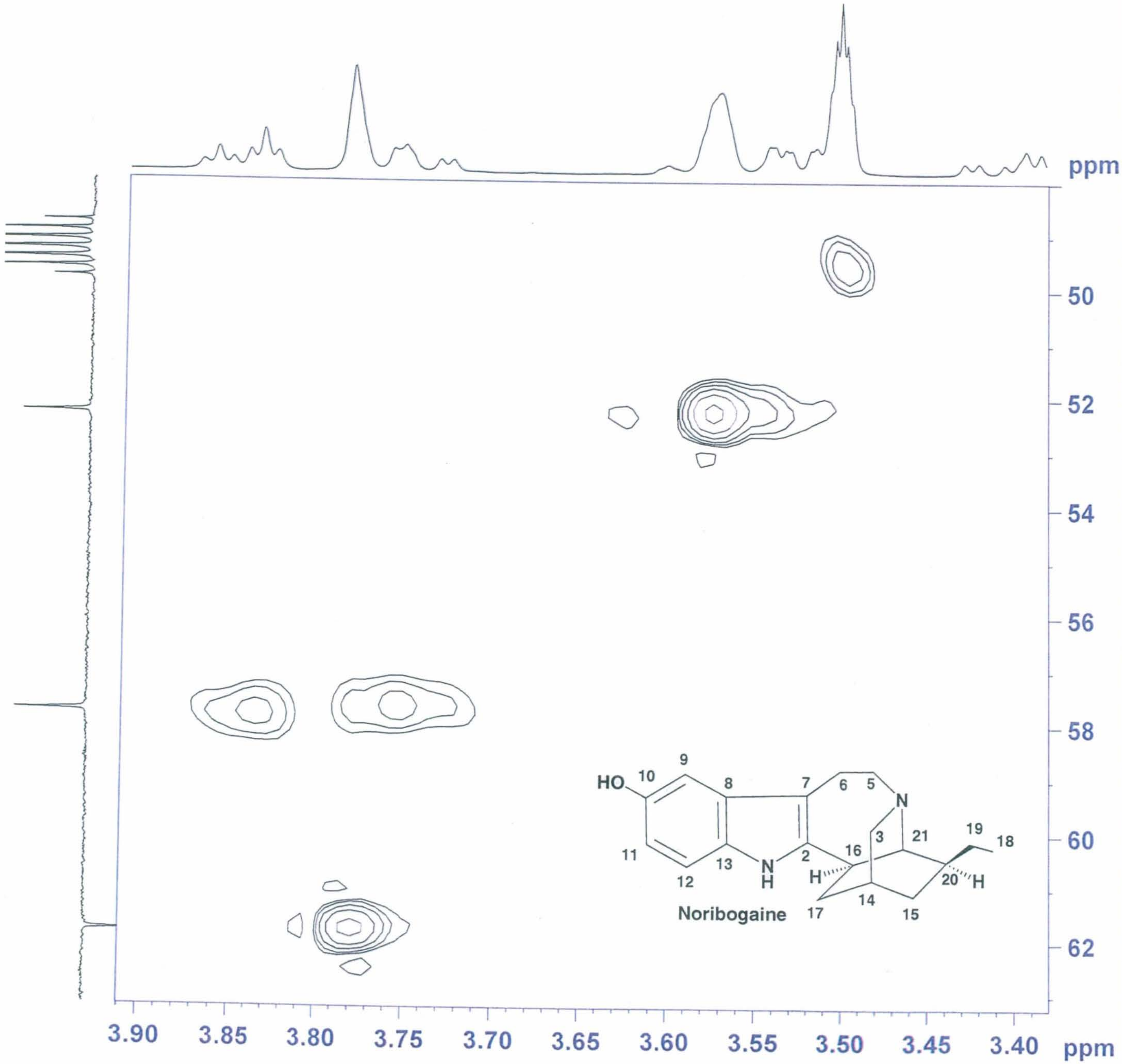

BRUKER

NAME	zhp297	
EXPNO	5	
PROCNO	1	
Date_	20110504	
Time	21.12	
INSTRUM	spect	
PROBHD	5 mm TXI 1H/D-	
PULPROG	hsqcetgpsi2	
TD	1024	
SOLVENT	MeOD	
NS	4	
DS	16	
SWH	3623.188	Hz
FIDRES	3.538270	Hz
AQ	0.1415000	sec
RG	18390.4	
DW	138.000	usec
DE	6.00	usec
TE	301.2	K
CNST2	145.0000000	
d0	0.00000300	sec
D1	1.43548799	sec
d4	0.00172414	sec
d11	0.03000000	sec
d13	0.00000400	sec
D16	0.00020000	sec
D24	0.00086207	sec
DELTA	0.00127330	sec
DELTA1	0.00120800	sec
DELTA2	0.00006207	sec
DELTA3	0.00052414	sec
IN0	0.00002400	sec
ST1CNT	0	
ZGOPTNS		
======== CHANNEL f1 ========		
NUC1	1H	
P1	8.65	usec
p2	17.30	usec
P28	0.10	usec
PL1	-2.00	dB
SFO1	500.1320027	MHz
======== CHANNEL f2 ========		
CPDPRG2	garp	
NUC2	13C	
P3	11.25	usec
p4	22.50	usec
PCPD2	75.00	usec
PL2	-5.50	dB
PL12	10.98	dB
SFO2	125.7672177	MHz
====== GRADIENT CHANNEL =====		
GPNAM1	SINE.100	
GPNAM2	SINE.100	
GPNAM3	SINE.100	
GPNAM4	SINE.100	
GPZ1	80.00	%
GPZ2	20.10	%
GPZ3	11.00	%
GPZ4	-5.00	%
P16	1000.00	usec
P19	600.00	usec
ND0	2	
TD	256	
SFO1	125.7672	MHz
FIDRES	81.380211	Hz
SW	165.650	ppm
FnMODE	Echo-Antiecho	
SI	1024	
SF	500.1299131	MHz
WDW	QSINE	
SSB	2	
LB	0.00	Hz
GB	0	
PC	1.40	
SI	1024	
MC2	echo-antiecho	
SF	125.7575747	MHz
WDW	QSINE	
SSB	2	
LB	0.00	Hz
GB	0	

Noribogaine source C

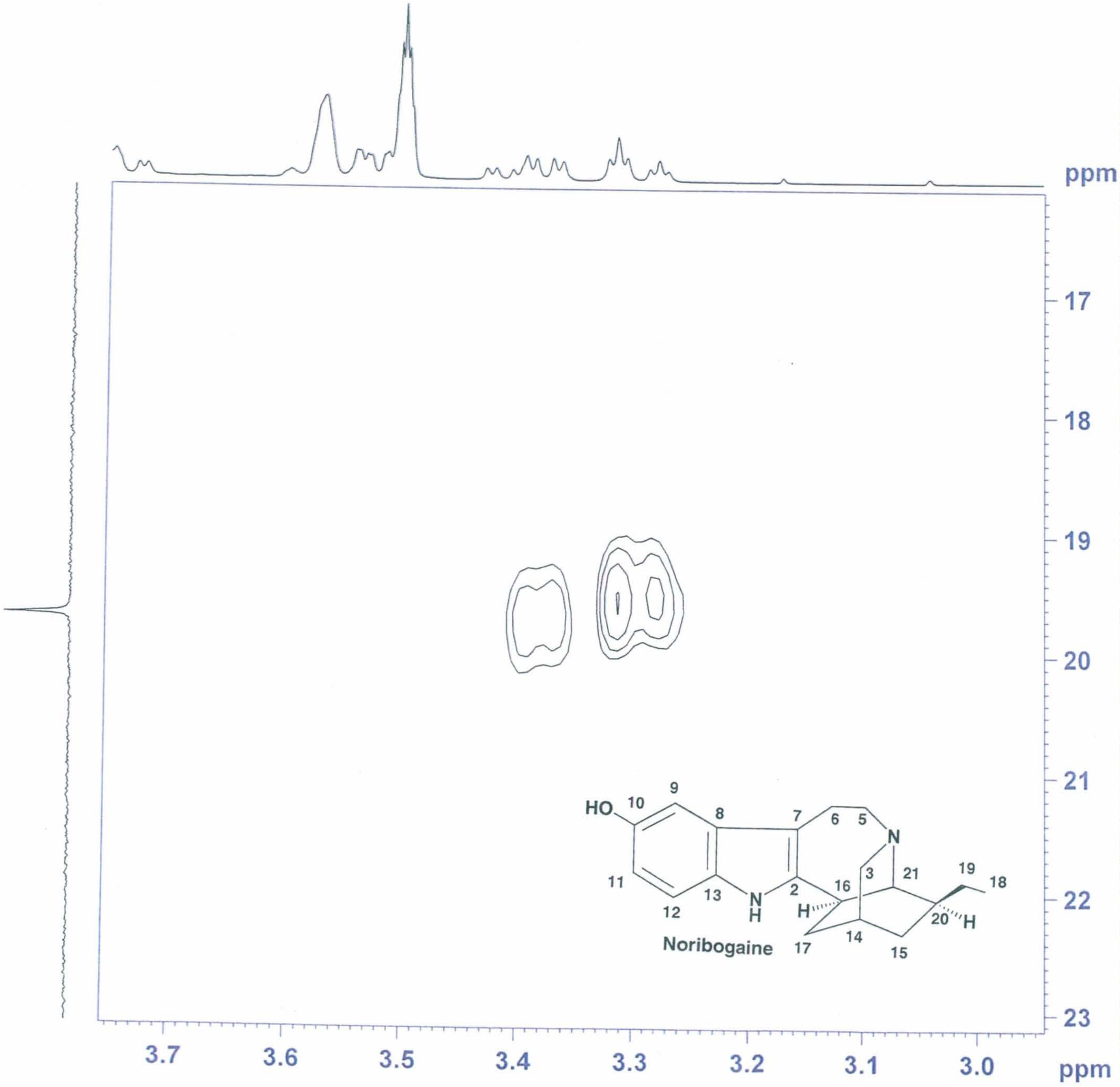


```
NAME              zhp297
EXPNO                  5
PROCNO                 1
Date_           20110504
Time               21.12
INSTRUM            spect
PROBHD    5 mm TXI 1H/D-
PULPROG      hsqcetgpsi2
TD                  1024
SOLVENT             MeOD
NS                     4
DS                    16
SWH             3623.188 Hz
FIDRES          3.538270 Hz
AQ             0.1415000 sec
RG               18390.4
DW               138.000 usec
DE                  6.00 usec
TE                 301.2 K
CNST2        145.0000000
d0            0.00000300 sec
D1            1.43548799 sec
d4            0.00172414 sec
d11           0.03000000 sec
d13           0.00000400 sec
D16           0.00020000 sec
D24           0.00086207 sec
DELTA         0.00127330 sec
DELTA1        0.00120800 sec
DELTA2        0.00006207 sec
DELTA3        0.00052414 sec
IN0           0.00002400 sec
ST1CNT                 0
ZGOPTNS

======== CHANNEL f1 ========
NUC1                  1H
P1                  8.65 usec
p2                 17.30 usec
P28                 0.10 usec
PL1                -2.00 dB
SFO1         500.1320027 MHz

======== CHANNEL f2 ========
CPDPRG2             garp
NUC2                 13C
P3                 11.25 usec
p4                 22.50 usec
PCPD2              75.00 usec
PL2                -5.50 dB
PL12               10.98 dB
SFO2         125.7672177 MHz

====== GRADIENT CHANNEL =====
GPNAM1          SINE.100
GPNAM2          SINE.100
GPNAM3          SINE.100
GPNAM4          SINE.100
GPZ1               80.00 %
GPZ2               20.10 %
GPZ3               11.00 %
GPZ4               -5.00 %
P16              1000.00 usec
P19               600.00 usec
ND0                    2
TD                   256
SFO1            125.7672 MHz
FIDRES         81.380211 Hz
SW               165.650 ppm
FnMODE     Echo-Antiecho
SI                  1024
SF           500.1299131 MHz
WDW                QSINE
SSB                    2
LB                  0.00 Hz
GB                     0
PC                  1.40
SI                  1024
MC2        echo-antiecho
SF           125.7575747 MHz
WDW                QSINE
SSB                    2
LB                  0.00 Hz
GB                     0
```

Noribogaine source C

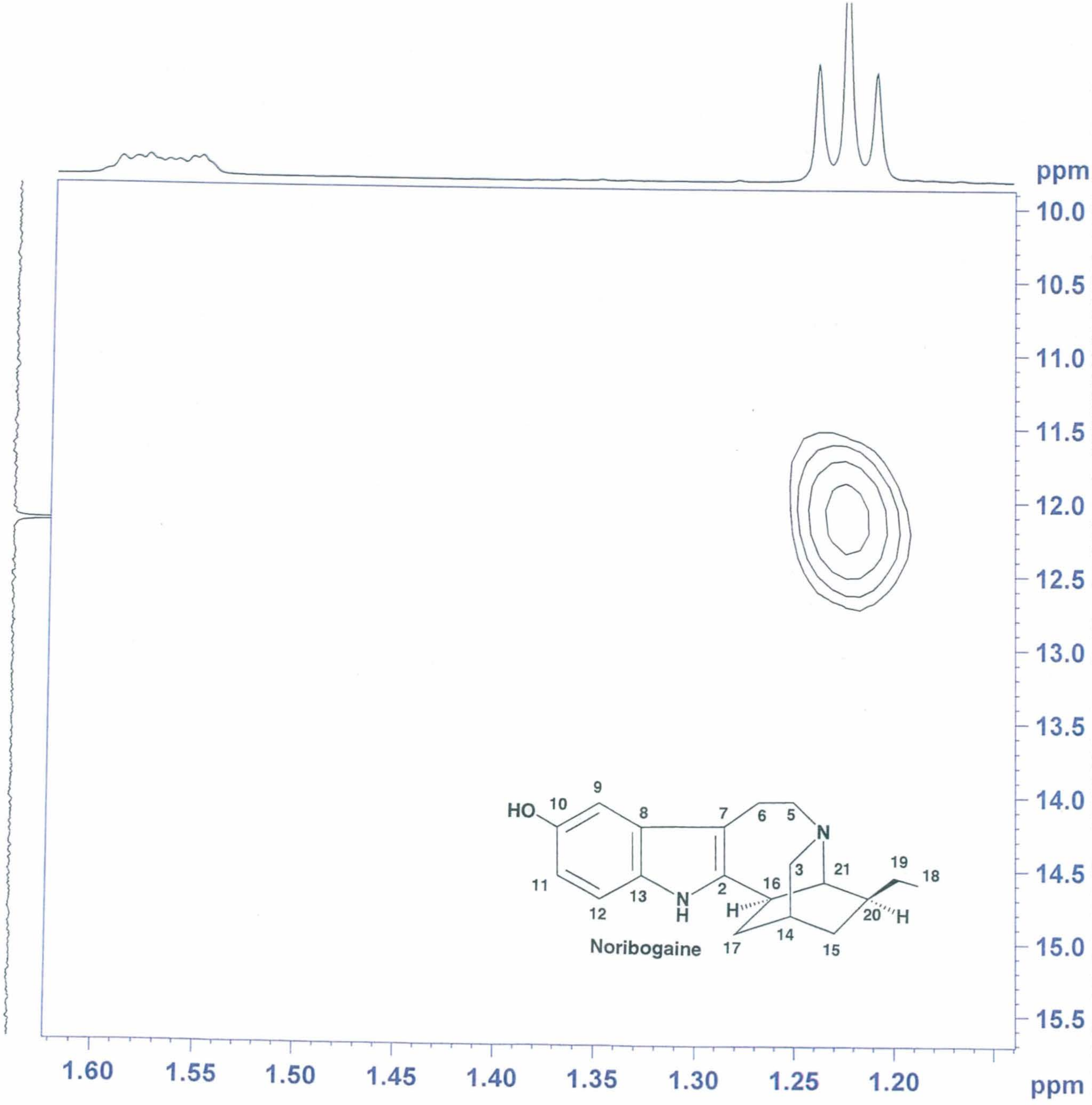

NAME	zhp297	
EXPNO	5	
PROCNO	1	
Date_	20110504	
Time	21.12	
INSTRUM	spect	
PROBHD	5 mm TXI 1H/D-	
PULPROG	hsqcetgpsi2	
TD	1024	
SOLVENT	MeOD	
NS	4	
DS	16	
SWH	3623.188	Hz
FIDRES	3.538270	Hz
AQ	0.1415000	sec
RG	18390.4	
DW	138.000	usec
DE	6.00	usec
TE	301.2	K
CNST2	145.0000000	
d0	0.00000300	sec
D1	1.43548799	sec
d4	0.00172414	sec
d11	0.03000000	sec
d13	0.00000400	sec
D16	0.00020000	sec
D24	0.00086207	sec
DELTA	0.00127330	sec
DELTA1	0.00120800	sec
DELTA2	0.00006207	sec
DELTA3	0.00052414	sec
IN0	0.00002400	sec
ST1CNT	0	
ZGOPTNS		
======== CHANNEL f1 ========		
NUC1	1H	
P1	8.65	usec
p2	17.30	usec
P28	0.10	usec
PL1	-2.00	dB
SFO1	500.1320027	MHz
======== CHANNEL f2 ========		
CPDPRG2	garp	
NUC2	13C	
P3	11.25	usec
p4	22.50	usec
PCPD2	75.00	usec
PL2	-5.50	dB
PL12	10.98	dB
SFO2	125.7672177	MHz
====== GRADIENT CHANNEL =====		
GPNAM1	SINE.100	
GPNAM2	SINE.100	
GPNAM3	SINE.100	
GPNAM4	SINE.100	
GPZ1	80.00	%
GPZ2	20.10	%
GPZ3	11.00	%
GPZ4	-5.00	%
P16	1000.00	usec
P19	600.00	usec
ND0	2	
TD	256	
SFO1	125.7672	MHz
FIDRES	81.380211	Hz
SW	165.650	ppm
FnMODE	Echo-Antiecho	
SI	1024	
SF	500.1299131	MHz
WDW	QSINE	
SSB	2	
LB	0.00	Hz
GB	0	
PC	1.40	
SI	1024	
MC2	echo-antiecho	
SF	125.7575747	MHz
WDW	QSINE	
SSB	2	
LB	0.00	Hz
GB	0	

Noribogaine source C

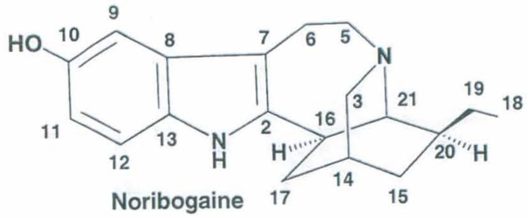

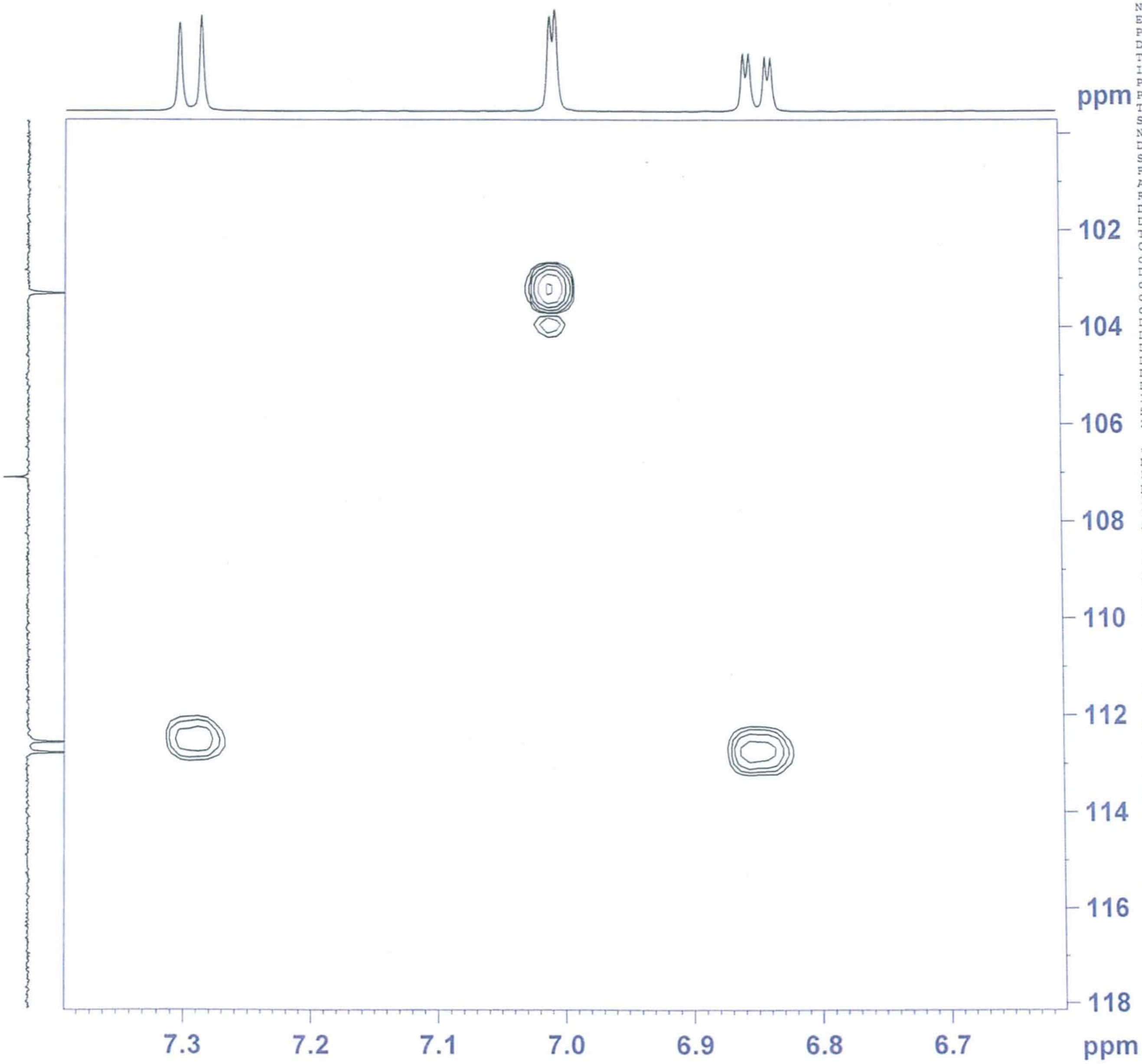

NAME	zhp297	
EXPNO	5	
PROCNO	1	
Date_	20110504	
Time	21.12	
INSTRUM	spect	
PROBHD	5 mm TXI 1H/D-	
PULPROG	hsqcetgpsi2	
TD	1024	
SOLVENT	MeOD	
NS	4	
DS	16	
SWH	3623.188	Hz
FIDRES	3.538270	Hz
AQ	0.1415000	sec
RG	18390.4	
DW	138.000	usec
DE	6.00	usec
TE	301.2	K
CNST2	145.0000000	
d0	0.00000300	sec
D1	1.43548799	sec
d4	0.00172414	sec
d11	0.03000000	sec
d13	0.00000400	sec
D16	0.00020000	sec
D24	0.00086207	sec
DELTA	0.00127330	sec
DELTA1	0.00120800	sec
DELTA2	0.00006207	sec
DELTA3	0.00052414	sec
IN0	0.00002400	sec
ST1CNT	0	
ZGOPTNS		
======== CHANNEL f1 ========		
NUC1	1H	
P1	8.65	usec
p2	17.30	usec
P28	0.10	usec
PL1	-2.00	dB
SFO1	500.1320027	MHz
======== CHANNEL f2 ========		
CPDPRG2	garp	
NUC2	13C	
P3	11.25	usec
p4	22.50	usec
PCPD2	75.00	usec
PL2	-5.50	dB
PL12	10.98	dB
SFO2	125.7672177	MHz
====== GRADIENT CHANNEL =====		
GPNAM1	SINE.100	
GPNAM2	SINE.100	
GPNAM3	SINE.100	
GPNAM4	SINE.100	
GPZ1	80.00	%
GPZ2	20.10	%
GPZ3	11.00	%
GPZ4	-5.00	%
P16	1000.00	usec
P19	600.00	usec
ND0	2	
TD	256	
SFO1	125.7672	MHz
FIDRES	81.380211	Hz
SW	165.650	ppm
FnMODE	Echo-Antiecho	
SI	1024	
SF	500.1299131	MHz
WDW	QSINE	
SSB	2	
LB	0.00	Hz
GB	0	
PC	1.40	
SI	1024	
MC2	echo-antiecho	
SF	125.7575747	MHz
WDW	QSINE	
SSB	2	
LB	0.00	Hz
GB	0	

Noribogaine source C

BB1

**Sample Name**	martin1297	**Position**	Vial 2	**Instrument Name**	Instrument 1	**User Name**	
**Inj Vol**	5	**InjPosition**		**SampleType**	Sample	**IRM Calibration Status**	All Ions Missed
**Data Filename**	blank1-r002974.d	**ACQ Method**	hplconly3hcoohacn.m	**Comment**		**Acquired Time**	5/4/2011 4:33:21 PM

peak 297

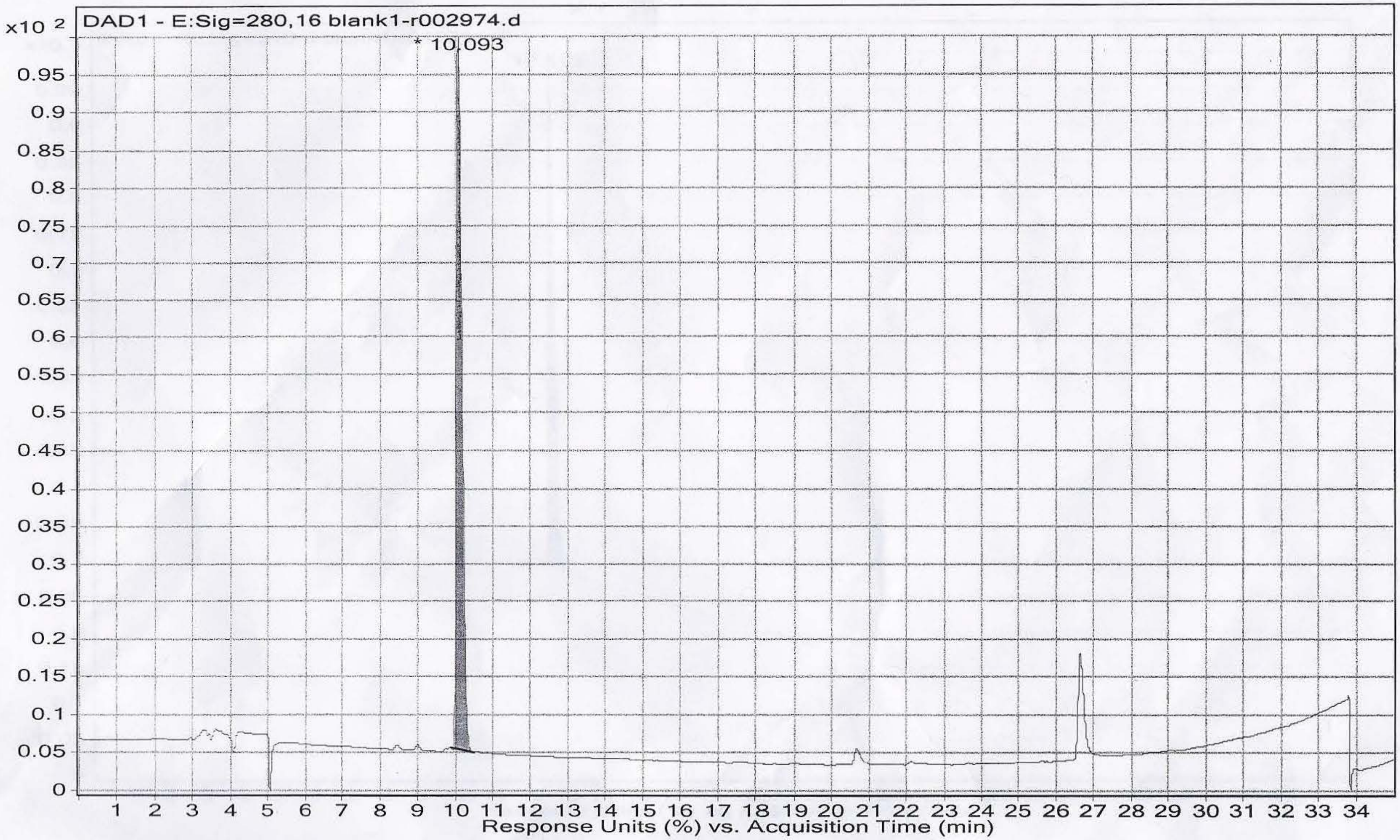

NOR - Ibogaine

(noribogaine)

Noribogaine source C

**Sample Name**	martin1311	**Position**	Vial 4	**Instrument Name**	Instrument 1	**User Name**	
**Inj Vol**	1	**InjPosition**		**SampleType**	Sample	**IRM Calibration Status**	All Ions Missed
**Data Filename**	blank1-r010311.d	**ACQ Method**	hplconly3hcoohacn.m	**Comment**		**Acquired Time**	5/4/2011 8:11:59 PM

M.W. 311.

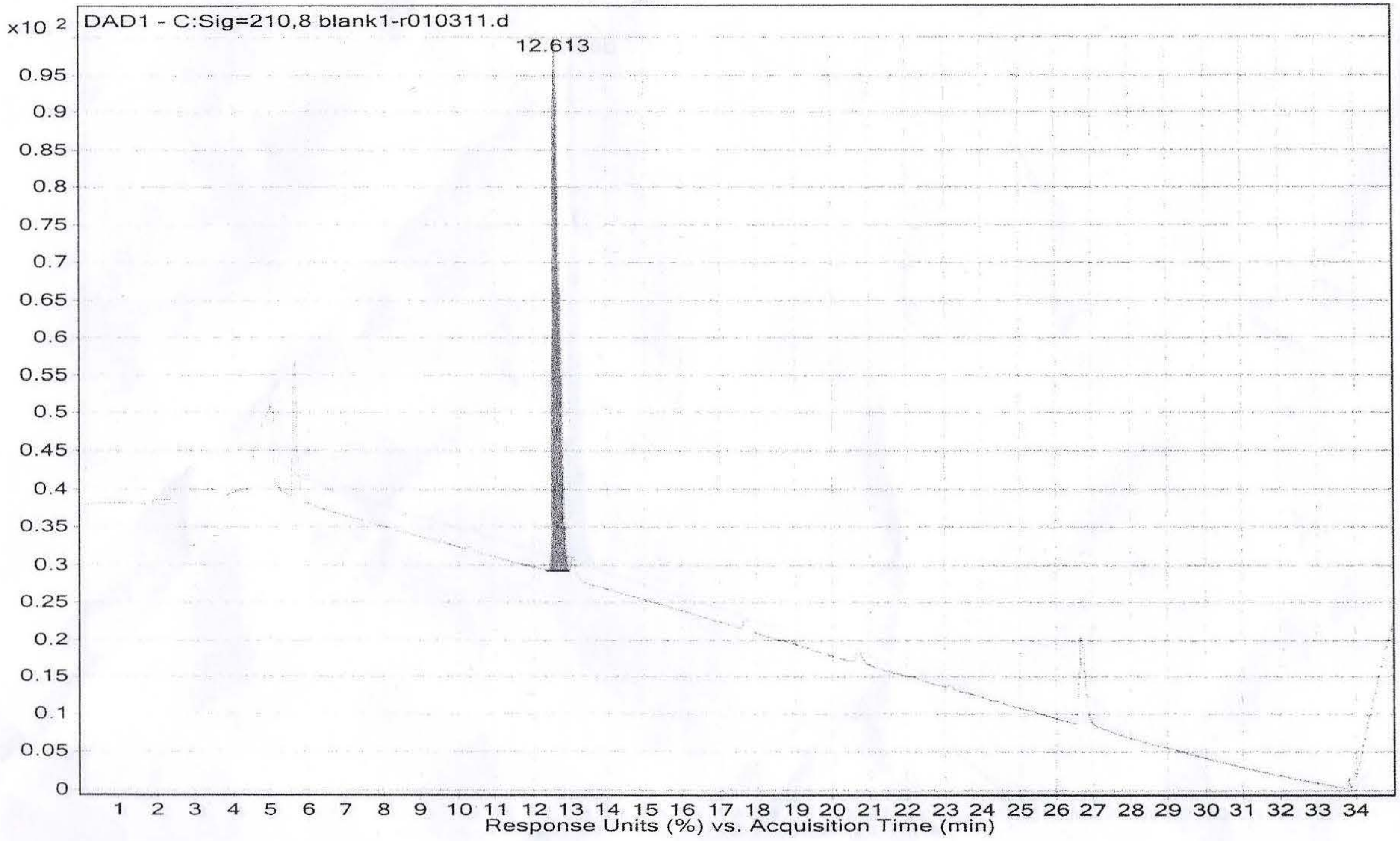

Ibogaine

(ibogaine)

Noribogaine source C

Sample Name	martin1281	Position	Vial 3	Instrument Name	Instrument 1	User Name	
Inj Vol	2	InjPosition		SampleType	Sample	IRM Calibration Status	All Ions Missed
Data Filename	blank1-r007281.d	ACQ Method	hplconly3hcoohacn.m	Comment		Acquired Time	5/4/2011 6:22:33 PM

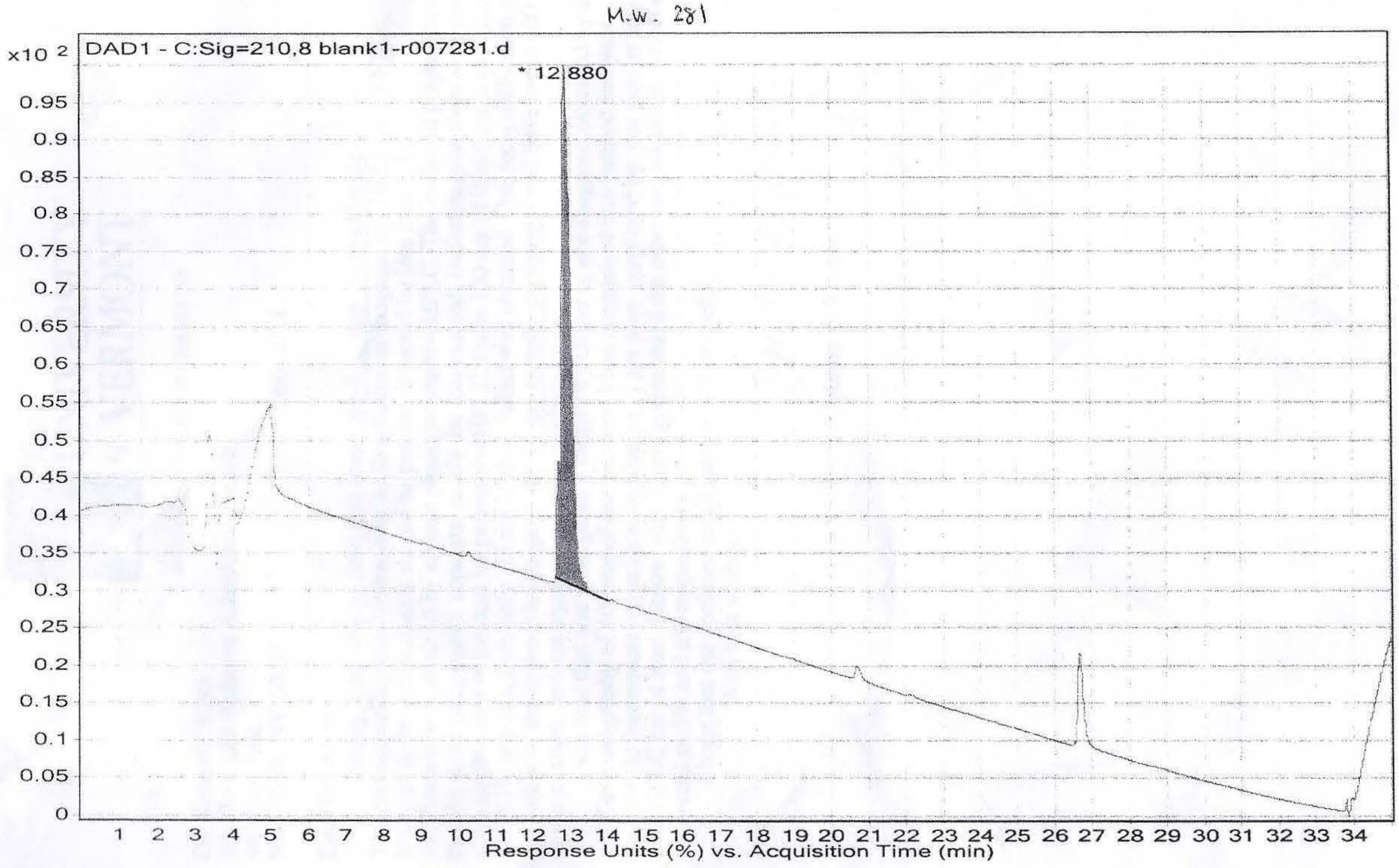

Ibogamine

(ibogamine)

**Sample Name**	martin1blnak1	**Position**	Vial 1	**Instrument Name**	Instrument 1	**User Name**	
**Inj Vol**	1	**InjPosition**		**SampleType**	Sample	**IRM Calibration Status**	All Ions Missed
**Data Filename**	blank1-r005.d	**ACQ Method**	hplconly3hcoohacn.m	**Comment**		**Acquired Time**	5/4/2011 5:09:46 PM

Blank sample

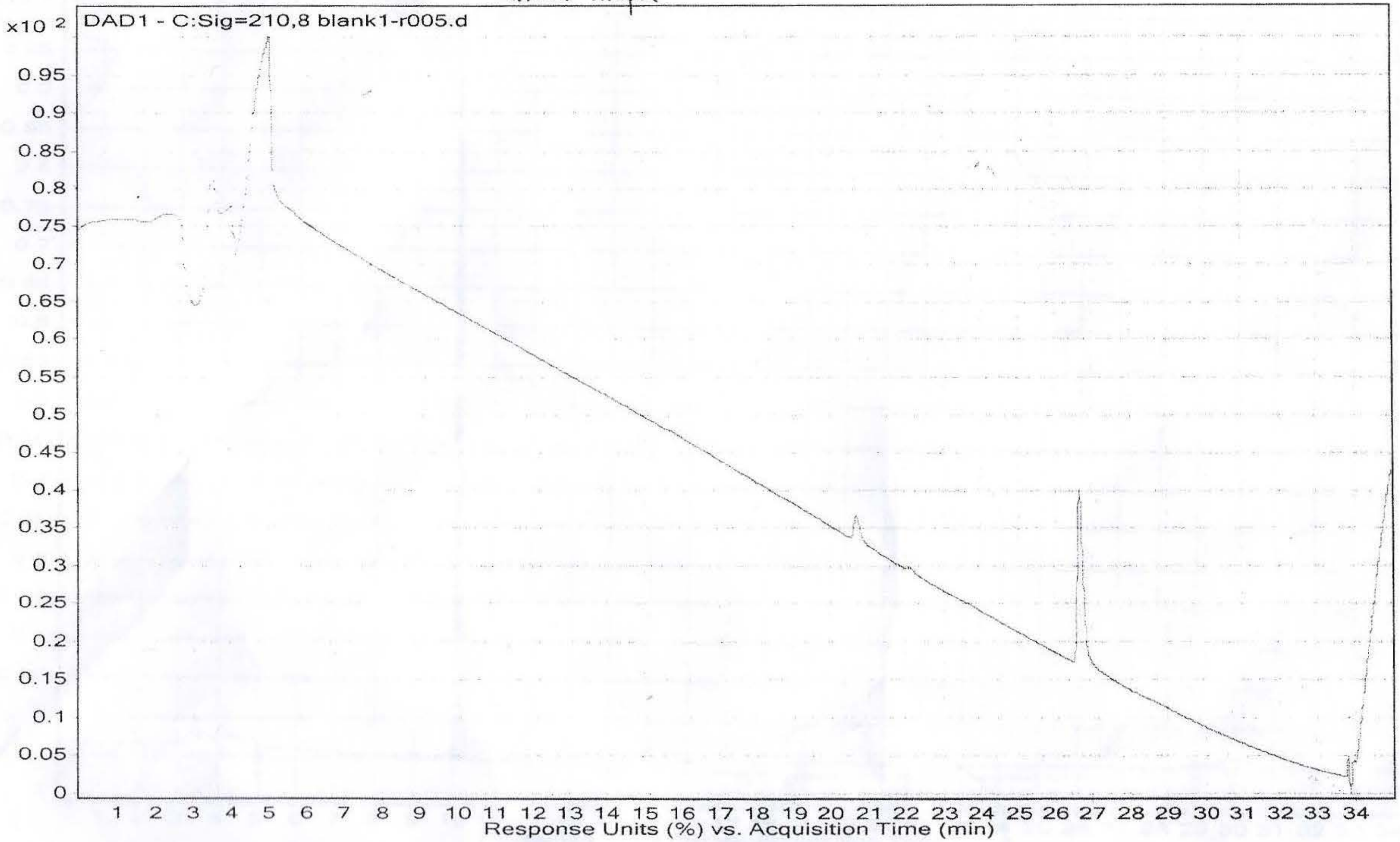

(blank sample)

Noribogaine source D

Analyst Version: 1.5

TIC of +Q1: from Sample 8 (MK 500 NORIB 03) of MK110831.wiff (Heated Nebulizer) Max. 1.8e8 cps.

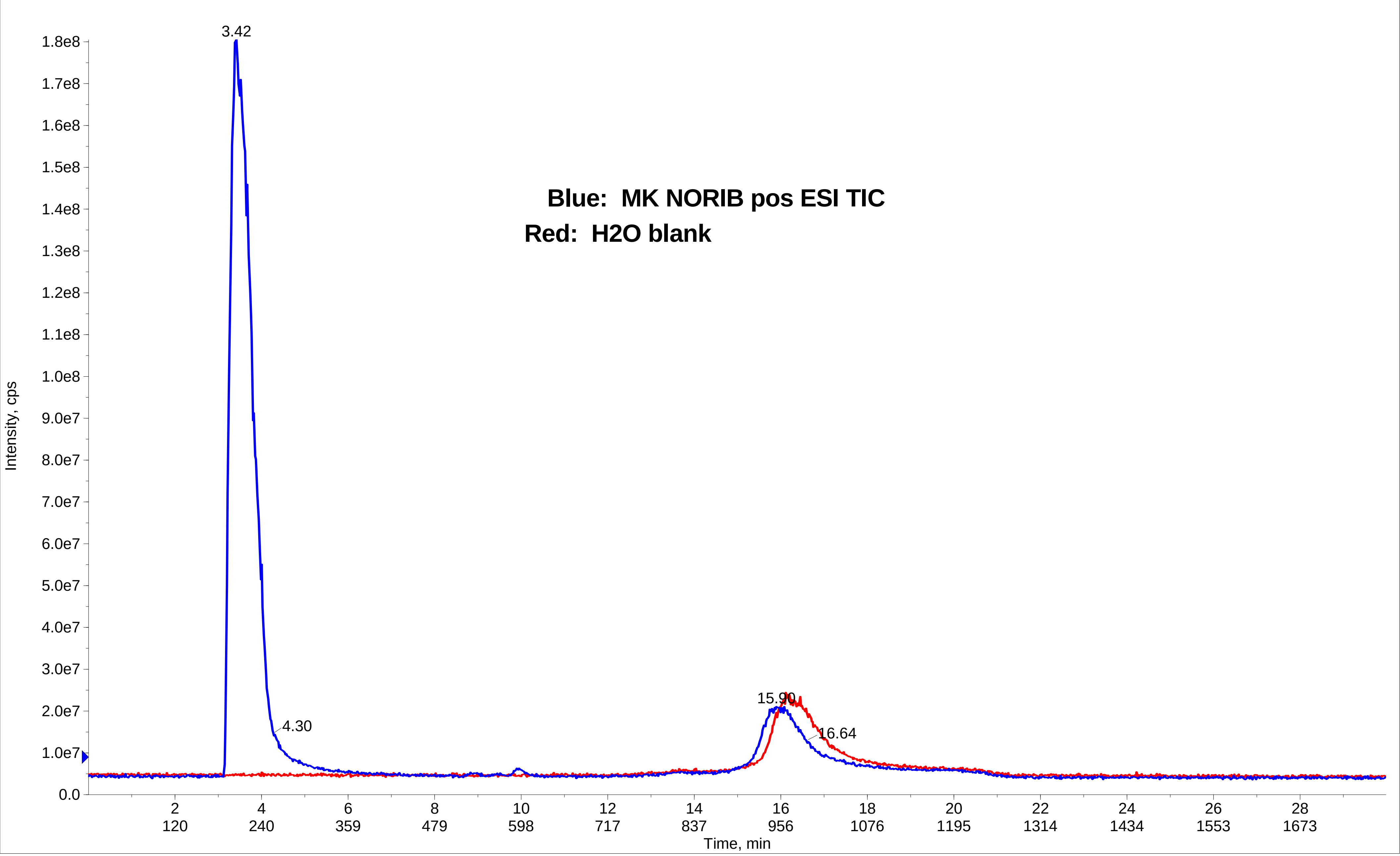

Acq. Date: Wednesday, August 31, 2011
Acq. File: MK110831.wiff

+Q1: 3.166 to 3.953 min from Sample 8 (MK 500 NORIB 03) of MK110831.wiff (Heated Nebulizer), subtract... Max. 5.0e6 cps.

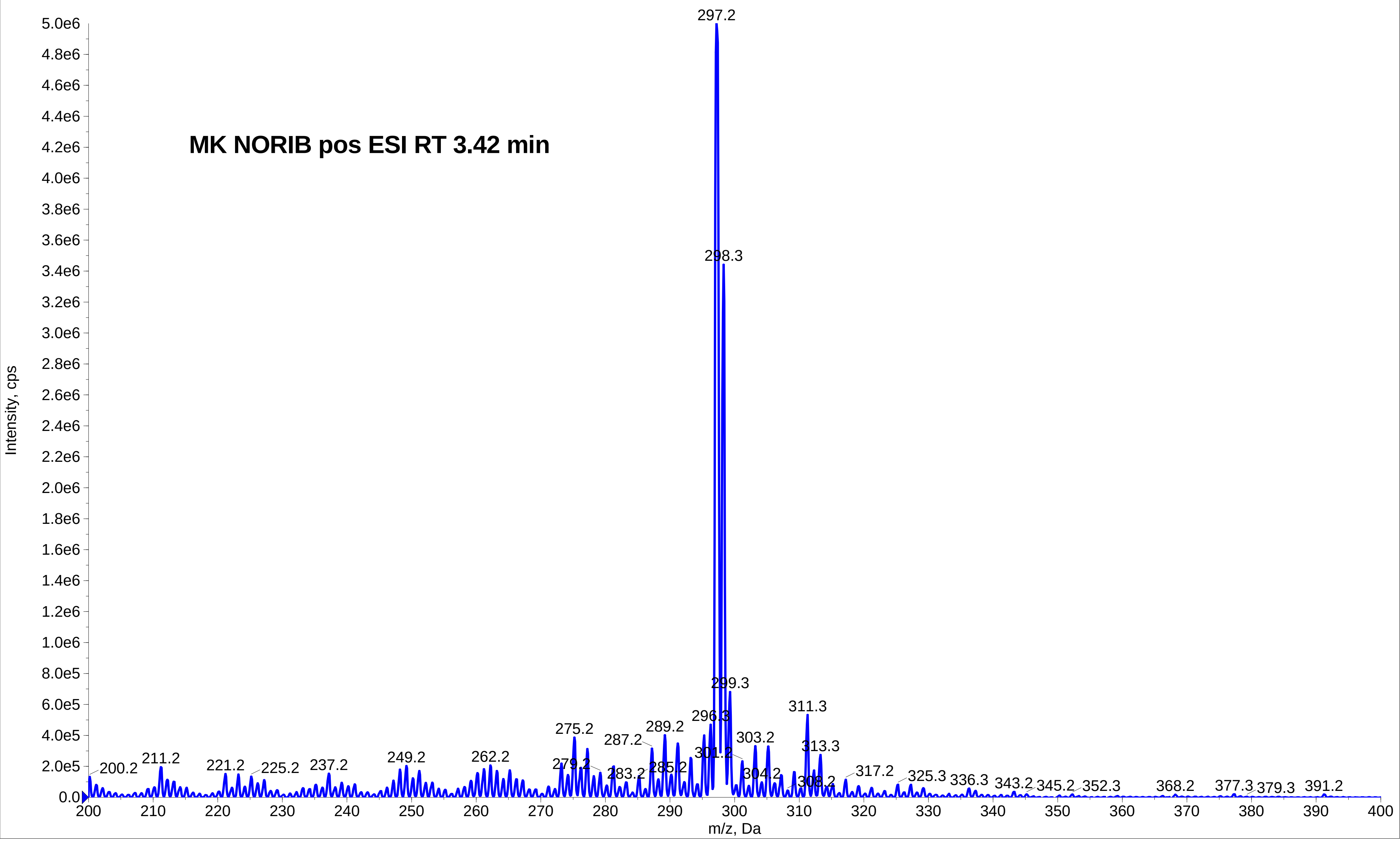

Acq. Date: Wednesday, August 31, 2011
Acq. File: MK110831.wiff

Noribogaine source D

Workstation: 4000QTRAP
Operator: ABI4000

Analyst Version: 1.5

HPLC/MS analysis for the tail end of the noribogaine peak graph for source D

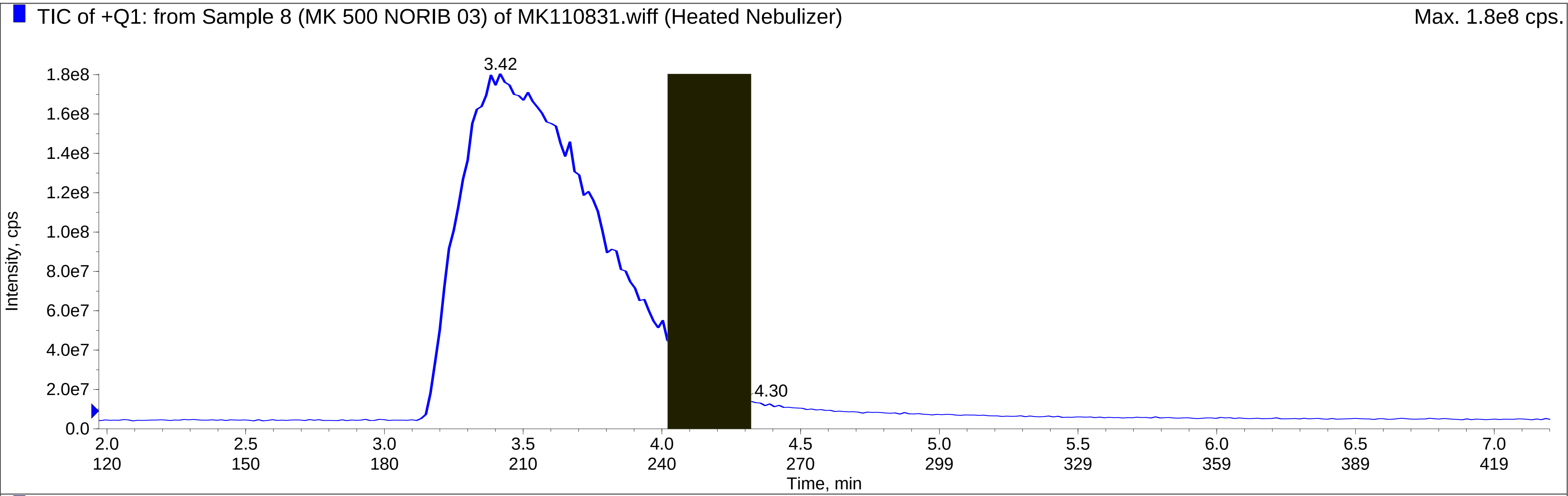

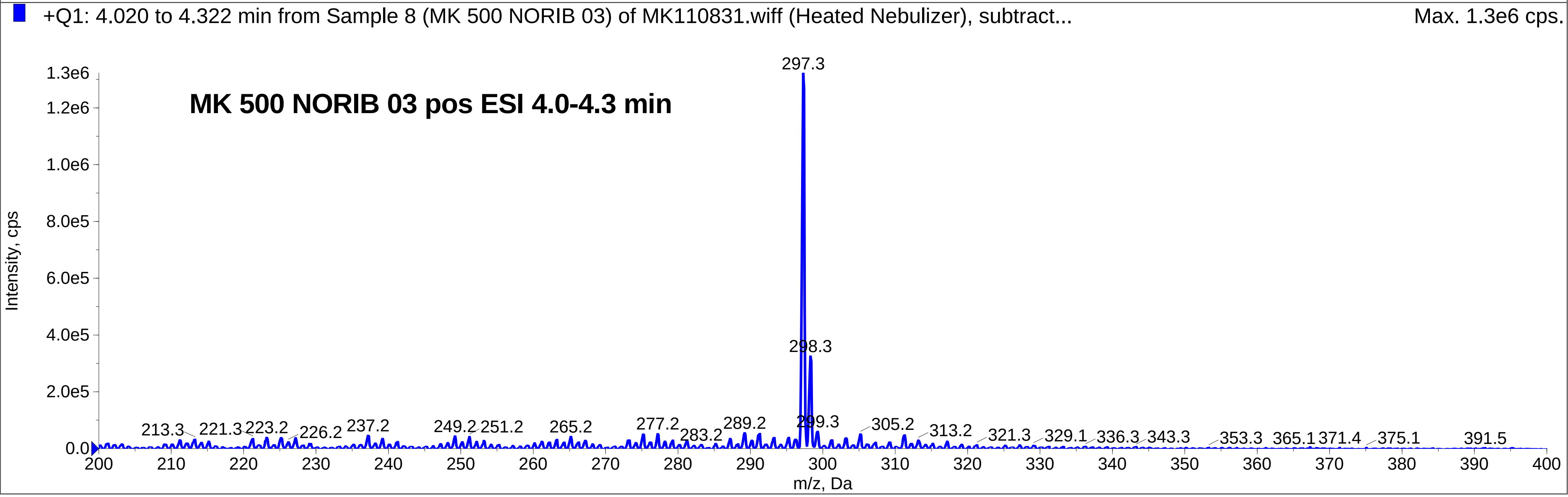

Acq. Date: Wednesday, August 31, 2011
Acq. File: MK110831.wiff

2809 Gemini Court
Champaign, IL 61822

Tel: (217) 359-1626
Fax: (217) 359-2567
info@obires.com

OBITER RESEARCH, LLC

Fine Kilo-Scale Chemicals | Unique Products | Contract Synthesis | Custom Manufacturing

CERTIFICATE OF ANALYSIS

July 16, 2013

Obiter Research obtained the following results from analysis of the material described below:

COMPOUND NAME: 18-Methoxycoronaridine Hydrochloride
COMPOUND DESCRIPTION: White Powder
LOT NUMBER: OBI-220-158-1
LAST SOLVENT: CH_2Cl_2/Ether
FORMULA WEIGHT: 404.93
CHEMICAL FORMULA: $C_{22}H_{29}ClN_2O_3$
CAS NUMBER:

PROJECT/PRODUCT NAME: OBT-195

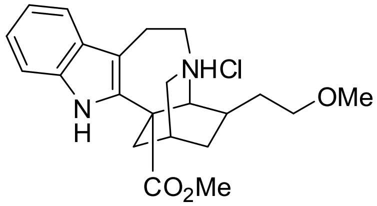

^{1}H-NMR:

Spectrometer Frequency:
Solvent: DMSO-d_6
Spectrum: Consistent with expected structure (attached)

HPLC:

Column: Phenomenex C18 150 mm x 4.6 mm
Adsorbent: Columbus 5 μ C-18, 110A
Flow Rate: 1.0 mL/min.
Solvent System: 75% ACN:25%H_2O
Injection Volume: 15μL
Temperature: 26°C
λ observed: 254 nm
Purity: > 99.0%

Retention Time: 5.423 min
(Spectrum attached)

Submitted by: ______________________ Date: ____________

Approved by: ______________________ Date: ____________

*   ATT 2^ 6   @
*   RUN #   196   DEC 23, 2010   14:58:49

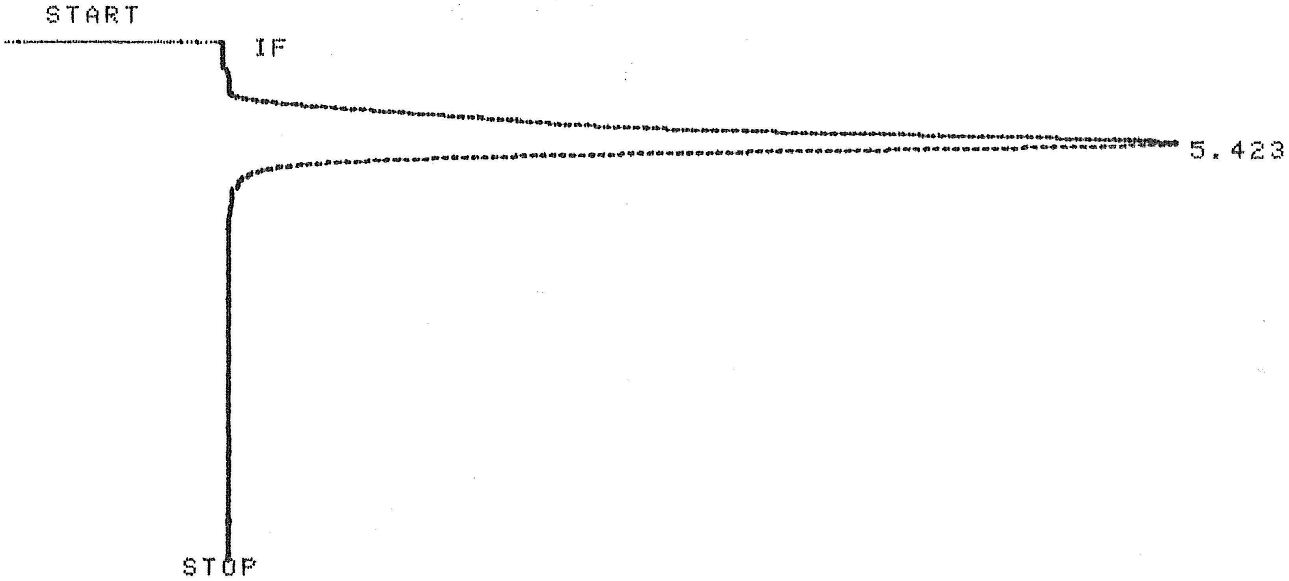

RUN#   196   DEC 23, 2010   14:58:49

AREA%

RT	AREA	TYPE	WIDTH	AREA%
5.423	31222192	BB	1.479	100.00000

TOTAL AREA=3.1222E+07
MUL FACTOR=1.0000E+00

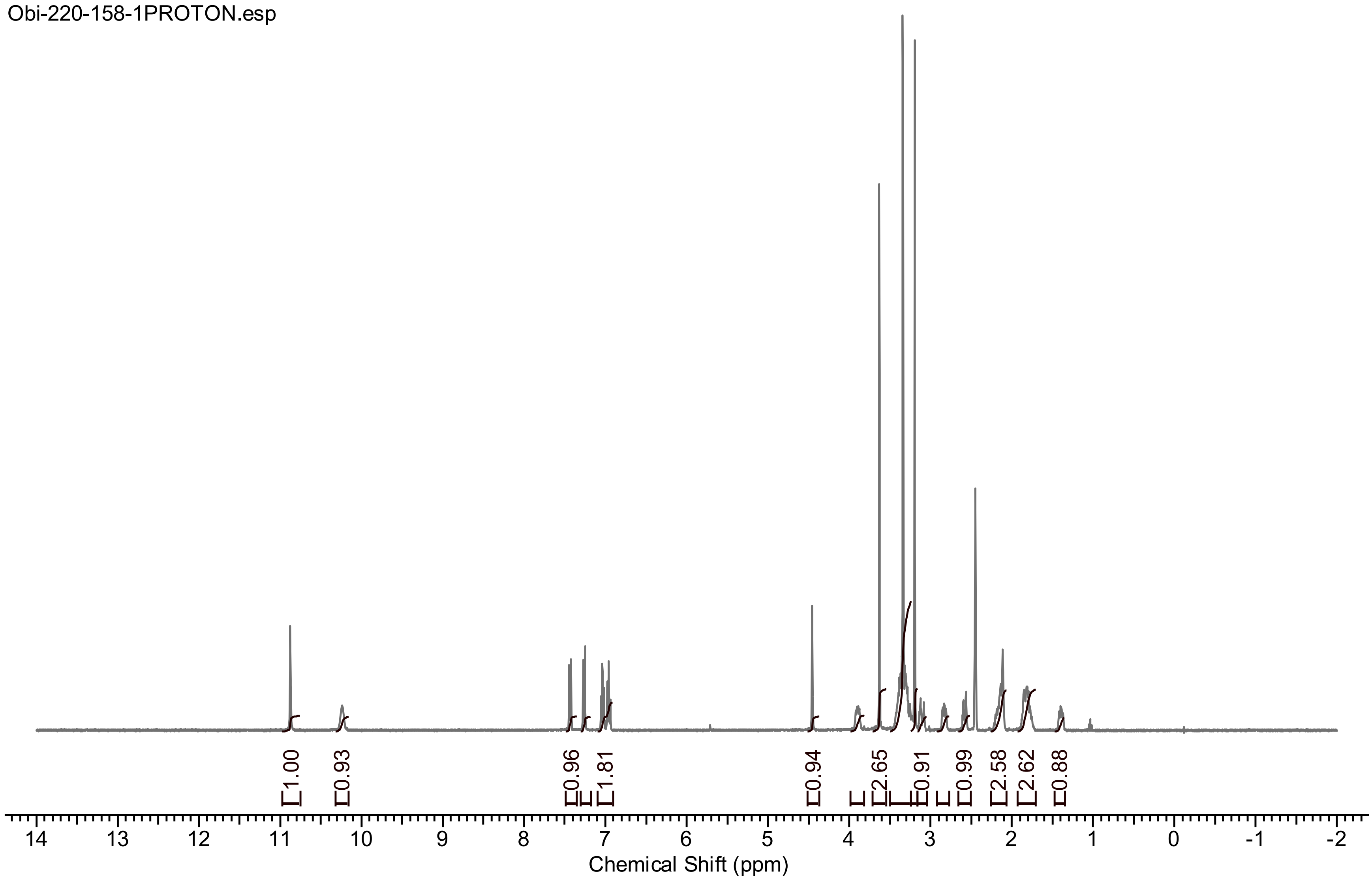
Obi-220-158-1PROTON.esp
1.00
0.93
0.96
1.81
0.94
2.65
0.91
0.99
2.58
2.62
0.88
14
13
12
11
10
9
8
7
6
5
4
3
2
1
0
-1
-2
Chemical Shift (ppm)

18-MC

Workstation: 4000QTRAP
Operator: ABI4000

Analyst Version: 1.5

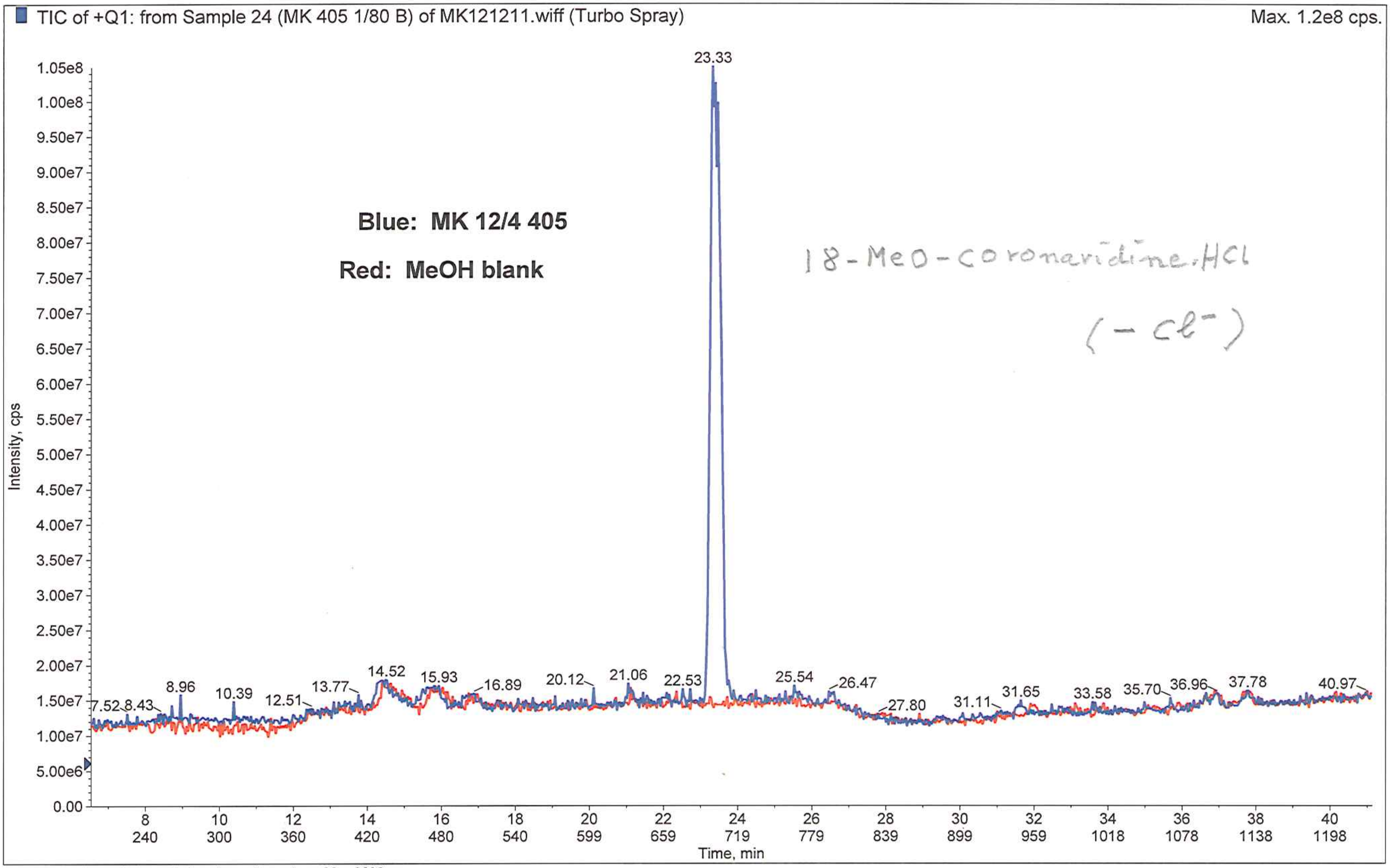

Acq. Date: Wednesday, December 12, 2012
Acq. File: MK121211.wiff

18-MC

Workstation: 4000QTRAP
Operator: ABI4000

Analyst Version: 1.5

+Q1: 23.125 to 23.727 min from Sample 24 (MK 405 1/80 B) of MK121211.wiff (Turbo Spray), subtracted (2... Max. 3.9e6 cps.

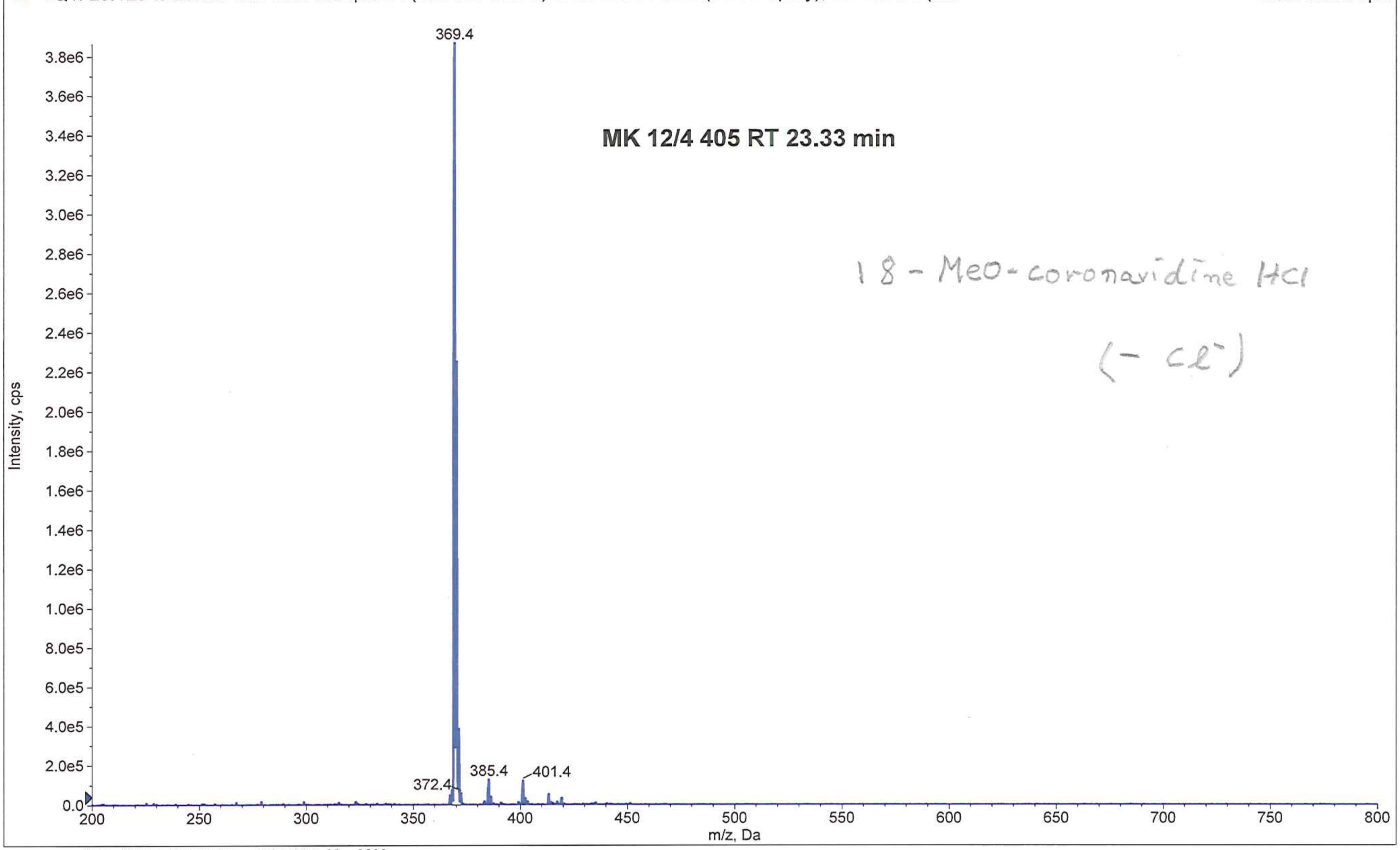

Acq. Date: Wednesday, December 12, 2012
Acq. File: MK121211.wiff
