# Supplementary material for: Effect of Iboga Alkaloids on µ-Opioid Receptor-Coupled G Protein Activation
Source: PLoS One. 2013 Oct 16;8(10):e77262. doi: 10.1371/journal.pone.0077262 (PMC3818563; doi:10.1371/journal.pone.0077262)
Supplement: File S2 — Supporting text to accompany File S1. The supporting text references the graphs/analytic data for each compound and source according to headers and page numbers indicated above in File S1. (PDF) [file pone.0077262.s002.pdf]

Supporting Information File S2

Text to accompany Supporting Information File S1

The text in this file accompanies the respective graphs/analytic data presented in Supporting Information File S1. The compounds are presented following order in both Supporting Information Files S1 and S2: ibogaine source A, ibogaine source B, noribogaine source C, noribogaine source D, and 18-MC. Within Supporting Information File S1, pages are labeled and numbered separately for each respective compound. On each page, the iboga alkaloid and source are indicated as headers at top center and the page numbers appear on the right upper corner.

Ibogaine source A:

Ibogaine HCl obtained from Slater & Frith Ltd (Wroxham Norwich, UK). Samples were analyzed on a Varian Saturn 2100T gas chromatograph-ion trap mass spectrometer with a Varian 3900 GC and CP-8400 autosampler. The mass spectrometer was operated in positive chemical ionization mode using methanol as the chemical ionization reagent gas. One microliter of sample was injected onto a Phenomenex Zebron ZB-5 (5% phenyl-95% dimethyl-polysiloxane) GC column (30m x 0.25 mm ID x 0.25 um film) with the following chromatographic conditions: injection port temp 270 °C; Initial GC temp 50 °C; Initial Time 2 min; ramp rate 30°C/min; final temp 310 °C; final time 15 min.

Pages 1-3: Overall purity of this free base sample is 95%. In this sample the first GC peak on the left is for ibogamine, the smaller between ibogamine and ibogaine may

be tabernanthine (a comparison standard was not available), the largest peak is ibogaine and the third peak is an unknown and corresponds to ibogaine minus 2H.

Ibogaine source B:

Ibogaine HCl source B was obtained from National Institute on Drug Abuse Research Resources Drug Supply Program.

Pages 1-3: Research Triangle Institute (RTI) data sheet with HPLC-MS. Overall purity of this sample is 97.2 ± 0.06% by reversed phase HPLC-MS (total area analysis).

Noribogaine Source C:

Noribogaine HCl was prepared by conversion from ibogaine source A by Kuehne lab with preparative HPLC purification at the Bornmann lab. 400 mg of ibogaine HCl (source A above) was demethylated with boron tribromide by the Archer procedure [1] at the Kuehne lab to provide 220 mg of crude product which had a reverse phase HPLC-MS composition (retention times/MW+1) of 65% noribogaine (5.6 min/297), 29% ibogaine (10.9 min/311), and 6% ibogamine (11.4 min/281).

Preparative HPLC was carried out at the Bornmann lab on a Varian Prepstar SD-1 semi-preparative system equipped (UV detection at 250 nm) using a Prep Microsorb–MWC18 column (250 X 41.4 mm; 6μ; 60 Å) with the following solvent system A= 5mM ammonium formate in water and B=acetonitrile and a gradient of 20%B to 44%B over 65 minutes with a flow rate of 20 ml/min.140 mg of the crude material above was dissolved in 10 ml methanol and filtered. The filtrate was divided into ten 1 ml aliquots for subsequent injection. Once completed the fractions corresponding to the correct mass were collected and freeze dried. Analytic HPLC-MS at the Bornmann lab was performed

on an Agilent Accurate-Mass 6200 TOF LC/MS system equipped with an Agilent LC1200 HPLC using a Varian Microsorb-MW C18 column (250 X 4.6 mm; 5 μ) with the following solvent gradient system: A= H_2O /0.1% TFA and B=acetonitrile/0.1% TFA. 10%B to 95%B over 30 min with a flow rate of 1ml/min

Nuclear magnetic resonance (NMR)spectra were recorded at the Bornmann lab on an IBM-Bruker Advance 500 (500 MHz for ^1H NMR and 125.76 MHz for ^{13}C NMR), spectrometers. The chemical structures of ibogamine, ibogaine and noribogaine were confirmed by ^1H and ^{13}C NMR via correlation spectroscopy (COSY) and heteronuclear correlation (HETCOR) analysis.

Pages 1-28: Proton, carbon, COSY and heteronuclear correlation HETCOR spectra of the noribogaine product of the preparative synthesis. The chemical structures of ibogamine, ibogaine and noribogaine were confirmed by ^1H and ^{13}C NMR including COSY and HETCOR analysis and indicate adequate separation of the compounds. The proton, carbon, COSY and HETCOR spectra of the noribogaine product are included here. The spectra confirm the presence of noribogaine and the absence of any other detectable contaminant structure, consistent with a purity > 99.9%.

NMR spectral data Noribogaine source C:

Chemical shifts (δ) are determined relative to MeOH-d_4 (referenced to 3.50ppm (δ) for ^1H NMR and 49.3 ppm (δ) for ^{13}C NMR). Proton-proton coupling constant (J) are given in Herz and splitting patterns are designated as singlet (s), doublet (d), triplet (t) quadruplet (q), multiplet or overlapped (m), and broad (br).

^1H NMR (500 MHz, MeOD) δ 7.29 (d, J = 8.6, 1H, H-12), 7.01 (d, J = 2.1, 1H, H-9), 6.85 (dd, J = 8.6, 2.3, 1H, H-11), 3.85 (dt, J = 13.4, 4.2, 1H, H-5β), 3.78 (s, 1H, H-21), 3.74 (dd, J = 13.4, 3.5, 1H, H-5α), 3.60 - 3.58 (m, 2H, H-3α, 3β), 3.52 (ddt, J = 10.3, 3.1, 1.5, 1H, H-16), 3.39 (ddd, J = 15.8, 11.5, 4.2, 1H, H-6β), 3.30 (dt, J = 17.7, 3.9, 1H, H-6α), 2.49 (t, J = 12.9, 1H, H-17β), 2.94 - 2.34 (m, 2H, H-14, 15β), 2.23 – 2.13 (m, 1H, H-20), 1.91 – 1.75 (m, 3H, H-17α, 19α, 19β), 1.61 – 1.53 (m, 1H, H-15α), 1.22 (t, J = 7.3, 3H, H-18).

^{13}C NMR (126 MHz, MeOD) δ 152.01 (C-10), 140.67 (C-2), 131.84 (C-13), 130.59 (C-8), 112.81 (C-11), 112.58 (C-12), 107.11 (C-7), 103.31 (C-9), 61.84 (C-21), 57.80 (C-

5), 52.31 (C-3), 40.64 (C-20), 36.72 (C-16), 32.83 (C-17), 30.43 (C-15), 27.52 (C-19), 25.57 (C-14), 19.70 (C-6), 12.17 (C-18).

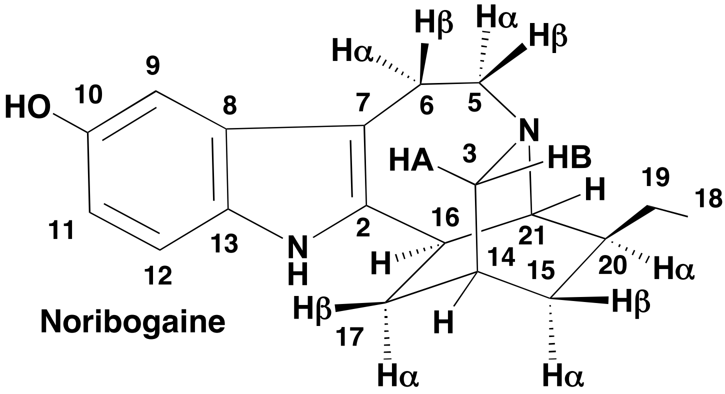

Pages 29-32: Analytic HPLC of products of the preparative HPLC. HPLC graphs of noribogaine (page 1) ibogaine (page 2) and ibogamine (page 3) and blank sample control (page 4). The graphs indicate adequate separation of the compounds and purity consistent with the NMR data.

Noribogaine source D:

Noribogaine HCl obtained from Slater & Frith Ltd (Wroxham Norwich, UK; donated by Phytostan Enterprises, Inc., Montreal, Quebec). Noribogaine HCl samples were analyzed using an AB Sciex 4000 QTrap (AB Sciex, Framingham, MA) hybrid triple quadrupole/linear ion trap liquid chromatograph-mass spectrometer (LCMS). Positive atmospheric chemical ionization was used as the ionization source. Nebulizer temperature was maintained at 450°C. Nebulizer or auxiliary gas was set at 40, curtain gas flow at 30, and the declustering potential was set to 100. The mass spectrometer was operated in single quadrupole mode, scanning from 200 to 400 da. Analytes were separated using a Shimadzu Prominence high performance (HPLC) system (Shimadzu Scientific Instruments, Columbia, MD) across a water to acetonitrile (ACN) gradient,

using 0.1% formic acid as an ion-pairing reagent. At the beginning of each run, the mobile phase was held at 15% ACN for 1 min, increased to 40% over 17 min, reduced back to 15% over 1 min, and held at 15% for 12 min. Flow was maintained at 100 µl/min. One µl of each sample was injected onto an Alltech (Grace Alltech, Deerfield IL) Alltima C18 reversed phase HPLC column (150 mm x 1 mm x5 µm).

Pages 1-2: HPLC-MS of noribogaine HCl (Kuehne lab, see above). The small impurity in this sample at 10 min is ibogamine; area of this ibogamine peak is .004 of the noribogaine peak by integration. No ibogaine was detected.

Page 3: Detailed analysis of the tail end of the noribogaine peak from the above HPLC-MS of noribogaine HCl. The tail contains only noribogaine and is due to residual elution of noribogaine.

18-MC:

18-MC was obtained from Obiter Research LLC, Champaign, IL, USA.

Page 1-3: Certificate of analysis 18-MC HCl, Obiter Research LLC, Champaign, IL, USA (Obiter). Overall impurity < 0.1%.

Pages 4-5: HPLC-MS (Kuehne lab;): A small fragment is present in the MS that is 16 higher than the mass of the compound. This is not an impurity in the sample, but is generated in the MS/HPLC system and appears to correspond to an extra oxygen on the molecular ion.

Comment regarding the possible effect of a laboratory contaminant:

Overall, it appears unlikely that a laboratory impurity in the iboga alkaloids utilized in this study could account for the finding that these compounds are not MOR agonists. Ibogaine, noribogaine and 18-MC, which produced similar effects on [^{35}S]GTPγS binding are the product of distinct manufacturing processes. Ibogaine was extracted from *T. iboga* root bark and noribogaine produced by demethylation of ibogaine, whereas 18-MC in contrast is a product of a total synthesis.

If a trace contaminant were to account for the finding that the iboga alkaloids evaluated in this study are not MOR agonists, it would have to be a high potency antagonist. A shared contaminant among the iboga alkaloids used in this study would have to be below a cutoff of detectable impurities, which for the sake of example is assumed to be 0.1%. If the iboga alkaloids were actually neutral with respect to their functional activity at the MOR, a 0.1% contaminant would have to have a potency of 13/1000 uM or 13 nM in order to produce an antagonist potency of 13 uM. An even greater potency would be required for a contaminant present at less than 0.1%. In this study the Ke of naltrexone was 0.96 nM. The potency of naloxone is on the order of one tenth that of naltrexone [2] Therefore, the contaminant, when present at 0.1%, would need to have potency close to that of naloxone; at lower levels of contamination, potencies higher than that of naloxone would be needed for the presently observed antagonist activity.

Furthermore, the above assumes that the iboga alkaloids are neutral with regard to a functional effect at the MOR, i.e., are not agonists (or antagonists). This would not negate the essential finding of the study, that these compounds, including noribogaine are not agonists. If the iboga alkaloids were really MOR agonists with the potency previously reported elsewhere [3], the hypothetical contaminant would have to overcome the putative agonist effect to truly artifact the main finding. We have calculated

(assuming an EC_{50} of 324 nM for agonist action as previously reported [3]) that this would require the presence of an antagonist (at 0.1%) that is at least 30 times more potent than naloxone. As noted above, it would also need to be present in 18-MC as well despite the very different synthetic process. All together, it appears unlikely that a laboratory impurity could account for the finding that ibogaine, noribogaine and 18-MC are not MOR agonists.

References

1. Gallagher CA, Hough LB, Keefner SM, Seyed-Mozaffari A, Archer S, et al. (1995) Identification and quantification of the indole alkaloid ibogaine in biological samples by gas chromatography-mass spectrometry. Biochem Pharmacol 49: 73-79.
2. Codd EE, Shank RP, Schupsky JJ, Raffa RB (1995) Serotonin and norepinephrine uptake inhibiting activity of centrally acting analgesics: structural determinants and role in antinociception. J Pharmacol Exp Ther 274: 1263-1270.
3. Pablo JP, Mash DC (1998) Noribogaine stimulates naloxone-sensitive [35S]GTPgammaS binding. Neuroreport 9: 109-114.
